# Stringy Corrections to Heterotic SU(3)-Geometry

**DOI:** 10.1007/s00220-026-05620-6

**Published:** 2026-04-04

**Authors:** Jock McOrist, Sebastien Picard

**Affiliations:** 1 Department of Mathematics, School of Science and Technology, University of New England, Armidale, 2351 Australia; 2 Department of Mathematics, University of British Columbia, 1984 Mathematics Road, Vancouver, BC Canada

## Abstract

We analyse the ${\alpha ^{\prime }\,}^2$ corrections to the supersymmetry algebra constructed by Bergshoeff–de Roo for heterotic compactifications on $\textrm{SU}(3)$ manifolds. The geometry is complex and conformally balanced. Starting from these supersymmetry constraints, we derive the equations of motions and find that the graviton equation contains an extraneous term which can be set to zero after gauge fixing. The curvature of the tangent bundle connection acquires a nonzero (0, 2) component and so does not satisfy the instanton equation, showing that the tangent bundle instanton condition does not persist beyond first order in ${\alpha ^{\prime }\,}$.

## Introduction

Non-Kähler geometry naturally arises in the study of the heterotic string on . In modern notation, the action for this theory is [1]^1^

1.1 $$\begin{aligned} \begin{aligned} S = \frac{1}{2} \int \textrm{d}^{10} x \sqrt{g} \, e^{-2\Phi } \left\{ R - \frac{1}{2} |H|^2 + 4 ({\partial }_m\Phi )^2 + \frac{{\alpha ^{\prime }\,}}{4} \textrm{tr}\hspace{2.0pt}|F|^2 -\right.&\left. \frac{{\alpha ^{\prime }\,}}{4} \textrm{tr}\hspace{2.0pt}|R^\textrm{H}|^2 + \cdots \right\} \\&+ \mathcal {O}({\alpha ^{\prime }\,}^3) ~, \end{aligned} \end{aligned}$$

where the $\cdots$ are terms involving fermions, which we omit. The bosonic fields are as follows: the graviton *g* is a metric tensor, the dilaton $\Phi$ is a scalar function, *F* is the curvature of a connection *A* for a $E_8 \times E_8$ or *SO*(32) gauge bundle *E*, and *H* is a three-form that satisfies the Bianchi identity

1.2 $$\begin{aligned} \textrm{d}H = \frac{{\alpha ^{\prime }\,}}{4} \textrm{tr}\hspace{2.0pt}F{\,\wedge \,}F - \frac{{\alpha ^{\prime }\,}}{4} \textrm{tr}\hspace{2.0pt}R^\textrm{H}{\,\wedge \,}R^\textrm{H}~. \end{aligned}$$

From the pair (*g*, *H*), we produce $R^\textrm{H}$ which is the curvature for a connection with torsion $\Theta ^\textrm{H}= \Theta ^\textrm{LC}+ \frac{1}{2}H$, where $\Theta ^\textrm{LC}$ is the Levi-Civita connection. *R* is the Ricci scalar for the metric on *X*. We have suppressed Newton’s constant. We note that (1.2) is an exact equality without further ${\alpha ^{\prime }\,}$ corrections; this condition is consistent with the Green-Schwarz anomaly cancellation mechanism [2].

We comment briefly on the origins of the action (1.1). Building on work by Chapline–Manton [3], Bergshoeff–de Roo [1, 4] constructed this action by studying the spacetime theory, supersymmetrising Lorentz–Chern–Simons terms, and constructing a supersymmetry algebra that leaves this action invariant. A profound and non-trivial test is that their results match the conditions from the sigma model point of view. The target space metric of the non-linear sigma model on spacetime  defines the kinetic terms of fields corresponding to the spacetime coordinates. The cancellation of the Weyl anomaly requires that *X* be a six-dimensional manifold, while unitarity implies it is endowed with a Riemannian metric. There are other fields, namely the Yang–Mills field and B-field, and together these satisfy the Bianchi identity, which is a requirement of a separate anomaly cancellation condition due to Green-Schwarz [2]. Quantisation of the sigma model promotes ${\alpha ^{\prime }\,}$ to a coupling constant and calculations proceed order-by-order in ${\alpha ^{\prime }\,}$; conformal invariance of the quantum theory is enforced by vanishing of the beta functions, which are computed via loop calculations at each order in perturbation theory. The results of Bergshoeff–de Roo match the conditions for vanishing beta functions [5, 6] and string scattering amplitudes [6, 7], and this was rechecked more recently in [8].

The departure point for the present work is the Bergshoeff–de Roo (BdR) supersymmetry algebra [1]. This leaves the action (1.1)—including both bosonic and fermionic fields—invariant. To be more precise, the supersymmetry algebra does not leave the action invariant as an exact equality, but generates extra terms of higher order ${\alpha ^{\prime }\,}^3$. So while the theory does not truncate to order ${\alpha ^{\prime }\,}^2$, it is understood order-by-order in a perturbative expansion.

We will apply the BdR algebra at order ${\alpha ^{\prime }\,}^2$ to the spacetime  and calculate the geometric constraints on the compact six-manifold *X*. We demand that there is a smooth ${\alpha ^{\prime }\,}\rightarrow 0$ limit; this is required by the sigma model description of the theory. Concretely this means geometric quantities cannot degenerate.^2^ We show the supersymmetry algebra implies $H=\mathcal {O}({\alpha ^{\prime }\,})$ and $\textrm{d}\Phi = \mathcal {O}({\alpha ^{\prime }\,})$ and so the geometry is underpinned by a Calabi–Yau manifold. This does not mean the metric is Kähler, as the ${\alpha ^{\prime }\,}, {\alpha ^{\prime }\,}^2, \cdots$ corrections generate torsion terms so that the Kähler form is no longer closed. Indeed, the non-Kähler corrections at first order in ${\alpha ^{\prime }\,}$ were studied by Hull [9] and Strominger [10].

The algebra of [1] requires *X* admit a global non-vanishing spinor $\eta$ annihilated by a covariant derivative $\nabla ^{\textrm{B}}\eta = 0$ where $\nabla ^{\textrm{B}}$ has some torsion *T*. This implies the manifold has *SU*(3) holonomy with respect to $\nabla ^{\textrm{B}}$ and there are globally well-defined spinor bilinears: a 3-form $\Omega$ and an almost complex structure *J* with $\nabla ^{\textrm{B}}J = 0$. That *J* is covariantly constant does not imply automatically the manifold is complex. In [10], it is shown that the torsion satisfies

$$ T = \textrm{i}({\partial }- {\overline{\partial }})\omega + N~, $$

with *N* the Nijenhuis tensor for *J*. The analysis of [10] holds to first order in ${\alpha ^{\prime }\,}$, where setting the gravition variation to zero implies $T=H$, setting the dilatino variation to zero implies $H^{0,3}=0$, and combining these equations yields $N=0$. To second order in ${\alpha ^{\prime }\,}$, the calculation is more involved as the dilatino variation implies $H^{0,3} + {\alpha ^{\prime }\,}P^{0,3} = 0$ while the gravitino variation implies

1.3 $$\begin{aligned} H=T + 2{\alpha ^{\prime }\,}P~,\qquad \textrm{where} \qquad P =\!- \frac{{\alpha ^{\prime }\,}}{16} e^{2\Phi } \nabla ^{-\,\dag } \Big (e^{-2\Phi } \big (\textrm{tr}\hspace{2.0pt}F{\,\wedge \,}F - \textrm{tr}\hspace{2.0pt}R^\textrm{H}{\,\wedge \,}R^\textrm{H}\big ) \Big ) ~, \end{aligned}$$

where $\nabla ^-$ is a metric connection with same torsion except with opposite sign to $\nabla ^\textrm{H}$ and $\dagger$ denotes the negative divergence $\nabla ^\dagger A = -\nabla ^i A_i$; we give a table of connections at the end of Sect. 1. Nonetheless we show that the Nijenhuis tensor still vanishes. The main point here is that integrability of the complex structure is not automatic from the perspective of supersymmetry, unlike the situation in some type II theories.

With the manifold being complex at order ${\alpha ^{\prime }\,}^2$, we can then show that for manifolds admitting a smooth ${\alpha ^{\prime }\,}\rightarrow 0$ limit that in fact ${\alpha ^{\prime }\,}P = \mathcal {O}({\alpha ^{\prime }\,}^3)$. This implies that the ${\alpha ^{\prime }\,}^2$-corrections in (1.3) are absent and

1.4 $$\begin{aligned} \begin{aligned} H&= \textrm{i}({\partial }-{\overline{\partial }})\omega + \mathcal {O}({\alpha ^{\prime }\,}^3)~. \\ \end{aligned} \end{aligned}$$

In addition, the complex manifold *X* admits a closed holomorphic volume form $\Omega$ whose norm is

1.5 $$\begin{aligned} \log \left\| \Omega \right\| _g =\! -2 \Phi + \mathcal {O}({\alpha ^{\prime }\,}^3)~, \end{aligned}$$

and further use of supersymmetry shows that the manifold is conformally balanced

$$\begin{aligned} \textrm{d}\left( e^{-2 \Phi } \omega ^2 \right) + \mathcal {O}({\alpha ^{\prime }\,}^3) = 0~. \end{aligned}$$

Thus the relations (1.4) and (1.5) between the fields *H* and $\Phi$ and the complex geometric structure $(X,\omega ,\Omega )$ are unchanged from first order in ${\alpha ^{\prime }\,}$ [9, 10] to second order, i.e. ${\alpha ^{\prime }\,}^2$. In principle, the quadratic terms in the ${\alpha ^{\prime }\,}$-expansion could have modified the complex geometry on *X*—see (2.10) for the equations on spinors with ${\alpha ^{\prime }\,}^2$-level terms—but these terms all cancel. Furthermore, writing the Bianchi identity in terms of complex geometry to ${\alpha ^{\prime }\,}^2$ implies

1.6 $$\begin{aligned} \begin{aligned} 2\textrm{i}{\overline{\partial }}{\partial }\omega&- \frac{{\alpha ^{\prime }\,}}{4} \left[ \textrm{tr}\hspace{2.0pt}F {\,\wedge \,}F - \textrm{tr}\hspace{2.0pt}R^\textrm{CH}{\,\wedge \,}R^\textrm{CH}\right] + \mathcal {O}({\alpha ^{\prime }\,}^3) = 0~. \end{aligned} \end{aligned}$$

This result is surprising since the Bianchi identity on $\textrm{d}H$ involves the curvature term $\textrm{tr}\hspace{2.0pt}R^\textrm{H}{\,\wedge \,}R^\textrm{H}$, but after combining the Bianchi identity with supersymmetry we derive an equation on complex manifolds involving the Chern curvature $\textrm{tr}\hspace{2.0pt}R^\textrm{CH}{\,\wedge \,}R^\textrm{CH}$.

The identity (1.6) suggests that perhaps the Chern connection is the right curvature for anomaly cancellation at higher orders. We only calculate here to second order in ${\alpha ^{\prime }\,}'$, and wonder whether this equality with Chern connection persists to next order. A geometric interpretation of this equation with Chern connection was given in [11] with follow-up work by [12]: the equation defines an elliptic complex on sections of an auxiliary vector bundle $Q \rightarrow X$ and this result is surveyed in Sect. 3.3.

An important deviation from the leading order in ${\alpha ^{\prime }\,}$ result concerns the connection $\Theta ^\textrm{H}$ on the tangent bundle appearing in the action (1.1) and Bianchi identity (1.2). The connection $\Theta ^H$ is known as the Hull connection and is given by $\Theta ^\textrm{H}= \Theta ^\textrm{LC}+ \frac{1}{2}H$. While the BdR supersymmetry algebra [1] implies that $\Theta ^\textrm{H}$ is a holomorphic instanton at lowest order in ${\alpha ^{\prime }\,}$, this condition is violated at the next order in ${\alpha ^{\prime }\,}$. Instead, it is required by the supersymmetry algebra that both the $R^{\textrm{H},0,2}$ and the trace of $R^\textrm{H}$ are proportional to $\textrm{d}H$; see (2.8) and (C.12) for the precise equation. This is an example of how quantum corrections can violate classical results, and as a consequence the action (1.1) is not supersymmetric if the connection $\Theta$ on the tangent bundle is an instanton.^3^

For completeness, we briefly mention a variety of other contexts where the Hull connection is central to higher corrections to string theory. For example, in [13], both the ten-dimensional 1-loop ${\alpha ^{\prime }\,}^3 R^4$ couplings and the resulting six-dimensional ${\alpha ^{\prime }\,}R^2$ couplings arising from type IIA compactified on *K*3 were analyzed. These corrections were only partially fixed by the one-loop string amplitude computation, while checking consistency with T-duality strongly suggested the couplings should be expressed in terms of curvatures built from torsionful connections rather than Levi-Civita. The structure of the six-dimensional effective action was then shown to be completely fixed using heterotic-type IIA duality, which removes the remaining ambiguities and shows the correct B-field dependence is naturally encoded using the Hull connection $\Theta ^\textrm{H}$ with appropriate averaging of Hull and Bismut connections in the CP-odd sector. The key point here is that duality with heterotic only worked provided the curvatures in the heterotic Bianchi identity were evaluated with the Hull connection.

These couplings were further tested at the level of off-shell supergravity in [14], where using off-shell $d=6$ $\mathcal {N} = (1,0)$ supergravity, a Gauss–Bonnet invariant was constructed and coupled to off-shell Einstein–Hilbert gravity. The Hull connection again played a central role in the construction of the invariants. These developments are examples of the role the Hull connection plays in organising ${\alpha ^{\prime }\,}$-corrected supergravity, even beyond the ${\alpha ^{\prime }\,}^2$ order studied here. The study and cancellation of the ${\alpha ^{\prime }\,}^2$ terms has appeared in other contexts. For example in [15] the BdR algebra is studied in a generalised geometry framework—a squaring of the supersymmetry variations via a generalised Lichnerowicz operator led to the equations of motion with the Bianchi identity evaluated with the Hull connection. The results in that paper appear consistent with the results we find here.

A natural consistency check is what is often called integrability: if we ‘square’ the supersymmetry variations (in essence, this means differentiating a second time, skew-symmetrizing, and manipulating the algebra), does the resulting curvature condition give any constraints at order ${\alpha ^{\prime }\,}^2$? In Sect. 4, we evaluate this curvature, together with the other spinor equations, and find the graviton equation of motion with an additional term proportional to the hessian of the dilaton. At first glance, this extraneous term looks problematic. The key observation is that for *SU*(3)-structure manifolds admitting a smooth ${\alpha ^{\prime }\,}\rightarrow 0$ limit, the dilaton is pure gauge up to order ${\alpha ^{\prime }\,}^3$ [16, 17]. By imposing the gauge-fixing condition described in [16, 17], the extraneous term vanishes identically.

In Sect. 5, we reformulate our results in a manner more accessible to a mathematical audience. We begin by assuming that the supersymmetry conditions–expressed as tensorial equations–hold identically. Under this assumption, we demonstrate that the equations of motion can be recovered purely from these tensor conditions using standard results from Hermitian geometry. This provides an independent consistency check of our analysis, and also a foundation for further mathematical exploration of the system. This approach reproduces the same gauge constraint on the dilaton, reinforcing the coherence of the geometric and physical formulations.

The outline for the paper is as follows. In Sect. 2 we review the Bergshoeff–de Roo supersymmetry algebra in [1] correct to ${\alpha ^{\prime }\,}^2$. We then apply this algebra to an *SU*(3) manifold. In Sect. 3 we derive conditions on the geometry of *X* order-by-order in ${\alpha ^{\prime }\,}$. In Sect. 4, we differentiate and skew-symmetrise the supersymmetry variations and find the equations of motion. In Sect. 5, we take stock mathematically: taking the tensor equations constructed previously in Sects. 2–3, we derive using hermitian geometry the equations of motion. We get identical equations to Sect. 4, providing a non-trivial consistency check. In Sect. 5 we conclude by commenting on connections to Ricci flow in heterotic theories.

### Equations of motion

In the remaining sections, to avoid notational clutter, we supress $\mathcal {O}({\alpha ^{\prime }\,}^3)$ unless possible confusion may arise and understand equality signs are to this order in the ${\alpha ^{\prime }\,}$ expansion. For example, $\nabla ^{\textrm{B}}_m \varepsilon + \mathcal {O}({\alpha ^{\prime }\,}^3) = 0$ is written as $\nabla _m^{\textrm{B}}\varepsilon = 0$.

We state the $d=10$ equations of motion and supersymmetry variations that leave (1.1) invariant. Let $B_{mn}$ be the local 2-form potential satisfying $H = \textrm{d}B + \frac{{\alpha ^{\prime }\,}}{4} ({\textrm{CS}}(A)-{\textrm{CS}}(\Theta ^\textrm{H}))$. The equations of motion come from (1.1) by varying the fields $\delta g_{mn}, \delta B_{mn}, \delta A, \delta \Phi$:

1.7 $$\begin{aligned}&\textrm{Ric}_{mn}+ 2 \nabla ^\textrm{LC}_m \nabla ^\textrm{LC}_n\Phi - \frac{1}{4} H_{mab} H_n{}^{ab} + \frac{{\alpha ^{\prime }\,}}{4} \Big ( \textrm{tr}\hspace{2.0pt}F_{mp} F_n{}^p - \textrm{tr}\hspace{2.0pt}R^\textrm{H}{}_{mp} R^\textrm{H}{}_n{}^{p} \Big ) = 0~, \end{aligned}$$

1.8 $$\begin{aligned}&\nabla ^\textrm{LC}{}^m(\textrm{e}^{-2\Phi } H_{mnp}) =0~, \end{aligned}$$

1.9

1.10 $$\begin{aligned}&R - 4(\nabla \Phi )^2 + 4 \nabla ^2 \Phi - \frac{1}{2}|H|^2 + \frac{{\alpha ^{\prime }\,}}{4} \big (\textrm{tr}\hspace{2.0pt}|F|^2 - \textrm{tr}\hspace{2.0pt}|R^\textrm{H}|^2\big )= 0~. \end{aligned}$$

Here *m*, *n* are real coordinates in $d=10$, which will later be specialised to the compact manifold *X*. The minus connection  acts as $\Theta ^- = \Theta ^\textrm{LC}- \frac{1}{2}H$ on tangent bundle indices. The Laplacian $\nabla ^2$, Ricci curvature $\textrm{Ric}$ and scalar curvature *R* are with respect to the Levi-Civita connection $\nabla ^\textrm{LC}$. This is the field basis of [1]. The components of a *p*-form *Q* are defined as

1.11 $$\begin{aligned} Q = \frac{1}{p!} Q_{m_1\cdots m_p} \textrm{d}x^{m_1} {\,\wedge \,}\cdots {\,\wedge \,}\textrm{d}x^{m_p}~, \quad |Q|^2 = \frac{1}{p!} Q_{m_1\cdots m_p} Q^{m_1\cdots m_p}~, \end{aligned}$$

and we will now often omit the wedge ‘${\,\wedge \,}$’ for compactness.

Next, let $S=S^+ \oplus S^-$ be a spinor bundle over the 10-dimensional spacetime so that we may add fermionic superpartners to the theory. To the bosonic action (1.1), we add the following fermionic fields: the gravitino $\psi \in \Gamma (S^+ \otimes T^*)$, the dilatino $\lambda \in \Gamma (S^- )$ and the gaugino $\chi \in \Gamma (S^+ \otimes \textrm{End} \, E)$. We will omit the explicit form of the inclusion of the fermions $(\psi ,\lambda ,\chi )$ into the action (1.1), but this can be found in [1]. The full action is such that it is invariant under the supersymmetry transformations, which upon setting fermions to zero take the form:1.12There are also supersymmetry transformations for the bosonic fields $(\delta g, \delta \Phi , \delta A, \delta B)$ which we list to leading order below in (2.1). Here $\varepsilon \in \Gamma (S^+)$ is a non-vanishing spinor generating the local supersymmetry algebra, and $P = - \frac{1}{4} e^{2 \Phi } \nabla ^-{}^\dagger (e^{-2 \Phi } dH)$. We are using the slash convention , where *Q* is a *p*-form and $\gamma ^m$ are the gamma matrices.

A background is supersymmetric if $\delta (\psi ,\lambda ,\chi ) = 0$. As usual, the background is bosonic and so to check supersymmetry we require that it remains bosonic. Hence, we just need to check the fermionic variations vanish. We give a more detailed summary of this result in the next section (Table 1).

**Table 1:** The connections that appear in heterotic compactifications on *SU*(3) manifolds

Connection	Symbol	Appearance	Properties
Levi-Civita	$\Theta ^\textrm{LC}$	*g*-EOM	Torsion-free, $\nabla ^\textrm{LC}J \ne 0$
Hull	$\Theta ^\textrm{H}= \Theta ^\textrm{LC}+ \frac{1}{2}H$	*g*-EOM, Bianchi	$\nabla ^\textrm{H}J \ne 0$
Minus	$\Theta ^- = \Theta ^\textrm{LC}- \frac{1}{2}H$	*F*-EOM, *P*	$\nabla ^- J \ne 0$
Bismut	$\Theta ^{\textrm{B}}= \Theta ^\textrm{LC}- \frac{1}{2}T$	Gravitino variation	$\nabla ^{\textrm{B}}J = 0$
Chern	$\Theta ^\textrm{CH}$	Complex geometry	$\nabla ^\textrm{CH}_{\bar{\mu }} = \bar{\partial }_{\bar{\mu }}$, $\nabla ^\textrm{CH}J =0$

Once we have proved the manifold is complex, we show ${\alpha ^{\prime }\,}P = \mathcal {O}({\alpha ^{\prime }\,}^3)$ and so there is in fact no difference between the minus connection and the Bismut connection. This may not be the case for ${\alpha ^{\prime }\,}^3$ corrections

## Supersymmetry Algebra and Action for $\textrm{SU}(3)$

### Bergshoeff–de Roo in $d=10$

We give a brief summary of the supersymmetry algebra and action constructed in BdR [1]. Notation is translated according to (B.1) and we refer the reader to the reference [1] for a more in-depth analysis.

In [1], building on [4], the authors construct an algebra and corresponding action invariant under supersymmetry, quadratic in both Yang–Mills and gravitational curvatures.^4^ A guiding principle for constructing the action is to treat the spin connection $\Theta$ on the tangent bundle as an $\textrm{SO}(9,1)$ gauge connection, symmetric in form with the Yang–Mills connection. This symmetry breaks down when coupling to gravity: the supersymmetry transformations act differently on the spin and gauge connections: it is a pseudo-symmetry, but nonetheless useful for finding an invariant action.

To zeroth order in ${\alpha ^{\prime }\,}$, the supersymmetry rules for coupled $d=10$ and YM fields are2.1where we have not written terms that are cubic in fermions. Here $\varepsilon$ is a Majorana–Weyl $d=10$ spinor, $e_m^a$ is the veilbein for the $d=10$ metric. The background is bosonic with all fermions set to zero and so to check that the background is supersymmetry all one needs to check is that the variations of the gravitino, dilatino and gaugino vanish, respectively $\delta _0\psi _m = \delta _0 \lambda = \delta _0 A_0=0$.

The Chapline-Manton coupling of Yang–Mills to gravity [3] requires an additional transformation for the *B*-field at first order in ${\alpha ^{\prime }\,}$ (in the original paper this was $g_{YM}^{-2}$). This transformation law reads

2.2 $$\begin{aligned} \delta _{\alpha ^{\prime }\,}B_{mn} = \frac{{\alpha ^{\prime }\,}}{2} \textrm{tr}\hspace{2.0pt}A_{[m} \delta _0 A_{n]} - \frac{{\alpha ^{\prime }\,}}{2} \textrm{tr}\hspace{2.0pt}\Theta ^\textrm{H}_{[m} \delta _0 \Theta ^\textrm{H}_{n]}~, \end{aligned}$$

and follows the guiding principle that there should be a symmetry between the *A* terms and the $\Theta$ terms. The total supersymmetry variation is the sum of these two contributions, so for example $\delta B = \delta _0 B + \delta _{\alpha ^{\prime }\,}B$.

The transformations (2.1)–(2.2) leave the action $\mathcal {L}(R) + \mathcal {L}(F^2) + \mathcal {L}(R^2)$ invariant, where

2.3 $$\begin{aligned} \mathcal {L}(R)= &   \frac{1}{2}e^{-2\Phi } \left\{ R - \frac{1}{12} H_{mnp} H^{mnp} + 4 ({\partial }_m\Phi )^2 \right\} + \mathrm{quadratic\,fermions} ~,\end{aligned}$$

2.4 $$\begin{aligned} \mathcal {L}(F^2)= &   \frac{{\alpha ^{\prime }\,}}{4} \textrm{tr}\hspace{2.0pt}|F|^2 + \mathrm{quadratic\,fermions} ~, \end{aligned}$$

2.5 $$\begin{aligned} \mathcal {L}(R^2)= &   \!- \frac{{\alpha ^{\prime }\,}}{4} \textrm{tr}\hspace{2.0pt}|R^\textrm{H}|^2 + \mathrm{quadratic\,fermions} ~. \end{aligned}$$

A key point made by [1] is that the field basis is chosen so that the supergravity multiplet is brought into a form that is analogous to the Yang–Mills multiplet.^5^ For example, the zeroth order supersymmetry variation of the *SO*(9, 1) connection is $\delta _0 \Theta _m^\textrm{H}= \delta _0(\Theta _m^\textrm{LC}+ \tfrac{1}{2} H_m)$, and this is calculated by applying (2.1) to the Levi-Civita connection, the B-field, and the Chern–Simons couplings. The result is analogous to $\delta _0 A$:

2.6 $$\begin{aligned} \delta _0 \Theta _m^\textrm{H}{}^{ab} = -\frac{1}{2}\bar{\varepsilon }\Gamma _m \psi ^{ab}~, \qquad \delta _0 \psi ^{ab} = \frac{1}{4} \Gamma ^{mn} \varepsilon R_{mn}{}^{ab} (\Theta ^\textrm{H})~, \end{aligned}$$

where $\psi ^{ab}$ is the super-partner of the connection $\Theta ^\textrm{H}$. It is important that this is not a fundamental field, but is instead a composite field, and can be thought of as the analogue of the gaugino. We cannot consistently truncate to zeroth order in ${\alpha ^{\prime }\,}$ due to the Bianchi identity and (2.2), and therefore we must continue the expansion and include the ${\alpha ^{\prime }\,}$-corrections.

The action $\mathcal {L}(R) + \mathcal {L}(F^2) + \mathcal {L}(R^2)$ on the surface looks like $G\times SO(9,1)$ Yang–Mills coupled to supergravity. However, the Lorentz term $\mathcal {L}(R^2)$ is really a gravitational interaction, and not a Yang–Mills interaction independent of the metric. The field $\Theta ^H$ is determined in terms of the underlying metric, *B*-field and dilaton. It gains an additional transformation law under supersymmetry as a result:

2.7 $$\begin{aligned} \delta _{\alpha ^{\prime }\,}\Theta ^\textrm{H}_m{}^{ab} = \frac{3}{2} \Gamma _{[m} X_{ab]}~, \qquad \qquad \delta _{\alpha ^{\prime }\,}\psi ^{ab} = \frac{3}{4} \Gamma _{cd} \varepsilon (\textrm{d}H)^{abcd} \end{aligned}$$

where $X_{ab} =\frac{{\alpha ^{\prime }\,}}{4} \textrm{tr}\hspace{2.0pt}(F_{ab} \chi ) - \frac{{\alpha ^{\prime }\,}}{4} \textrm{tr}\hspace{2.0pt}( R_{ab}(\Theta ^\textrm{H}) \psi )$. This is an example of how the pseudo-symmetry between *A* and $\Theta ^\textrm{H}$ is broken.

On an *SU*(3) manifold, the background being supersymmetric means $(\delta _0 + \delta _{\alpha ^{\prime }\,})\psi ^{ab} =0$ and this gives

2.8 $$\begin{aligned} \left( R^\textrm{H}_{\mu {\overline{\nu }}}{}^{\alpha {\overline{\beta }}}- \frac{1}{2} (\textrm{d}H)_{\mu {\overline{\nu }}}{}^{\alpha {\overline{\beta }}}\right) g^{\mu {\overline{\nu }}} + \mathcal {O}({\alpha ^{\prime }\,}^2) =0~, \qquad R^\textrm{H}_{{\overline{\mu }}{\overline{\nu }}}- \frac{1}{2} (\textrm{d}H)_{{\overline{\mu }}{\overline{\nu }}} + \mathcal {O}({\alpha ^{\prime }\,}^2) = 0~. \end{aligned}$$

We give an independent check of (2.8) using conformally balanced metrics and complex geometry in (C.12), which confirms that the variation of the superpartner to $\Theta ^\textrm{H}$ vanishing, $(\delta _0 + \delta _{\alpha ^{\prime }\,})\psi ^{ab} =0$, follows from the gravitino, dilaton and gaugino variations and does not give any new information. This is another echo of $\Theta ^\textrm{H}$ not being an independent degree of freedom, and that varying $\delta \Theta ^\textrm{H}$ in the action does not give new information either. Treating $\Theta ^\textrm{H}$ as a dependent field determined by (*g*, *H*) removes what are sometimes called the spurious degrees of freedom [11, 19–22].

We see from (2.8) that the Hermitian–Yang–Mills condition does not hold for the curvature on *TM*. Let us compare with what is known for the standard embedding; this is the case when *A* is taken to be $\Theta$, which implies $\textrm{d}H = 0$ and, when compactified to six dimensions, implies that $H=0$, $\Phi =const$ and the compact space is Calabi–Yau. It is known in string theory that explicit corrections at the standard embedding at order ${\alpha ^{\prime }\,}^3$ lead to violations of the Hermitian-Yang–Mills (HYM) condition for the connection on *TM* [18, 23, 24]. Away from the standard embedding, (2.8) already indicates this phenomenon occurs earlier on in the ${\alpha ^{\prime }\,}$-expansion.

To first order in ${\alpha ^{\prime }\,}$, the supersymmetry transformations are given by (2.1)–(2.2), which in turn imply the transformation rules (2.6) and (2.7) for the *SO*(9, 1) multiplet $(\Theta ^\textrm{H}, \psi )$. The action $\mathcal {L}(R)+\mathcal {L}(R^2)+\mathcal {L}(F^2)$ is invariant under (2.1), (2.2), and (2.6), but not under (2.7). In fact, (2.7) generates ${\alpha ^{\prime }\,}^2$ corrections to the action. Thus, while the supersymmetry algebra closes consistently at order ${\alpha ^{\prime }\,}$, the action itself is not invariant at this order–there is no consistent truncation to first order in ${\alpha ^{\prime }\,}$.

To cancel the ${\alpha ^{\prime }\,}^2$ terms generated by the supersymmetry algebra, a lemma is introduced in [1] that for variations of the Hull connection and its superpartner $\delta \Theta ^\textrm{H}$ and $\delta \psi ^{ab}$ in $\mathcal {L}(R) + \mathcal {L}(R^2)$, one finds $\delta \mathcal {L}= \delta _1 \mathcal {L}+ \delta _2 \mathcal {L}$, where $\delta _1 \mathcal {L}$ is proportional to the leading-order equations of motion, and $\delta _2 \mathcal {L}$ is proportional to $\textrm{d}H$. That one finds the lagrangian is modified by terms involving equations of motion is not surprising and a generic feature of field redefinitions; it is often utilised in studies of ${\alpha ^{\prime }\,}$-corrected supergravity to simplify the action, the symmetry and equations of motion. Under redefinitions, the equations of motion that derive form the Lagrangian and the supersymmetry variations will also change. In [1], the lemma gives the precise coefficients multiplying the equations of motion; and these allow one to read off how the supersymmetry variations are modified to soak up these extra terms.

When applied to the variation (2.7), the resulting ${\alpha ^{\prime }\,}^2$ terms fall into two categories: those proportional to the equations of motion and those involving the Bianchi identity. The former can be cancelled by introducing appropriate ${\alpha ^{\prime }\,}^2$ corrections to the supersymmetry transformations:2.9together with $\delta _{{\alpha ^{\prime }\,}^2} e_m{}^a, \delta _{{\alpha ^{\prime }\,}^2} B_{mn}, \delta _{{\alpha ^{\prime }\,}^2} \Phi$ which we have not written here but are in [1]. There are also additional ${\alpha ^{\prime }\,}^2$ terms added to the action which are quadratic in fermions; we do not write these as we only ever need the bosonic terms.

Just like at first order, if we evaluate the supersymmetry transformations (2.1), (2.2), and (2.9) the action is not invariant: there are ${\alpha ^{\prime }\,}^3$ terms that are not cancelled. BdR consider how to modify the action and supersymmetry transformations at ${\alpha ^{\prime }\,}^3$, but we do not discuss this here except to reiterate that the process does not truncate and one needs an order-by-order analysis.

Just as (2.1)–(2.2) induces the transformations (2.6), (2.7), the ${\alpha ^{\prime }\,}^2$ corrections (2.9) induce an ${\alpha ^{\prime }\,}^2$ correction to $\delta \Theta ^\textrm{H}, \delta \psi ^{ab}$ which will further modify how far the connection $\Theta ^\textrm{H}$ is being from an instanton.

### Bergshoeff–de Roo on *SU*(3)

We now compactify from the $d=10$ spacetime  to the 6-dimensional compact manifold *X*. For this, we assume the metric splits as  where the metric on  is the Minkowski metric, and write the generator of supersymmetry as $\varepsilon = \zeta \otimes \eta + c.c.$, where $\eta$ is a normalized positive chirality spinor on *X* and $\zeta$ is a spinor on Minkowski space . We assume that $(H,A,\Phi )$ have no components along . In this setup, we may treat the fields $(g,H,A,\Phi )$ as defined over the compact manifold *X*. Setting the fermionic variations (1.12) to zero gives the constraints on *X* required for a supersymmetric background.2.10where *m*, *n*, *k*, *a*, *b* are real coordinates on the compact 6-manifold *X* and

2.11 $$\begin{aligned} P_{mab} = \frac{1}{4} e^{2 \Phi } (\nabla ^-)^q (e^{-2 \Phi } \textrm{d}H)_{qmab}~, \end{aligned}$$

and

$$\begin{aligned} \nabla ^\pm {}_k V^q = \nabla ^{\textrm{LC}}{}_k V^q \pm \frac{1}{2}H_k{}^q{}_p V^p. \end{aligned}$$

This is to be supplemented by a Bianchi identity for *H* given in (1.2). Our strategy is to follow the approach of [25] and later [9, 10] in rewriting these equations in terms of conditions on the geometry of the manifold and vector bundle.

#### Gravitino variation

Let (*X*, *g*) be a compact six-dimensional Riemannian manifold equipped with a spinor bundle $S \rightarrow X$. Let $H \in \Omega ^3(X)$ and $\Phi \in C^\infty (X)$. Suppose $\eta \in \Gamma (S)$ is a nowhere vanishing positive chirality spinor satisfying $\eta ^\dagger \eta = 1$. In this section, we study how the gravitino equation affects the geometry of *X*. Setting the gravitino variation to zero gives the following gravitino equation:

2.12 $$\begin{aligned} \nabla ^{\textrm{LC}}{}_k \eta - \frac{1}{8} H_k{}^{ab} \gamma _{ab} \eta + \frac{{\alpha ^{\prime }\,}}{4} P_k{}^{ab} \gamma _{ab} \eta = 0~. \end{aligned}$$

As noticed by Candelas–Horowitz–Strominger–Witten [25] and Strominger [10], the existence of a normalized non-vanishing positive chirality spinor $\eta$ defines an almost-complex structure on (*X*, *g*) given by

$$\begin{aligned} J^k{}_\ell = \textrm{i}\eta ^\dagger \gamma ^k{}_\ell \eta ~, \end{aligned}$$

and satisfying $g(V,W) = g(JV,JW)$. In [25] and [10], this *J* is integrable and so *X* is a complex manifold. Our first goal is to understand whether *J* is integrable once the ${\alpha ^{\prime }\,}^2$-correction term in the supersymmetry equation is included. Equation (2.12) implies

$$\begin{aligned} \nabla ^{{\textrm{B}}} J = 0~, \end{aligned}$$

where the connection $\nabla ^{{\textrm{B}}}$ acts on vector fields by

2.13 $$\begin{aligned} \nabla ^{{\textrm{B}}}{}_k V^q = \nabla ^{\textrm{LC}}{}_k V^q - \frac{1}{2}H_k{}^q{}_p V^p + {\alpha ^{\prime }\,}P_k{}^q{}_p V^p, \quad V \in \Gamma (TX)~. \end{aligned}$$

The constraint $\nabla ^{{\textrm{B}}} J = 0$ gives a relation between (*X*, *g*, *J*) and the field *H*. The following general mathematical statement is well-known in the literature: see for example [26–28]. We give here the full proof to establish conventions.

##### Proposition 1

Let (*X*, *g*, *J*) be an almost-complex manifold, and let $\omega (V,W) = g(JV,W)$. Suppose $\nabla ^{{\textrm{B}}} J = 0$, where $\nabla ^{{\textrm{B}}}$ is a connection defined by a 3-form $T \in \Omega ^3(X)$ in the following way:

2.14 $$\begin{aligned} \nabla ^{{\textrm{B}}}{}_k V^i = \nabla ^{\textrm{LC}}{}_k V^i - \frac{1}{2}{T}_k{}^i{}_p V^p, \quad V \in \Gamma (TX)~. \end{aligned}$$

Then we have the relations

2.15 $$\begin{aligned} {T}_{ijk}&= 4N_{kij} + (d \omega )_{Ji,Jj,Jk} \end{aligned}$$

2.16 $$\begin{aligned} T&= \textrm{i}(\partial - \bar{\partial })\omega + N \end{aligned}$$

where $\partial \omega = (d \omega )^{2,1}$ and $\bar{\partial } \omega = (d \omega )^{1,2}$, and $N \in \Omega ^2(T^*X)$ is the Nijenhuis tensor.

##### Proof

In components, our conventions are

$$ \omega = \frac{1}{2}\omega _{ij} \textrm{d}x^{ij}, \quad J^i _p J^p _j =\!- \delta ^i _j, \quad \omega _{ij} = J^p _i g_{p j}, \quad \omega _{ij} = g_{Ji,j}~. $$

Here, and throughout the proof, we use the following short-hand notation:

$$\begin{aligned} J^k{}_i \beta _k {:}{=} \beta _{Ji}, \quad \beta \in \Omega ^1(X) \end{aligned}$$

so that for example $g_{Ji, j} {:}{=} J^k{}_i g_{kj}$ and $g(J \cdot , J \cdot ) = g(\cdot , \cdot )$ reads $g_{Ji,Jj}=g_{ij}$. The Nijenhuis tensor is defined by

$$\begin{aligned} N = \frac{1}{2}N_{pij} \, \textrm{d}x^p \otimes \textrm{d}x^{ij}~, \quad N_{pij} = g_{pk} N^k{}_{ij}~, \end{aligned}$$

where

2.17 $$\begin{aligned} N^p{}_{ij} = \frac{1}{4} \bigg ( J^k{}_i \nabla ^{\textrm{LC}}{}_k J^p{}_j + J^p{}_k \nabla ^{\textrm{LC}}{}_j J^k{}_i - (i \leftrightarrow j) \bigg )~. \end{aligned}$$

$\bullet$ The first step is to use $\nabla \omega = 0$ to compute $d \omega$ in terms of *T*. We start fromFrom $\nabla ^{{\textrm{B}}} \omega = 0$, we use the expression (2.14) to convert $\nabla ^{\textrm{LC}}$ to $\nabla ^{{\textrm{B}}}$ and obtainWe skew-symmetrize this expression and derive

$$\begin{aligned} (\textrm{d}\omega )_{kij} =\!-T_k{}^\ell {}_i \omega _{\ell j} -T_j{}^\ell {}_k \omega _{\ell i} -T_i{}^\ell {}_j \omega _{\ell k}~. \end{aligned}$$

Since $\omega _{ij} = - g_{i,Jj}$, this is

$$\begin{aligned} (\textrm{d}\omega )_{kij} =T_k{}^\ell {}_i g_{\ell , Jj} + T_j{}^\ell {}_k g_{\ell , Ji} + T_i{}^\ell {}_j g_{\ell , Jk}~. \end{aligned}$$

and so

2.18 $$\begin{aligned} (\textrm{d}\omega )_{kij} =T_{k, Jj,i} + T_{j,Ji,k} + T_{i,Jk,j}~. \end{aligned}$$

$\bullet$ The second step is to use $\nabla ^{{\textrm{B}}} J = 0$ to compute *N* in terms of *T*. We can use the expression (2.14) to derive the relation

2.19 $$\begin{aligned} \nabla ^{\textrm{LC}}{}_k J^p{}_j = \frac{1}{2}T_k{}^p{}_r J^r{}_j - \frac{1}{2}T_k{}^r{}_j J^p{}_r~. \end{aligned}$$

Substituting this into (2.17) gives

$$\begin{aligned} N^p{}_{ij} = \frac{1}{8} \bigg ( J^k{}_i (T_k{}^p{}_r J^r{}_j - T_k{}^r{}_j J^p{}_r) + J^p{}_k (T_j{}^k{}_r J^r{}_i - T_j{}^r{}_i J^k{}_r) - (i \leftrightarrow j) \bigg )~, \end{aligned}$$

where $T_j{}^{Jp}{}_j \cong J^p{}_k T_j{}^k{}_j$ and Nijenhuis becomes

2.20 $$\begin{aligned} N^p{}_{ij} = \frac{1}{4} (T_{Ji}{}^p{}_{Jj} - T_{Ji}{}^{Jp}{}_j + T_j{}^{Jp}{}_{Ji} + T_j{}^p{}_i )~. \end{aligned}$$

$\bullet$ We now derive (2.15). Returning to (2.18), we have

2.21 $$\begin{aligned} (\textrm{d}\omega )_{Ji,Jj,Jk} = T_{ Ji, j, Jk} + T_{i,Jj,Jk} + T_{ Ji, Jj, k}~. \end{aligned}$$

Lower an index on $N^p{}_{ij}$ in (2.20) and use $g_{kp} T_{Ji}{}^{Jp}{}_j = - T_{Ji,Jk,j}$

$$\begin{aligned} \begin{aligned} N_{kij}&= \frac{1}{4} (T_{Ji,k,Jj} + 2 T_{Ji,Jk,j} + T_{kij} )\\&=\!- \frac{1}{4} (T_{Ji,Jj,k} +T_{Ji,j,Jk} + T_{i,Jj,Jk} - T_{ijk} )\\&=\!- \frac{1}{4} \big ( (\textrm{d}\omega )_{Ji,Jj,Jk} - T_{ijk} \big )~. \end{aligned} \end{aligned}$$

We have used that $N^p{}_{ij} = - N^p{}_{ji}$. The identity (2.15) is then established.

$\bullet$ We now prove (2.16). Indeed, let $\{ e_\alpha \}_{\alpha =1}^3$ be a local frame for , where  is the $+i$ eigenspace of *J* so that e.g. $A_{J\alpha } = i A_\alpha$. Evaluating (2.18) in this frame gives

$$ (\textrm{d}\omega )_{\alpha \beta \gamma } =\! -3\textrm{i}T_{\alpha \beta \gamma }, \quad (\textrm{d}\omega )_{\alpha \bar{\beta } \gamma } =\!-\textrm{i}T_{\alpha \bar{\beta } \gamma }~. $$

Therefore $T_{\alpha \bar{\beta } \gamma } = (\textrm{i}\partial \omega )_{\alpha \bar{\beta } \gamma }$. Evaluating (2.20) in this frame gives

2.22 $$\begin{aligned} N_{\alpha \beta \gamma } = T_{\alpha \beta \gamma }~, \quad N_{\bar{\alpha } \beta \gamma } = N_{\alpha \bar{\beta } \gamma }= 0~. \end{aligned}$$

Combining these identities proves (2.16). Lastly, we note that (2.15) and (2.16) are consistent since the above equations imply $(d \omega )_{\alpha \beta \gamma } = -3i N_{\alpha \beta \gamma }$. $\square$

#### Supersymmetry at second order in ${\alpha ^{\prime }\,}$

We will apply Proposition 1 to the connection (2.13) that appears in the gravitino equation. This amounts to replacing *T* with $H_k{}^i{}_p - 2 {\alpha ^{\prime }\,}P_k{}^i{}_p$. We conclude this subsection with the following statement: suppose $\nabla ^{\textrm{B}}J = 0$ where $\nabla ^{\textrm{B}}$ is defined in (2.13). Then

2.23 $$\begin{aligned} H = \textrm{i}(\partial - \bar{\partial }) \omega + N + 2 {\alpha ^{\prime }\,}P~. \end{aligned}$$

We remark that the complex structure on the compact manifold *X* is not automatic from $\nabla ^{\textrm{B}}J = 0$, and more constraints are required to force $N=0$. To determine whether the Nijenhuis tensor vanishes, we include the dilatino equation.

#### Dilatino variation

The dilatino equation sets the following constraint2.24To extract information from it, we use the following identity noticed by Strominger.

##### Proposition 2

[10] Let (*X*, *g*, *J*) be an almost-complex 6-manifold with complex structure defined by a normalized non-vanishing positive chirality spinor $\eta$, so that $J^k{}_\ell = i \eta ^\dagger \gamma ^k{}_\ell \eta$. Suppose2.25for $Q \in \Omega ^3(X)$ and $\Phi \in C^\infty (X)$ and e.g. . Then

2.26 $$\begin{aligned} Q_{\mu \nu \lambda }= &   0 ~, \end{aligned}$$

2.27 $$\begin{aligned} g^{\mu \bar{\nu }} Q_{\mu \bar{\nu } \lambda }= &   \partial _\lambda \Phi ~, \end{aligned}$$

where Greek indices refer to a local frame $\{ e_\mu \}$ for .

##### Proof

The fact that *J* comes from $\eta$ means that $\gamma ^{\bar{\alpha }} \eta = 0$ and $\gamma _\alpha \eta = 0$. Here we use the following convention for indices: Greek indices (e.g. $\mu$, $\mu$) denote directions along  while Roman indices (e.g. *i*, *j*) denote directions spanning all of .

We start by applying $\gamma _\mu$ to (2.25). This gives

2.28 $$\begin{aligned} \frac{1}{6} Q_{\alpha \beta \delta } \{ \gamma _\mu , \gamma ^{\alpha \beta \delta } \} \eta + \frac{1}{2}\bigg ( Q_{\bar{\alpha } \beta \delta } \{ \gamma _\mu , \gamma ^{\bar{\alpha } \beta \delta } \} + Q_{\bar{\alpha } \bar{\beta } \delta } \{ \gamma _\mu , \gamma ^{\bar{\alpha } \bar{\beta } \delta } \} \bigg ) \eta =\! - \Phi _\alpha \gamma _\mu \gamma ^\alpha \eta ~, \end{aligned}$$

since $Q_{\bar{\alpha } \bar{\beta } \bar{\gamma }} \gamma ^{\bar{\alpha } \bar{\beta } \bar{\gamma }} \eta = 0$. We can apply the commutator identity (A.1) to verify the following identities:

2.29 $$\begin{aligned} \frac{1}{6} Q_{\alpha \beta \delta } \{ \gamma _\mu , \gamma ^{\alpha \beta \delta } \} \eta= &   Q_{\mu \alpha \beta } \gamma ^{\alpha \beta } \eta ~, \end{aligned}$$

2.30 $$\begin{aligned} \frac{1}{2}Q_{\bar{\alpha } \beta \delta } \{ \gamma _\mu , \gamma ^{\bar{\alpha } \beta \delta } \} \eta= &   2 Q_{\bar{\alpha } \beta \mu } \gamma ^{\bar{\alpha } \beta } \eta ~, \end{aligned}$$

2.31 $$\begin{aligned} Q_{\bar{\alpha } \bar{\beta } \delta } \{ \gamma _\mu , \gamma ^{\bar{\alpha } \bar{\beta } \delta } \} \eta= &   0~. \end{aligned}$$

Substituting these into (2.28), we obtain

2.32 $$\begin{aligned} Q_{\mu \alpha \beta } \gamma ^{\alpha \beta } \eta + 2 Q_{\bar{\alpha } \beta \mu } g^{\bar{\alpha } \beta } \eta =\! -2 \Phi _\mu \eta ~. \end{aligned}$$

The first term is in fact zero. Indeed, multiplying this equation through by $\gamma _\nu$ gives

$$ Q_{\mu \alpha \beta } [\gamma _\nu , \gamma ^{\alpha \beta }] \eta = 0~, $$

and from the commutator identity (A.1) we derive $Q_{\mu \alpha \beta } = 0$. Substituting this back into (2.28) completes the proof of the Proposition. $\square$

We now apply Proposition 2 to the dilatino equation (2.24). The result is

2.33 $$\begin{aligned} H_{\mu \nu \lambda } - 3 {\alpha ^{\prime }\,}P_{\mu \nu \lambda }= &   0~, \end{aligned}$$

2.34 $$\begin{aligned} g^{\mu \bar{\nu }} ( H_{ \bar{\nu } \mu \lambda } - 3 {\alpha ^{\prime }\,}P_{ \bar{\nu }\mu \lambda })= &   2\partial _\lambda \Phi \end{aligned}$$

where indices $\mu ,\nu ,\lambda$ denote a local frame $\{ e_\mu \}_{\mu =1}^3$ generating  with respect to the almost-complex structure *J*.

Next, we recall that $\nabla ^{{\textrm{B}}} J = 0$ implies (2.22), which combined with (2.23) reads

$$\begin{aligned} N_{\lambda \mu \nu } = H_{\lambda \mu \nu } - 2 {\alpha ^{\prime }\,}P_{\mu \nu \lambda }~. \end{aligned}$$

So we deduce that $\nabla ^{{\textrm{B}}} J = 0$ together with the dilatino equation (2.24) imply

2.35 $$\begin{aligned} N = N^{3,0} + N^{0,3}~, \qquad N_{\lambda \mu \nu } = {\alpha ^{\prime }\,}P_{\mu \nu \lambda }~. \end{aligned}$$

From here, we conclude that $N = \mathcal {O}({\alpha ^{\prime }\,}^2)$, recovering the result of Strominger [10] that the manifold is complex at linear order in ${\alpha ^{\prime }\,}$. In particular, *X* admits an integrable complex structure obtained by setting ${\alpha ^{\prime }\,}=0$. However, the almost-complex structure denoted $J^k{}_\ell = i \eta ^\dagger \gamma ^k{}_\ell \eta$ comes from a spinor $\eta$ satisfying the gravitino and dilatino equations with ${\alpha ^{\prime }\,}^2$-terms included, and in the following section we verify that the Nijenhuis tensor of this perturbed *J* vanishes at second order in ${\alpha ^{\prime }\,}$.

## Complex Geometry at Second Order

In the previous section, we started from the equations obtained by setting the fermionic variations to zero (2.10), and derived various constraints on the fields *g*, *H*, $\Phi$, and *J*. In the current section, we interpret the constraints as equations of complex geometry. We will find that *X* is a non-Kähler Calabi–Yau manifold with a conformally balanced metric satisfying a nonlinear constraint on $i \partial \bar{\partial } \omega$. This extends Strominger’s analysis [10] from linear order in ${\alpha ^{\prime }\,}$ to second order ${\alpha ^{\prime }\,}^2$.

### Zeroth order analysis

**Fig. 1:**
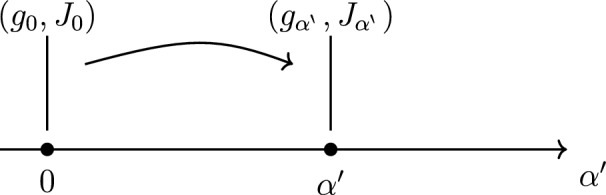
A family of tensors $(g_{\alpha ^{\prime }\,}, J_{\alpha ^{\prime }\,})$ over a real parameter ${\alpha ^{\prime }\,}$. The background $(g_0,J_0)$ is Kähler, while ${\alpha ^{\prime }\,}$ terms add non-Kähler corrections

Before delving into the equations of complex geometry to order ${\alpha ^{\prime }\,}^2$, we begin by rederiving the well-known result that at zeroth order in supersymmetry the manifold is complex with Kähler metric [25]. We take a family of our fields $(g,H,\Phi ,A,\eta )$ flowing in ${\alpha ^{\prime }\,}$, e.g.

$$ g = g_{\alpha ^{\prime }\,}= g^{(0)} + {\alpha ^{\prime }\,}g^{(1)} + {\alpha ^{\prime }\,}^2\,g^{(2)} + \cdots , \quad \eta = \eta _{{\alpha ^{\prime }\,}} = \eta ^{(0)} + {\alpha ^{\prime }\,}\eta ^{(1)} + \cdots ~, $$

and consequently the bilinear *J* also admits an ${\alpha ^{\prime }\,}$ expansion and so does its Nijenhuis tensor *N*. We assume $g^{(0)}$ is a metric tensor as well as $g_{\alpha ^{\prime }\,}$ at each value of the parameter ${\alpha ^{\prime }\,}$ (Fig. 1).

In this section, we prove that if the fields $(g,H,\Phi ,\eta )$ satisfy (2.10), then

3.1 $$\begin{aligned} H^{(0)} = 0~, \quad \Phi ^{(0)} = \textrm{const}, \quad N^{(0)} = 0~, \end{aligned}$$

and $(X,J^{(0)}, g^{(0)})$ is a complex Kähler manifold. We will often use in the following sections a consequence of this result, which is that

$$\begin{aligned} H = \mathcal {O}({\alpha ^{\prime }\,})~, \quad \nabla \Phi = \mathcal {O}({\alpha ^{\prime }\,})~. \end{aligned}$$

To see (3.1), send ${\alpha ^{\prime }\,}\rightarrow 0$ in (2.23), (2.34), (2.33), (1.2). We obtain

3.2 $$\begin{aligned} H^{(0)} -i (\partial -\bar{\partial })_{J^{(0)}} \omega ^{(0)} -N_{J^{(0)}}&= 0~, \end{aligned}$$

3.3 $$\begin{aligned} i \Lambda _{\omega ^{(0)}} H^{(0)} + 2 (\partial - \bar{\partial })_{J^{(0)}} \Phi ^{(0)}&= 0~, \end{aligned}$$

3.4 $$\begin{aligned} (H^{(0)})^{(3,0)}&= 0~, \end{aligned}$$

3.5 $$\begin{aligned} d H^{(0)}&= 0~. \end{aligned}$$

It follows that

$$\begin{aligned} (N^{(0)})^{(3,0)} = (H^{(0)})^{(3,0)} = 0~, \end{aligned}$$

and so $N_{J^{(0)}}=0$ since the Nijenhuis tensor is purely (3, 0)-type. We conclude that $J^{(0)}$ defines an analytic complex structure by the Newlander-Nirenberg theorem. Therefore

3.6 $$\begin{aligned} H^{(0)} = \textrm{i}(\partial - \bar{\partial })\omega ^{(0)}~, \quad (H^{(0)})_\mu {}^\mu {}_\lambda =\! - 2 \partial _\lambda \Phi ^{(0)} ~, \quad i \partial \bar{\partial } \omega ^{(0)}=0~. \end{aligned}$$

It is well-known [29, 30] that (3.6) implies that $\omega ^{(0)}$ is Kähler. To see this explicitly, we can take the trace of the exchange relation on Chern curvatures (C.10) to derive

$$\begin{aligned} (\textrm{i}\partial \bar{\partial } \omega )_\mu {}^{\mu \alpha }{}_{\alpha } = g^{\alpha \bar{\beta }} \partial _\alpha T_\mu {}^\mu {}_{\bar{\beta }} - g^{\alpha \bar{\beta }} \partial _{\bar{\beta }} T_\mu {}^\mu {}_\alpha + T_{\alpha \mu \bar{\nu }} T^{\mu \bar{\nu } \alpha }~, \quad T = \textrm{i}(\partial - \bar{\partial }) \omega ~, \end{aligned}$$

which is a general identity that holds on any hermitian manifold $(X,\omega )$. Substituting (3.6) into the above identity, we obtain

$$\begin{aligned} 4 g^{\alpha \bar{\beta }} \partial _\alpha \partial _{\bar{\beta }} \Phi ^{(0)} = \frac{1}{4} (H^{(0)})_{\alpha \mu \bar{\nu }} (H^{(0)})^{\mu \bar{\nu } \alpha } ~\ge ~ 0~. \end{aligned}$$

The maximum principle for elliptic equations on a compact manifold implies that $\Phi ^{(0)}$ is a constant and $H^{(0)}=0$. Therefore, at zeroth order the geometry is Kähler Calabi–Yau, and we will see that once ${\alpha ^{\prime }\,}$-corrections are included the geometry is perturbed to a non-Kähler Calabi–Yau structure.

### The manifold is complex at ${\alpha ^{\prime }\,}^2$

The first order analysis in [10] proves that $J^{(0)} + {\alpha ^{\prime }\,}J^{(1)}$ is integrable. We now show the Nijenhuis tensor vanishes to ${\alpha ^{\prime }\,}^2$. The only component to check is $N^{3,0}$ and its complex conjugate. From (2.35),

3.7 $$\begin{aligned} N_{\mu \nu \lambda } = \frac{{\alpha ^{\prime }\,}}{4} \Big ( (\nabla ^-)^\rho (\textrm{d}H)_{\rho \mu \nu \lambda } + (\nabla ^-)^{\overline{\rho }}(\textrm{d}H)_{{\overline{\rho }}\mu \nu \lambda }\Big )~. \end{aligned}$$

where $\{ e_\mu \}_{\mu =1}^3$ is a local frame for  over an almost-complex manifold *X*.

From [10], it is shown that $F^{0,2} = \mathcal {O}({\alpha ^{\prime }\,}^2)$. The curvature of the Hull connection satisfies $R^{\textrm{H}\,0,2} = \mathcal {O}({\alpha ^{\prime }\,})$ using the complex geometry valid at first order in ${\alpha ^{\prime }\,}$. We do this explicitly (3.9)–(3.10) below. Consequently,

$$\begin{aligned} \frac{{\alpha ^{\prime }\,}}{4} \textrm{tr}\hspace{2.0pt}F{\,\wedge \,}F = \frac{{\alpha ^{\prime }\,}}{4}( \textrm{tr}\hspace{2.0pt}F {\,\wedge \,}F )^{2,2} + \mathcal {O}({\alpha ^{\prime }\,}^3)~, \quad \frac{{\alpha ^{\prime }\,}}{4} \textrm{tr}\hspace{2.0pt}R^\textrm{H}{\,\wedge \,}R^\textrm{H}= \frac{{\alpha ^{\prime }\,}}{4}( \textrm{tr}\hspace{2.0pt}R^\textrm{H}{\,\wedge \,}R^\textrm{H})^{2,2} + \mathcal {O}({\alpha ^{\prime }\,}^2)~. \end{aligned}$$

The Bianchi identity (1.2) then implies $(\textrm{d}H)^{3,1} = \mathcal {O}({\alpha ^{\prime }\,}^2)$, and $(\textrm{d}H)^{4,0} = 0$ by dimensionality. Hence, (3.7) gives

$$\begin{aligned} N_{\mu \nu \lambda } = \mathcal {O}({\alpha ^{\prime }\,}^3)~. \end{aligned}$$

The manifold is complex at ${\alpha ^{\prime }\,}^2$ with $J = J^{(0)} + {\alpha ^{\prime }\,}J^{(1)} + {\alpha ^{\prime }\,}^2 J^{(2)}$ integrable.

Hence, (2.23) becomes

$$\begin{aligned} T = \textrm{i}({\partial }-{\overline{\partial }})\omega = H - 2{\alpha ^{\prime }\,}P~. \end{aligned}$$

### The constraint for $i \partial \bar{\partial } \omega$ at ${\alpha ^{\prime }\,}^2$

The Bianchi identity (1.2) repeated here is determined by anomaly cancellation and so unexpectedly not modified by ${\alpha ^{\prime }\,}$ corrections:

$$\begin{aligned} \textrm{d}H= \frac{{\alpha ^{\prime }\,}}{4}\Big (\textrm{tr}\hspace{2.0pt}F {\,\wedge \,}F - \textrm{tr}\hspace{2.0pt}R^\textrm{H}{\,\wedge \,}R^\textrm{H}\Big ) ~. \end{aligned}$$

What is corrected is the relation between ${\partial }{\overline{\partial }}\omega$ and the gauge bundle. We calculate this here. For this reason it is important that we refer to (1.2) as the Bianchi identity and not its expression in terms of $\omega$. We will derive

3.8 $$\begin{aligned} \begin{aligned} 2\textrm{i}{\overline{\partial }}{\partial }\omega&- \frac{{\alpha ^{\prime }\,}}{4} \left[ \textrm{tr}\hspace{2.0pt}F {\,\wedge \,}F - \textrm{tr}\hspace{2.0pt}R^\textrm{CH}{\,\wedge \,}R^\textrm{CH}\right] +\mathcal {O}({\alpha ^{\prime }\,}^3)=0~. \end{aligned} \end{aligned}$$

To start, we first improve on the previous subsection by showing $[\textrm{d}H]^{3,1} =0 + \mathcal {O}({\alpha ^{\prime }\,}^3)$. Write $\Theta ^\textrm{H}= \Theta ^\textrm{CH}+ S$ where *S* is some tensor

3.9 $$\begin{aligned} \begin{aligned} R^\textrm{H}&= R^\textrm{CH}+ \textrm{d}_\Theta S + S^2~, \\ \textrm{tr}\hspace{2.0pt}R^\textrm{H}{\,\wedge \,}R^\textrm{H}&= \textrm{tr}\hspace{2.0pt}R^\textrm{CH}{\,\wedge \,}R^\textrm{CH}+ \textrm{d}\left[ \textrm{tr}\hspace{2.0pt}\left( 2S{\,\wedge \,}R^\textrm{CH}+S{\,\wedge \,}\textrm{d}_{\Theta ^\textrm{CH}} S+ \frac{2}{3} S^3 \right) \right] ~. \end{aligned} \end{aligned}$$

In complex coordinates

3.10 $$\begin{aligned} R^{\textrm{H}\,0,2}{\,}^{\overline{\sigma }}{}_\rho = \textrm{d}x^{{\overline{\mu }}{\overline{\nu }}} \nabla ^\textrm{CH}_{\overline{\mu }}H_{\overline{\nu }}{}^{\overline{\sigma }}{}_\rho ~. \end{aligned}$$

$R^\textrm{H}$ fails to be a holomorphic instanton at first order in ${\alpha ^{\prime }\,}$, consistent with (2.8).

Split *S* into holomorphic and antiholomorphic components $S = {{\mathcal {S}}\,}+ {\overline{\mathcal {S}}\,}$, where from Sect. C.1 the only non-vanishing components are3.11Using $R^{\textrm{CH}\,0,2} = 0$ we find (to save space, we write $\Theta$ in this subsection to mean $\Theta ^\textrm{CH}$):we find

3.12 $$\begin{aligned} \begin{aligned} \textrm{tr}\hspace{2.0pt}R^\textrm{H}{\,\wedge \,}R^\textrm{H}- \textrm{tr}\hspace{2.0pt}R^\textrm{CH}{\,\wedge \,}R^\textrm{CH}&= 2( ({\partial }_\Theta + {\overline{\partial }}_\Theta ) {{\mathcal {S}}\,}^\nu {}_{\overline{\rho }}){\,\wedge \,}\big ( {\partial }_\Theta {\overline{\mathcal {S}}\,}^{\overline{\rho }}{}_\nu + {\overline{\partial }}_\Theta {\overline{\mathcal {S}}\,}^{\overline{\rho }}{}_\nu \big ) \\&\qquad + 2 {{\mathcal {S}}\,}^\nu {}_{\overline{\rho }}{\,\wedge \,}\big ( R^\textrm{CH}{}^{\overline{\rho }}{}_{\overline{\sigma }}{\overline{\mathcal {S}}\,}{}^{\overline{\sigma }}{}_\nu - R^\textrm{CH}{}^\sigma {}_\nu {\overline{\mathcal {S}}\,}{}^{\overline{\rho }}{}_\sigma \Big )~. \end{aligned} \end{aligned}$$

We can isolate the components that are not (2, 2) in the Bianchi:

$$\begin{aligned} {[}\textrm{tr}\hspace{2.0pt}R^\textrm{H}{\,\wedge \,}R^\textrm{H}]^{(1,3)} = 2{\overline{\partial }}\left( {{\mathcal {S}}\,}{}^\nu {}_{\overline{\rho }}{\,\wedge \,}{\overline{\partial }}_\Theta {\overline{\mathcal {S}}\,}{}^{\overline{\rho }}{}_\nu \right) ~, \qquad \qquad [\textrm{tr}\hspace{2.0pt}R^\textrm{H}{\,\wedge \,}R^\textrm{H}]^{(0,4)} = 0~. \end{aligned}$$

and so

$$\begin{aligned} {[}\textrm{d}H]^{1,3} = \frac{{\alpha ^{\prime }\,}}{2} {\overline{\partial }}\left( {{\mathcal {S}}\,}{}^\nu {}_{\overline{\rho }}{\,\wedge \,}{\overline{\partial }}_\Theta {\overline{\mathcal {S}}\,}{}^{\overline{\rho }}{}_\nu \right) = \mathcal {O}({\alpha ^{\prime }\,}^3) ~,\qquad \qquad [\textrm{d}H]^{0,4} = 0 ~. \end{aligned}$$

Now we find the ${\alpha ^{\prime }\,}^2$-corrected relation between $\omega$ and *F* using the Bianchi identity and the gravitino equation. Substitute (2.23) into (1.2), with terms manifestly (2, 2) on the left hand side,

3.13 $$\begin{aligned} \begin{aligned} \textrm{d}\textrm{d}^c\omega - \frac{{\alpha ^{\prime }\,}}{4} \textrm{tr}\hspace{2.0pt}(F {\,\wedge \,}F)&=\! -\frac{{\alpha ^{\prime }\,}}{4}\Big ( \textrm{tr}\hspace{2.0pt}R^\textrm{H}{\,\wedge \,}R^\textrm{H}\Big ) \\&\qquad \qquad + \frac{{\alpha ^{\prime }\,}^2}{8} \textrm{d}\Big (\nabla ^{{\textrm{B}}\,m} ( \textrm{tr}\hspace{2.0pt}F {\,\wedge \,}F - \textrm{tr}\hspace{2.0pt}R^\textrm{H}{\,\wedge \,}R^\textrm{H}\Big )_m \Big )~. \end{aligned} \end{aligned}$$

Using $$H=\mathcal {O}({\alpha ^{\prime }\,})$$:

$$ {\alpha ^{\prime }\,}\textrm{tr}\hspace{2.0pt}R^\textrm{H}{\,\wedge \,}\mathcal {R}^\textrm{H}- {\alpha ^{\prime }\,}\textrm{tr}\hspace{2.0pt}R^\textrm{CH}{\,\wedge \,}R^\textrm{CH}= \mathcal {O}({\alpha ^{\prime }\,}^3)~, $$

from (3.12).

In (4.31), we show that ${\alpha ^{\prime }\,}P = \mathcal {O}({\alpha ^{\prime }\,}^3)$ and so (3.13) becomes (3.8), which was to be established.

#### Remark

The nonlinear constraint on $i \partial \bar{\partial } \omega$, taken as the following stand-alone mathematical equation

3.14 $$\begin{aligned} 2\textrm{i}{\overline{\partial }}{\partial }\omega - \frac{{\alpha ^{\prime }\,}}{4} \left[ \textrm{tr}\hspace{2.0pt}F {\,\wedge \,}F - \textrm{tr}\hspace{2.0pt}R^\textrm{CH}{\,\wedge \,}R^\textrm{CH}\right] =0~, \end{aligned}$$

is given a differential geometric interpretation in [11], building on [19, 31]. Let $E \rightarrow X$ be a holomorphic bundle over a complex manifold *X* with *F* the Chern curvature of a hermitian metric on the bundle *E*. There is a differential operatorof the form

$$\begin{aligned} \bar{D}= \begin{bmatrix} {\overline{\partial }}&  - \frac{{\alpha ^{\prime }\,}}{4} \mathcal {F}^* &  \mathcal {T}- \frac{{\alpha ^{\prime }\,}}{4} \mathcal {R}\nabla \\ 0 &  {\overline{\partial }}_A &  \mathcal {F}\\ 0 &  0 &  {\overline{\partial }}\end{bmatrix}~, \end{aligned}$$

which satisfies $\bar{D}^2=0$ if and only if (3.14) holds. The definitions are:3.153.163.173.18The operator $\bar{D}$ does not quite define a holomorphic structure on the vector bundle *Q* due to the ${\alpha ^{\prime }\,}\mathcal {R}\nabla$ term. Given (3.14), then the differential operator $\bar{D}$ defines an elliptic complex

$$\begin{aligned} 0 \longrightarrow \Gamma (Q) \overset{\bar{D}}{\longrightarrow } \Omega ^{(0,1)}(Q) \overset{\bar{D}}{\longrightarrow } \Omega ^{(0,2)}(Q) \overset{\bar{D}}{\longrightarrow } \cdots ~. \end{aligned}$$

We refer to [12, 20] for cohomological calculations and physical interpretations of this complex. There is also a mathematical interpretation given in the language of generalized geometry [28, 32] of related equations of the form $i \partial \bar{\partial } \omega = c \langle F \wedge F \rangle$ where *c* is a bi-invariant symmetric pairing on the Lie algebra of the structure group of the bundle. For more on interactions between generalized geometry and heterotic string theory, see [33, 34].

### Holomorphic volume form

We have shown that the supersymmetry equations (2.10) lead to an integrable complex structure *J* at order ${\alpha ^{\prime }\,}^2$. Next, we investigate holomorphicity of a nowhere vanishing 3-form at order ${\alpha ^{\prime }\,}^2$. Consider the 3-form $\Psi \in \Omega ^3(X)$ determined by the positive chirality spinor $\eta$ given by

3.19 $$\begin{aligned} \Psi _{ijk} =\bar{\eta }^T \gamma _{ijk} \bar{\eta }~. \end{aligned}$$

One can check using $\gamma _7 \eta = \eta$ and $\gamma _\alpha \eta = 0$ in holomorphic directions that $\Psi \in \Omega ^{3,0}(X,\mathbb {C})$. The gravitino equation (2.12) implies

3.20 $$\begin{aligned} \quad \nabla ^{{\textrm{B}}} \Psi = 0~, \end{aligned}$$

where the connection $\nabla ^{{\textrm{B}}}$ is defined in (2.13). Since the connection $\nabla ^{{\textrm{B}}}$ also satisfies $\nabla ^{{\textrm{B}}} g = 0$ and $\nabla ^{{\textrm{B}}} J =0$, its holonomy is contained in the group *SU*(3).

$$\begin{aligned} \textrm{Hol}(\nabla ^{{\textrm{B}}}) \subseteq SU(3)~. \end{aligned}$$

The pair $(\omega ,\Psi )$ forms an *SU*(3)-structure, namely

$$\begin{aligned} \omega \wedge \Psi = 0~, \quad i \Psi \wedge \bar{\Psi } = \frac{\omega ^3}{3!}~, \quad |\Psi |_g = 1~. \end{aligned}$$

We note that neither $\omega$ nor $\Psi$ are closed. The constant norm 3-form $\Psi$ will not be a holomorphic volume form at order ${\alpha ^{\prime }\,}^2$, but we will arrange that $\Omega = e^f \Psi$ solves $\bar{\partial } \Omega = 0$ for some conformal factor $e^f$.

Let us rewrite the gravitino equation for $\nabla ^{\textrm{LC}} \eta$ in a different way for later use. Using $\gamma _\alpha \eta = 0$ in holomorphic directions and $H^{3,0}={\alpha ^{\prime }\,}P^{3,0} = \mathcal {O}({\alpha ^{\prime }\,}^3)$, from the gravitino equation (2.12) we derive

$$\begin{aligned} \nabla ^{\textrm{LC}}{}_\alpha \eta = \frac{1}{4} H_\alpha {}^{\mu \bar{\nu }} g_{\mu \bar{\nu }} \eta - \frac{{\alpha ^{\prime }\,}}{2} P_\alpha {}^{\mu \bar{\nu }} g_{\mu \bar{\nu }} \eta ~, \end{aligned}$$

in holomorphic indices. We next substitute the dilatino equation (2.34).

3.21 $$\begin{aligned} \nabla ^{\textrm{LC}}{}_\alpha \eta = \frac{1}{2} (\partial _\alpha \Phi ) \eta + \frac{{\alpha ^{\prime }\,}}{4} P_\alpha {}^{\mu \bar{\nu }} g_{\mu \bar{\nu }} \eta ~. \end{aligned}$$

We can also combine the dilatino equation (2.34) with the locking relation for *H* given in (2.23), knowing now that the Nijenhuis tensor is zero, to see

3.22 $$\begin{aligned} (i \partial \omega )_{\mu }{}^\mu {}_{\lambda } - {\alpha ^{\prime }\,}P_{\mu }{}^\mu {}_{\lambda } =\! -2\partial _\lambda \Phi ~. \end{aligned}$$

Combining (3.21) and (3.22) yields the identity

3.23 $$\begin{aligned} \nabla ^{\textrm{LC}}{}_\alpha \eta =\!-\frac{1}{4} (i \partial \omega )_{\mu }{}^\mu {}_{\alpha } \eta ~. \end{aligned}$$

With (3.23), we can now compute the covariant derivative of the 3-form $\Psi$ defined in (3.19).

$$\begin{aligned} \nabla ^{\textrm{LC}}{}_{\bar{\alpha }} \Psi _{ijk} = 2 \bar{\eta }^T \gamma _{ijk} \nabla ^{\textrm{LC}}{}_{\bar{\alpha }} \bar{\eta } =\! -\frac{1}{2}(i \bar{\partial } \omega )_{\mu }{}^\mu {}_{\bar{\alpha }} \Psi _{ijk}~. \end{aligned}$$

The Levi-Civita connection induced on top forms $\Psi \in \Omega ^{3,0}(X)$ is

$$\begin{aligned} \nabla ^{\textrm{LC}}{}_{\bar{\alpha }} \Psi _{ijk} = \partial _{\bar{\alpha }} \Psi _{ijk} - \Gamma ^{\textrm{LC}}{}_{\bar{\alpha }}{}^\mu {}_\mu \Psi _{ijk} = \partial _{\bar{\alpha }} \Psi _{ijk}- \frac{1}{2} (i \bar{\partial } \omega )_{\bar{\alpha }}{}^\mu {}_\mu \Psi _{ijk}. \end{aligned}$$

Therefore

$$\begin{aligned} \partial _{\bar{\alpha }} \Psi _{ijk} =\! - (\textrm{i}\bar{\partial } \omega )_{\mu }{}^\mu {}_{\bar{\alpha }} \Psi _{ijk}~. \end{aligned}$$

Substituting (3.22) gives

$$\begin{aligned} \partial _{\bar{\alpha }} \Psi _{ijk} = (2 \partial _{\bar{\alpha }} \Phi + {\alpha ^{\prime }\,}P_\mu {}^\mu {}_{\bar{\alpha }}) \Psi _{ijk}~. \end{aligned}$$

As expected, $\Psi$ is holomorphic to zeroth order in ${\alpha ^{\prime }\,}$, but to higher order it must be corrected by setting $\Omega = e^f \Psi$ and solving $\bar{\partial } \Omega = 0$ for a suitable conformal factor. For this, we use that in fact ${\alpha ^{\prime }\,}P = \mathcal {O}({\alpha ^{\prime }\,}^3)$; this will be derived later on in (4.29). Terms involving ${\alpha ^{\prime }\,}P$ may now be dropped, and so we define

$$\begin{aligned} \Omega = \exp \left( -2 \Phi \right) \Psi , \quad \Psi _{ijk} = \bar{\eta }^T \gamma _{ijk} \bar{\eta }~, \end{aligned}$$

so that at order ${\alpha ^{\prime }\,}^2$ we produced $\Omega \in \Omega ^{3,0}(X,\mathbb {C})$ which is nowhere vanishing with $\bar{\partial } \Omega = 0$. Taking the norm of $\Omega$ gives

3.24 $$\begin{aligned} \log \left\| \Omega \right\| _g =\! -2 \Phi ~. \end{aligned}$$

As $(X,\omega )$ is a complex hermitian manifold with holomorphic volume form $\Omega$ but with $d \omega \ne 0$, it is a non-Kähler Calabi–Yau threefold.

### Conformally balanced equation

The supersymmetry equations on spinors have produced a pair $(\omega , \Omega )$, where $\omega$ is a hermitian metric on a complex manifold and $\Omega$ is a holomorphic volume form. We now derive the conformally balanced relation $d( \left\| \Omega \right\| _g \omega ^2)=0$. Substituting ${\alpha ^{\prime }\,}P = \mathcal {O}({\alpha ^{\prime }\,}^3)$ into (3.22) gives

$$\begin{aligned} \frac{1}{2} (\textrm{i}\partial \omega )_{\mu }{}^\mu {}_{\lambda } =\! -\partial _\lambda \Phi ~, \end{aligned}$$

and thus by (C.2) we obtain

$$\begin{aligned} \textrm{d}\left( \exp (-2 \Phi ) \omega ^2 \right) = 0~. \end{aligned}$$

We can write this conformal factor $e^{-2 \Phi }$ in terms of the holomorphic volume form $\Omega$ by substituting (3.24), and the result is $d ( \left\| \Omega \right\| _g \omega ^2 )=0$.

The mathematical significance of the conformally balanced equation becomes apparent when the gaugino equation (2.10) is included, which states $g^{\mu \bar{\nu }} F_{\mu \bar{\nu }}=0$. The triple $(\omega ,\Omega ,F)$ then solves

$$\begin{aligned} d ( \left\| \Omega \right\| _g \omega ^2 )=0, \quad F \wedge \omega ^2= 0~. \end{aligned}$$

The (conformal) closedness of $\omega ^2$ is what allows the numerical pairing $[\left\| \Omega \right\| _g \omega ^2] \cdot c_1(S)$ to be defined on subsheaves $S \subseteq E$, and this is what allows a well-defined notion of slope stability as a criterion for the non-Kähler Donaldson–Uhlenbeck–Yau theorem [35–37]: if $[\left\| \Omega \right\| _g \omega ^2] \cdot c_1(S) < 0$ for all strict torsion-free coherent subsheaves $S \subseteq E$, then there exists a solution to $F \wedge \omega ^2= 0$.

## Integrability and the Equations of Motion

Supersymmetry of the spacetime is equivalent to the vanishing of the fermionic variations given in (2.10). As a consistency check, one can act with a derivative on (2.10), skew-symmetrise and extract the resulting torsionful curvature equation to obtain an integrability condition. After some manipulation, this reveals a connection between the spinors in (2.10) and the bosonic equations of motion.

### Preliminaries

Let $\varepsilon$ be the ten-dimensional Majorana Weyl spinor. Recall, there is the torsion of the Bismut connection *T*, its relation to *H* and a new three-form ${\hat{H}}$ that appears in the dilatino variation:

4.1 $$\begin{aligned} T_{mab} = H_{mab} - 2{\alpha ^{\prime }\,}P_{mab} = {\hat{H}}_{mab} + {\alpha ^{\prime }\,}P_{mab}~, \qquad {\hat{H}}_{mab} = H_{mab} - 3 {\alpha ^{\prime }\,}P_{mab}~. \end{aligned}$$

The gravitino variation implies a spinor covariantly constant with respect to the *T* connection. Take a second covariant derivative of the gravitino variation with respect to Levi-Civita:

4.2 $$\begin{aligned} \nabla _n\nabla _m \varepsilon - \frac{1}{8} \nabla _n T_{mab} \gamma ^{ab} \varepsilon - \frac{1}{64}T_{mab} T_{ncd} \gamma ^{ab} \gamma ^{cd} \varepsilon = 0~, \end{aligned}$$

where in this section unless otherwise noted $\nabla = \nabla ^\textrm{LC}$. Antisymmetrise using ${[}\nabla _m,\nabla _n] = \frac{1}{4} R_{mnab} \gamma ^{ab}~,$ where $\gamma ^a$ are the gamma matrices and use the first line of (A.2) to give

4.3 $$\begin{aligned} \textrm{d}x^m {\,\wedge \,}\textrm{d}x^n \left( R_{mnab} - \nabla _{[m} T_{n]ab} + \frac{1}{2}T_{[m|ac} T_{n]}{}^c{}_b \right) \gamma ^{ab} \varepsilon = 0~. \end{aligned}$$

This result can be derived in a second way using Cartan’s formulation. Write the gravitino variation as

4.4 $$\begin{aligned} \nabla ^{\textrm{B}}_m \varepsilon = {\partial }_m \varepsilon + \frac{1}{4} \Theta ^{\textrm{B}}_{m\,ab} \gamma ^{ab}~, \qquad \Theta ^{\textrm{B}}_m{}^a{}_b = \Theta ^\textrm{LC}_m{}^a{}_b - \frac{1}{2}T_m{}^a{}_b~. \end{aligned}$$

The integrability condition comes from skew-symmetrising the derivatives

4.5 $$\begin{aligned} {[} \nabla ^{\textrm{B}}_m, \nabla ^{\textrm{B}}_n] \varepsilon = \frac{1}{4} R^{\textrm{B}}_{mnab} \gamma ^{ab}\varepsilon =0~, \qquad R^{\textrm{B}}= \textrm{d}\Theta ^{\textrm{B}}+ \Theta ^{\textrm{B}}{\,\wedge \,}\Theta ^{\textrm{B}}~. \end{aligned}$$

Now, as $\Theta ^{\textrm{B}}= \Theta ^\textrm{LC}- \frac{1}{2}T$ we have

$$ R^{\textrm{B}}= R^\textrm{LC}- \frac{1}{2}\textrm{d}_{\nabla ^\textrm{LC}} T +\frac{1}{4}T {\,\wedge \,}T~, $$

where $\nabla ^\textrm{LC}$ acts only on the spinor indices labelled *a*, *b*. Evaluating this gives

4.6 $$\begin{aligned} R^{\textrm{B}}_{ab} = \frac{1}{2}\textrm{d}x^m {\,\wedge \,}\textrm{d}x^n \left( R_{mnab}^\textrm{LC}- \nabla ^\textrm{LC}_{[m} T_{n]ab} + \frac{1}{2}T_{[m|a}{}^c T_{n]cb} \right) ~, \end{aligned}$$

which when put in (4.5) gives (4.3) as promised.

As tempting as it is, we cannot identify the terms in parenthesis with zero in (4.3). Instead, multiply (4.3) by $\gamma ^n$ and use $\gamma ^n \gamma ^{ab} = g^{na} \gamma ^b - g^{nb} \gamma ^a + \gamma ^{nab}$ via the middle line of (A.1) to give

4.7 $$\begin{aligned} \begin{aligned} \frac{1}{2}R^{\textrm{B}}_{mnab} \gamma ^n\gamma ^{ab} \varepsilon =&\left( \textrm{Ric}_{mn} - \frac{1}{4}T_{mab} T_{n}{}^{ab} +\frac{1}{2}\nabla ^{\textrm{LC}\,p} T_{pmn}\right) \gamma ^n\varepsilon \\&\qquad \quad +\frac{1}{4} \left( \nabla ^\textrm{LC}_m T_{nab} - \nabla ^\textrm{LC}_n T_{mab}- T_{ma}{}^c T_{bnc} \right) \gamma ^{nab} \varepsilon = 0~. \end{aligned} \end{aligned}$$

Note we have used $H^p{}_{pq} = 0$, $\textrm{Ric}_{mn} = R_{mpn}{}^p$ and the Bianchi identity for the Riemann curvature $R_{m[nab]}=0$ has been used. Our goal is to massage the term proportional to $\gamma ^n \varepsilon$ to be as close to the equations of motion as possible.

We need the covariant derivative of the dilatino equation, which is the second line of (2.10). Taking a derivative and using $\nabla ^{\textrm{B}}\varepsilon = 0$, we find

$$ \left( 2 \nabla ^{\textrm{B}}_m \nabla _n \Phi \gamma ^n\varepsilon - \frac{1}{6}\nabla ^{\textrm{B}}_m {\hat{H}}_{nab} \gamma ^{nab}\right) \varepsilon = 0~. $$

In components, this becomes

4.8 $$\begin{aligned} \begin{aligned} \left( 2 \nabla _m \nabla _n \Phi + H_m{}^p{}_n \nabla _p \Phi \right) \gamma ^n\varepsilon = \frac{1}{6}\left( \nabla _m {\hat{H}}_{nab} - \frac{3}{2} H_{mn}{}^p H_{abp} \right) \gamma ^{nab}\varepsilon ~, \end{aligned} \end{aligned}$$

where we use ${\hat{H}}$ in (4.1).

Finally, multiply the gaugino equation  by $F_{pn}\gamma ^n$ to obtain

4.9 $$\begin{aligned} F_{pn} F_{ab} \gamma ^{nab} \varepsilon + 2 F_p{}^a F_{an} \gamma ^n \varepsilon = 0~. \end{aligned}$$

Hence,

4.10 $$\begin{aligned} \begin{aligned}&\frac{1}{12} \textrm{tr}\hspace{2.0pt}(F {\,\wedge \,}F)_{mnab} \gamma ^{nab} \varepsilon = F_m{}^a F_{na}\gamma ^n \varepsilon ~, \qquad \textrm{where}~~\\&\qquad \qquad \frac{1}{2}\textrm{tr}\hspace{2.0pt}(F{\,\wedge \,}F)_{mnab} = F_{mn} F_{ab} - F_{ma} F_{nb} - F_{mb} F_{an} ~. \end{aligned} \end{aligned}$$

Note that

4.11 $$\begin{aligned} - e^{2\Phi } \nabla ^p (e^{-2\Phi } H_{pmn} ) \gamma ^n \varepsilon = - \nabla ^p H_{pmn} \gamma ^n \varepsilon + 2(\nabla ^p \Phi ) H_{pmn} \gamma ^n \varepsilon ~. \end{aligned}$$

With this collection of results in hand we return to the integrability condition (4.7). Using (4.1), (4.10) and

$$\begin{aligned}&(\textrm{d}H)_{mnab} = \nabla _m H_{nab} - \nabla _n H_{abm} + \nabla _a H_{bmn} - \nabla _b H_{mna}~, \\&(\textrm{d}H)_{mnab} \gamma ^{nab} = (\nabla _m H_{nab} - 3 \nabla _n H_{abm} )\gamma ^{nab}~, \end{aligned}$$

gives

$$\begin{aligned} \begin{aligned}&\left( \textrm{Ric}_{mn} - \frac{1}{4}H_{mab} H_{n}{}^{ab}\right) \gamma ^n\varepsilon -\frac{1}{4}\left( H_{ma}{}^c H_{bnc} \right) \gamma ^{nab} \varepsilon \\&\quad =- \frac{1}{12} \left( \nabla ^\textrm{LC}_m T_{nab} - 3\nabla ^\textrm{LC}_n T_{abm} + 2\nabla ^\textrm{LC}_m {\hat{H}}_{nab} +2{\alpha ^{\prime }\,}\nabla ^\textrm{LC}_m P_{nab} \right) \gamma ^{nab}\\&\qquad \qquad +{\alpha ^{\prime }\,}\nabla ^p P_{pmn} \gamma ^n \varepsilon - H_{pmn} ( \nabla ^p \Phi ) \gamma ^n \varepsilon - \frac{1}{2}e^{2\Phi } \nabla ^p (e^{-2\Phi } H_{pmn} ) \gamma ^n \varepsilon \\ \end{aligned} \end{aligned}$$

Using (4.8), together with (4.20) that ${\alpha ^{\prime }\,}P \propto {\alpha ^{\prime }\,}\textrm{d}^\dag (\textrm{d}H) + \mathcal {O}({\alpha ^{\prime }\,}^3)$, so that $\textrm{d}^\dag P= \mathcal {O}({\alpha ^{\prime }\,}^3)$, this becomes

$$\begin{aligned} \begin{aligned}&\left( \textrm{Ric}_{mn} + 2 \nabla _m \nabla _n \Phi - \frac{1}{4}H_{mab} H_{n}{}^{ab}\right) \gamma ^n\varepsilon + \frac{1}{2}e^{2\Phi } \nabla ^p (e^{-2\Phi } H_{pmn} ) \gamma ^n \\&\quad =\!- \frac{1}{12} (\textrm{d}T)_{mnab} \gamma ^{nab} - \frac{{\alpha ^{\prime }\,}}{6} \nabla _m P_{nab} \gamma ^{nab} ~. \end{aligned} \end{aligned}$$

Using $$T = H - 2{\alpha ^{\prime }\,}P$$ and (4.9)–(4.10)

4.12 $$\begin{aligned} \begin{aligned}&\left( \textrm{Ric}_{mn} + 2\nabla _m \nabla _n \Phi - \frac{1}{4}H_{mab} H_{n}{}^{ab} + \frac{{\alpha ^{\prime }\,}}{4}\textrm{tr}\hspace{2.0pt}F_m{}^a F_{na} \right) \gamma ^n\varepsilon + \frac{1}{2}e^{2\Phi } \nabla ^p (e^{-2\Phi } H_{pmn} ) \gamma ^n\\&\quad =\!- \frac{1}{12}\Big ( (\textrm{d}H)_{mnab} - \frac{{\alpha ^{\prime }\,}}{4} (\textrm{tr}\hspace{2.0pt}F^2)_{mnab} \Big ) \gamma ^{nab} \varepsilon + \frac{{\alpha ^{\prime }\,}}{6} \Big ( (\textrm{d}P)_{mnab} -\nabla ^\textrm{LC}_m P_{nab} \Big ) \gamma ^{nab} \varepsilon ~. \end{aligned} \end{aligned}$$

The first line is converging on the graviton equation of motion. The *H* equation of motion is there. The second line is converging on the Bianchi identity plus terms manifestly ${\alpha ^{\prime }\,}^2$.

We now add and subtract terms to get the graviton equation of motion and evaluate the heterotic Bianchi identity (1.2):

4.13 $$\begin{aligned} \begin{aligned}&\left( \textrm{Ric}_{mn} + 2\nabla _m \nabla _n \Phi - \frac{1}{4}H_{mab} H_{n}{}^{ab} + \frac{{\alpha ^{\prime }\,}}{4}\textrm{tr}\hspace{2.0pt}F_m{}^a F_{na} - \frac{{\alpha ^{\prime }\,}}{4}\textrm{tr}\hspace{2.0pt}R^\textrm{H}{}_m{}^a R^\textrm{H}{}_{na} \right) \gamma ^n\varepsilon \\&+ \frac{1}{2}e^{2\Phi } \nabla ^p (e^{-2\Phi } H_{pmn} ) \gamma ^n \varepsilon =\! - \frac{{\alpha ^{\prime }\,}}{4}\textrm{tr}\hspace{2.0pt}\left( R^\textrm{H}{}_m{}^a R^\textrm{H}{}_{na} \gamma ^n\varepsilon - \frac{1}{12} (R^\textrm{H}{\,\wedge \,}R^\textrm{H})_{mnab} \gamma ^{nab} \varepsilon \right) \\&~~~ + \frac{{\alpha ^{\prime }\,}}{6} \Big ( (\textrm{d}P)_{mnab} -\nabla ^\textrm{LC}_m P_{nab} \Big ) \gamma ^{nab} \varepsilon ~.\\ \end{aligned} \end{aligned}$$

The left hand side contains the graviton and *H* equations of motion. The second line includes a term which would vanish if $R^\textrm{H}$ were traceless. To see this, note that if we treated $R^\textrm{H}$ in the same way as *F*, then we could apply (4.9)–(4.10) and this line would vanish. Since $R^\textrm{H}$ is not an instanton [1] (see (2.8) and (3.10)), we are left with the second line. The final line are all ${\alpha ^{\prime }\,}^2$ corrections.

### $\textrm{SU}(3)$ manifold

Thus far we have not specified the dimension of the compactification. To make progress we specialise (4.13) to *SU*(3). This means we can introduce a complex structure, as we proved earlier that the manifold is complex. The ten-dimensional spinor is written as $\varepsilon = \zeta \otimes \eta + \mathrm{c.c.}$, where $\eta$ is a Weyl spinor of $SO(6)\cong SU(4)$. As usual we focus just on the internal manifold and discard the $d=4$ spacetime component.

#### *H* and *F* equations of motion

We comment on the *H* and *F* equations of motion. First, we derive from the gaugino variation  the *F* equation of motion. For an *SU*(3) manifold, the gaugino equation reads

4.14 $$\begin{aligned} F_{\mu {\overline{\nu }}} \gamma ^{\mu {\overline{\nu }}} \eta = 0~,\qquad \omega ^{\mu {\overline{\nu }}} F_{\mu {\overline{\nu }}} = 0~, \end{aligned}$$

where we use (A.3). The gaugino is understood to come multiplied by an ${\alpha ^{\prime }\,}$ and so we keep terms up to order ${\alpha ^{\prime }\,}$. This means we can differentiate with respect to the Bismut connection and it will kill $\omega$ leaving

4.15 $$\begin{aligned} \begin{aligned} \omega ^{\mu {\overline{\nu }}} \nabla ^{\textrm{B}}_\rho F_{\mu {\overline{\nu }}}&= \omega ^{\mu {\overline{\nu }}}\left( {\partial }^A_\rho F_{\mu {\overline{\nu }}} -\Gamma ^\textrm{LC}_\rho {}^\sigma {}_\mu F_{\sigma {\overline{\nu }}} + \frac{1}{2}H_\rho {}^\sigma {}_\mu F_{\sigma {\overline{\nu }}} + H_\rho {}^{\overline{\sigma }}{}_{\overline{\nu }}F_{\mu {\overline{\sigma }}} \right) \\&= \textrm{i}\nabla ^{{\textrm{B}}\, {\overline{\nu }}} (e^{-2\Phi } F_{{\overline{\nu }}\rho }) = 0~, \end{aligned} \end{aligned}$$

where $\nabla ^A_\mu F_{\mu {\overline{\nu }}}$ is the gauge covariant derivative and we use the Bianchi identity ${\partial }_A F = 0$. We have replaced the torsion *T* by *H* as the difference is ${\alpha ^{\prime }\,}^2$, keeping in mind the gaugino comes with an implicit ${\alpha ^{\prime }\,}$, this difference is really ${\alpha ^{\prime }\,}^3$. We also use $2\nabla ^{\overline{\sigma }}\Phi = H^\rho {}_\rho {}^{\overline{\sigma }}+ \mathcal {O}({\alpha ^{\prime }\,}^2)$. This means supersymmetry implies the gaugino equation of motion and we can use it in the following.

Second, we show that the *H* equations of motion follow directly from the supersymmetry conditions. Using that $\star \beta ^{2,1} = \textrm{i}\beta ^{2,1} - \beta _\mu {}^\mu {\,\wedge \,}\omega$, we find

4.16 $$\begin{aligned} \star T = - e^{2{\hat{\Phi }}} \textrm{d}(e^{-2{\hat{\Phi }}} \omega )~, \qquad \hat{\Phi }= \Phi + \frac{{\alpha ^{\prime }\,}^2}{32} (\textrm{tr}\hspace{2.0pt}|F|^2 - \textrm{tr}\hspace{2.0pt}|R^\textrm{H}|^2)~, \end{aligned}$$

where we use $T = \textrm{d}^c\omega = H-2{\alpha ^{\prime }\,}P$, the dilatino equation (2.34) applied to *T*, and the trace of *P* (4.31) calculated below. Here we introduced the scalar function $\hat{\Phi }$ which is defined by (4.16). Hence,

4.17 $$\begin{aligned} \begin{aligned} e^{-2{\hat{\Phi }}} \star H&= 2{\alpha ^{\prime }\,}e^{-2{\hat{\Phi }}} \star P - \textrm{d}(e^{-2{\hat{\Phi }}} \omega ). \\ \end{aligned} \end{aligned}$$

From (4.20) below we see that

$$ {\alpha ^{\prime }\,}\textrm{d}(e^{-2{\hat{\Phi }}} \star P) = {\alpha ^{\prime }\,}^2 (\textrm{d}^\dag )^2 (\textrm{tr}\hspace{2.0pt}F{\,\wedge \,}F - \textrm{tr}\hspace{2.0pt}R^\textrm{H}{\,\wedge \,}R^\textrm{H}) -2 \textrm{d}{\hat{\Phi }} {\,\wedge \,}\star {\alpha ^{\prime }\,}P = \mathcal {O}({\alpha ^{\prime }\,}^3) $$

and so finally noting that $e^{-2{\hat{\Phi }}} H = e^{-2\Phi } H + \mathcal {O}({\alpha ^{\prime }\,}^3)$ we end up with

4.18 $$\begin{aligned} \textrm{d}\left( e^{-2 \Phi } \star H \right) =0~, \end{aligned}$$

which is the *H* equation of motion up to and including ${\alpha ^{\prime }\,}^2$. It follows directly from supersymmetry.

#### Preliminary results on *P* and *H*

We derive some results to be used in the following sections. In the following we assume supersymmetry holds. We have already shown to zeroth order Sect. 3.1 that the *SU*(3) manifold is complex, Kähler with Ricci-flat metric. In fact, the Bergshoeff–de Roo algebra has (2.8) which to zeroth order in ${\alpha ^{\prime }\,}$ shows $R^\textrm{H}$ satisfies a Yang–Mills equation and that $R^{\textrm{H}\,0,2} = \mathcal {O}({\alpha ^{\prime }\,})$ (shown directly using (2.6) for an *SU*(3) manifold in the same way as *F*; or using (3.10) with $H=\mathcal {O}({\alpha ^{\prime }\,})$). Together with Yau’s theorem, this implies that the metric is Ricci flat to zeroth order: $\textrm{Ric}_{mn} = \mathcal {O}({\alpha ^{\prime }\,})$.

For any *p*-form *Q*, we write $Q_m = \frac{1}{(p-1)!} Q_{m n_2\cdots n_p} \textrm{d}x^{n_2\cdots n_p}$ and its divergence satisfies:

4.19 $$\begin{aligned} \begin{aligned} \nabla ^{{\textrm{B}}\, m} Q_m&= g^{mn} \left( \nabla ^\textrm{LC}_n Q_m + \frac{1}{2}H_n{}^p{}_m Q_p \right) = \nabla ^{\textrm{LC}\,m} Q_m + \mathcal {O}({\alpha ^{\prime }\,}^2) \\&= \left( \nabla ^\textrm{CH}_\mu Q_{\overline{\nu }}+ \nabla ^\textrm{CH}_{\overline{\nu }}Q_\mu \right) g^{\mu {\overline{\nu }}} + \mathcal {O}({\alpha ^{\prime }\,}^2)~, \end{aligned} \end{aligned}$$

where we use the dilatino equation (2.34) with (2.11) and that $\textrm{d}H = \mathcal {O}({\alpha ^{\prime }\,})$.

The tensor *P* in (2.11) simplifies using (4.19)

4.20 $$\begin{aligned} \begin{aligned} {\alpha ^{\prime }\,}P&= \frac{{\alpha ^{\prime }\,}}{4} e^{2\Phi } \nabla ^{{\textrm{B}}\,m}\left( e^{-2\Phi } (\textrm{d}H)_m \right) \\&= \frac{{\alpha ^{\prime }\,}^2}{8} \nabla ^m \left( \textrm{tr}\hspace{2.0pt}F_m {\,\wedge \,}F - \textrm{tr}\hspace{2.0pt}R^\textrm{H}{}_m {\,\wedge \,}R^\textrm{H}\right) + \mathcal {O}({\alpha ^{\prime }\,}^3) =\!- \frac{{\alpha ^{\prime }\,}}{4} \textrm{d}^\dag \textrm{d}H ~. \end{aligned} \end{aligned}$$

Due to the ${\alpha ^{\prime }\,}^2$, $\nabla ^m$ can be any connection and the dilatino equation $\nabla _\rho \Phi = \frac{1}{2}H_\rho {}^\tau {}_\tau + \cdots = \mathcal {O}({\alpha ^{\prime }\,})$ allowed us to eliminate $e^{\Phi }$ from *P*. Furthermore, $\textrm{d}^\dag P = \mathcal {O}({\alpha ^{\prime }\,}^3)$.

We can further simplify the divergence of the Chern class

4.21 $$\begin{aligned} \begin{aligned} \frac{{\alpha ^{\prime }\,}^2}{2}&\nabla ^{{\textrm{B}}\,m}\Big (\textrm{tr}\hspace{2.0pt}(F{\,\wedge \,}F)_m\Big ) = {\alpha ^{\prime }\,}^2 \textrm{tr}\hspace{2.0pt}\left( \nabla _A^{m} F{\,\wedge \,}F_m + F{\,\wedge \,}\nabla _A^{m} F_m\right) \\&={\alpha ^{\prime }\,}^2 \textrm{tr}\hspace{2.0pt}( \textrm{d}_A F_m{\,\wedge \,}F^m) +\mathcal {O}({\alpha ^{\prime }\,}^3) =\frac{{\alpha ^{\prime }\,}^2}{2} ( {\overline{\partial }}- {\partial }) \textrm{tr}\hspace{2.0pt}(F^\rho {\,\wedge \,}F_\rho ) ~, \end{aligned} \end{aligned}$$

where we use $\textrm{d}_A F = 0$, the *F* equation of motion (imposed via the gaugino variation), the dilatino equation implies any $\nabla \Phi$ can be dropped as they are ${\alpha ^{\prime }\,}^3$. Repeat for the corresponding term in $R^\textrm{H}$, which is straightforward due to $\nabla ^m R^\textrm{H}_m = \mathcal {O}({\alpha ^{\prime }\,})$. This follows both directly from the BdR supersymmetry algebra (2.8); and also hermitian geometry with the conformally balanced equation: (C.11) with (5.11), (3.10).

Consequently,

4.22 $$\begin{aligned} {\alpha ^{\prime }\,}P = \frac{{\alpha ^{\prime }\,}^2}{8} ({\overline{\partial }}-{\partial })\Big ( \textrm{tr}\hspace{2.0pt}(F^\rho {\,\wedge \,}F_\rho ) - \textrm{tr}\hspace{2.0pt}(R^{\textrm{H}\,\rho } {\,\wedge \,}R^\textrm{H}_\rho ) \Big ) ~. \end{aligned}$$

We need some results for $\textrm{d}H$ and its trace. The trace of $(\textrm{d}H)^{3,1}$:

4.23 $$\begin{aligned} \begin{aligned} ( \textrm{d}H)^{3,1}&= \frac{1}{3!} (\textrm{d}H)_{\mu \nu \rho {\overline{\sigma }}} \textrm{d}x^{\mu \nu \rho {\overline{\sigma }}} = {\overline{\partial }}H^{3,0} + {\partial }H^{2,1} \\&= \frac{1}{3!} \left( - \nabla _{\overline{\sigma }}H_{\mu \nu \rho } + \nabla _\mu H_{\nu \rho {\overline{\sigma }}} + \nabla _\nu H_{\rho \mu {\overline{\sigma }}} + \nabla _\rho H_{\mu \nu {\overline{\sigma }}}\right) \textrm{d}x^{\mu \nu \rho {\overline{\sigma }}} ~. \end{aligned} \end{aligned}$$

Hence,

4.24 $$\begin{aligned} \begin{aligned} (\textrm{d}H)_{\mu \nu \rho }{}^\rho&= \left( - \nabla ^\rho H_{\mu \nu \rho } + \nabla ^{\overline{\sigma }}H_{\mu \nu {\overline{\sigma }}} - \nabla _\mu H{}_\nu {}^\rho {}_{\rho }{} + \nabla _\nu H{}_\mu {}^\rho {}_{\rho } \right) ~. \end{aligned} \end{aligned}$$

The dilatino equation (2.34) with (4.1) implies $\nabla _\nu H_\mu {}^\rho {}_\rho = 2\nabla _\nu \nabla _\mu \Phi + 3{\alpha ^{\prime }\,}\nabla _\nu P_\mu {}^\rho {}_\rho$. Hence, with (4.31),

$$\begin{aligned} \nabla _\nu H_\mu {}^\rho {}_\rho - \nabla _\mu H_\nu {}^\rho {}_\rho = 0~. \end{aligned}$$

The dilatino equation (2.34) implies $\hat{H}_{\mu \nu \rho } = H_{\mu \nu \rho } - 3{\alpha ^{\prime }\,}P_{\mu \nu \rho } = 0$, and so with ${\alpha ^{\prime }\,}P = \mathcal {O}({\alpha ^{\prime }\,}^3)$ we find $H_{\mu \nu \rho } = \mathcal {O}({\alpha ^{\prime }\,}^3)$. The Levi-Civita connection acts byand so $H_{\mu \nu \rho } = \mathcal {O}({\alpha ^{\prime }\,}^3)$ does not imply $\nabla _{\overline{\sigma }}H_{\mu \nu \rho }=\mathcal {O}({\alpha ^{\prime }\,}^3)$. Using (C.4), we see thatTogether with the *H* equation of motion and an application of dilatino equation, we find

4.25 $$\begin{aligned} \begin{aligned} (\textrm{d}H)_{\mu \nu \rho }{}^\rho&= -2\nabla ^\rho H_{\mu \nu \rho } + \nabla ^p H_{\mu \nu p} = -2\nabla ^\rho H_{\mu \nu \rho } + 2(\nabla ^p \Phi ) H_{\mu \nu p}\\&= 2H^{\rho {\overline{\lambda }}}{}_{[\mu } H_{\nu ] \rho {\overline{\lambda }}} ~. \end{aligned} \end{aligned}$$

Now, we compute the trace of $(\textrm{d}H)^{2,2} = {\overline{\partial }}H^{2,1} + {\partial }H^{1,2}$. Its useful to record the form in components:

$$ (\textrm{d}H)_{\mu \nu {\overline{\sigma }}{\overline{\rho }}}= \left( \nabla _{\overline{\sigma }}H_{\mu \nu {\overline{\rho }}} - \nabla _{\overline{\rho }}H_{\mu \nu {\overline{\sigma }}} \right) + \left( \nabla _\mu H_{\nu {\overline{\sigma }}{\overline{\rho }}} - \nabla _\nu H_{\mu {\overline{\sigma }}{\overline{\rho }}} \right) ~. $$

The trace is

4.26 $$\begin{aligned} \begin{aligned} (\textrm{d}H)_{\nu }{}^\nu {}_{\mu {\overline{\sigma }}}&= \nabla _{\overline{\sigma }}H_\mu {}^\nu {}_{\nu } -\nabla _\mu H_{\overline{\sigma }}{}^\nu {}_{\nu } - \nabla ^\nu H_{\nu \mu {\overline{\sigma }}} + \nabla ^{\overline{\nu }}H_{{\overline{\nu }}\mu {\overline{\sigma }}} ~. \end{aligned} \end{aligned}$$

The dilatino equation (2.34) and (4.31) implies

$$\begin{aligned} \begin{aligned} \nabla _{\overline{\sigma }}H_\mu {}^\rho {}_\rho&= 2\nabla _{\overline{\sigma }}\nabla _\mu \Phi + 3{\alpha ^{\prime }\,}\nabla _{\overline{\sigma }}P_\mu {}^\rho {}_\rho = \nabla _{\overline{\sigma }}\nabla _\mu \left( 2 \Phi + \frac{{\alpha ^{\prime }\,}^2}{8} \left( \textrm{tr}\hspace{2.0pt}|F|^2 - \textrm{tr}\hspace{2.0pt}|R|^2 \right) \right) ~,\\ \end{aligned} \end{aligned}$$

with $\nabla _\mu H_{\overline{\sigma }}{}^\rho {}_\rho$ following by complex conjugation.

Putting this into (4.26):

4.27 $$\begin{aligned} \begin{aligned} (\textrm{d}H)_{\nu }{}^\nu {}_{\mu {\overline{\sigma }}}&= 4\nabla _\mu \nabla _{\overline{\sigma }}\Phi + \mathcal {O}({\alpha ^{\prime }\,}^2)~. \end{aligned} \end{aligned}$$

It is also useful to trace the Bianchi identity (1.2). Using $F^{0,2}=F_\mu {}^\mu = \mathcal {O}({\alpha ^{\prime }\,}^2)$ with $R^\textrm{H}{}^{0,2} = R^\textrm{H}{}_\mu {}^\mu =\mathcal {O}({\alpha ^{\prime }\,})$ and (4.10),

4.28 $$\begin{aligned} \begin{aligned} (\textrm{d}H)_{\nu }{}^\nu {}= (\textrm{d}H)_{\nu }{}^\nu {}_{\mu {\overline{\sigma }}} \textrm{d}x^{\mu {\overline{\sigma }}}&= \frac{{\alpha ^{\prime }\,}}{2} (\textrm{tr}\hspace{2.0pt}F^\nu {\,\wedge \,}F_{\nu } - \textrm{tr}\hspace{2.0pt}R^\textrm{H}{}^\nu {\,\wedge \,}R^\textrm{H}_\nu ) + \mathcal {O}({\alpha ^{\prime }\,}^2)~, \end{aligned} \end{aligned}$$

Finally, we put (4.22), (4.27) and (4.28) to evaluate ${\alpha ^{\prime }\,}P$:

4.29 $$\begin{aligned} \begin{aligned} {\alpha ^{\prime }\,}P&= \frac{{\alpha ^{\prime }\,}}{4} ({\overline{\partial }}-{\partial }) (\textrm{d}H){}_\nu {}^\nu \\&= {\alpha ^{\prime }\,}\,{\overline{\partial }}{\overline{\partial }}{\partial }\Phi + \mathrm{c.c.}+ \mathcal {O}({\alpha ^{\prime }\,}^3) = \mathcal {O}({\alpha ^{\prime }\,}^3)~. \end{aligned} \end{aligned}$$

Due to the ${\alpha ^{\prime }\,}$ pre-factor we are free to take the covariant derivatives in (4.26) to be Chern.

This means that for *SU*(3) manifolds with a smooth ${\alpha ^{\prime }\,}\rightarrow 0$ limit, ${\alpha ^{\prime }\,}P = \mathcal {O}({\alpha ^{\prime }\,}^3)$ and there is no difference between *T* and *H*:

4.30 $$\begin{aligned} H = \textrm{i}({\partial }-{\overline{\partial }})\omega ~. \end{aligned}$$

If we evaluate the trace of of *P* and use that ${\alpha ^{\prime }\,}P = \mathcal {O}({\alpha ^{\prime }\,}^3)$ we find

4.31 $$\begin{aligned} {\alpha ^{\prime }\,}P^\beta {}_{\beta m} \textrm{d}x^m =\frac{ {\alpha ^{\prime }\,}^2}{16} ({\partial }- {\overline{\partial }}) \left( \textrm{tr}\hspace{2.0pt}|F|^2 - \textrm{tr}\hspace{2.0pt}|R^\textrm{H}|^2 \right) = \mathcal {O}({\alpha ^{\prime }\,}^3) ~, \end{aligned}$$

where we again used the gaugino equation in that *F* is traceless. Hence,

$$\begin{aligned} \textrm{tr}\hspace{2.0pt}|F|^2 - \textrm{tr}\hspace{2.0pt}|R^\textrm{H}|^2 = c_0 + \mathcal {O}({\alpha ^{\prime }\,})~, \end{aligned}$$

where $c_0$ is a constant on *X*. In fact, $c_0=0$, since as noticed by [38] and [17] we have

4.32 $$\begin{aligned} \int _X\left( \textrm{tr}\hspace{2.0pt}|F|^2 - \textrm{tr}\hspace{2.0pt}|R^\textrm{H}|^2\right) \, \textrm{d}^{6} x \sqrt{g} = \mathcal {O}({\alpha ^{\prime }\,})~. \end{aligned}$$

Indeed, from (4.27) we have $(dH)_\nu {}^\nu {}_{\mu }{}^\mu = 4\,g^{\mu \bar{\nu }} \partial _\mu \partial _{\bar{\nu }} \Phi + \mathcal {O}({\alpha ^{\prime }\,}^2)$, hence

$$\begin{aligned} \int _X (dH)_\nu {}^\nu {}_{\mu }{}^\mu \, \textrm{d}^{6} x \sqrt{g} = 2 \int _X \nabla ^2 \Phi \, \textrm{d}^{6} x \sqrt{g} + \mathcal {O}({\alpha ^{\prime }\,}^2) = \mathcal {O}({\alpha ^{\prime }\,}^2) \end{aligned}$$

since the Levi-Civita Laplacian $\nabla ^2 \Phi$ differs from the complex Laplacian $2\,g^{\mu \bar{\nu }} \partial _\mu \partial _{\bar{\nu }} \Phi$ by terms of the form $T * \partial \Phi = \mathcal {O}({\alpha ^{\prime }\,}^2)$. Substituting (4.28) gives (4.32).

We have proved

4.33 $$\begin{aligned} H = \textrm{i}({\partial }-{\overline{\partial }})\omega + \mathcal {O}({\alpha ^{\prime }\,}^3)~,\qquad \textrm{tr}\hspace{2.0pt}|F|^2 - \textrm{tr}\hspace{2.0pt}|R^\textrm{H}|^2 = \mathcal {O}({\alpha ^{\prime }\,})~. \end{aligned}$$

#### Double holomorphic direction

We put $m=\mu$ in (4.13), which will correspond to the graviton equation of motion in the direction of two holomorphic coordinates:

4.34 $$\begin{aligned} \begin{aligned}&\left( \textrm{Ric}_{\mu \nu } + 2\nabla _\mu \nabla _\nu \Phi - \frac{1}{4}H_{\mu ab} H_{\nu }{}^{ab} + \frac{{\alpha ^{\prime }\,}}{4}\textrm{tr}\hspace{2.0pt}F_\mu {}^a F_{\nu a} - \frac{{\alpha ^{\prime }\,}}{4}\textrm{tr}\hspace{2.0pt}R^\textrm{H}{}_\mu {}^a R^\textrm{H}{}_{\nu a} \right) \gamma ^\nu \eta \\&+ \frac{1}{2}e^{2\Phi } \nabla ^p (e^{-2\Phi } H_{p\mu \nu } ) \gamma ^\nu \eta = \frac{{\alpha ^{\prime }\,}}{4} \textrm{tr}\hspace{2.0pt}\left( \frac{1}{4} (R^\textrm{H}{\,\wedge \,}R^\textrm{H})_{\mu \nu \alpha {\overline{\beta }}} \gamma ^{\nu \alpha {\overline{\beta }}} \eta - \left( R^\textrm{H}{}_\mu {}^a R^\textrm{H}{}_{\nu a} \right) \gamma ^\nu \eta \right) ~. \end{aligned} \end{aligned}$$

We have used that ${\alpha ^{\prime }\,}P=\mathcal {O}({\alpha ^{\prime }\,}^3)$.

The first line is the graviton equation of motion and the *H* equation of motion. The second line we deal with via

$$\begin{aligned} \begin{aligned} \textrm{tr}\hspace{2.0pt}\left( R^\textrm{H}{}_\mu {}^a R^\textrm{H}{}_{\nu a} \right) \gamma ^\nu \eta - \frac{1}{4}\Big (\textrm{tr}\hspace{2.0pt}(R^\textrm{H}{\,\wedge \,}R^\textrm{H})_{\mu \nu \alpha {\overline{\beta }}} \Big ) \gamma ^{\nu \alpha {\overline{\beta }}} \eta = \textrm{tr}\hspace{2.0pt}(R^\textrm{H}_{\mu \nu } R^\textrm{H}{}_\alpha {}^\alpha )\gamma ^\nu = \mathcal {O}({\alpha ^{\prime }\,}^2) ~. \end{aligned} \end{aligned}$$

We have used that $\textrm{tr}\hspace{2.0pt}R^\textrm{H}{\,\wedge \,}R^\textrm{H}$ has no (4, 0) component, thatas well as $R^\textrm{H}{}^{0,2} = \mathcal {O}({\alpha ^{\prime }\,})$ from (3.10) and $R^\textrm{H}_\alpha {}^\alpha = \mathcal {O}({\alpha ^{\prime }\,})$ from (2.8).

Hence, (4.34) simplifies to

4.35 $$\begin{aligned} \begin{aligned}&\left( \textrm{Ric}_{\mu \nu } + 2\nabla _\mu \nabla _\nu \Phi - \frac{1}{4}H_{\mu ab} H_{\nu }{}^{ab} + \frac{{\alpha ^{\prime }\,}}{4}\textrm{tr}\hspace{2.0pt}F_\mu {}^a F_{\nu a} - \frac{{\alpha ^{\prime }\,}}{4}\textrm{tr}\hspace{2.0pt}R^\textrm{H}{}_\mu {}^a R^\textrm{H}{}_{\nu a} \right) \gamma ^\nu \eta = 0~. \\ \end{aligned} \end{aligned}$$

#### Mixed component

We now study $m={\overline{\mu }}$ in (4.13):

4.36 $$\begin{aligned} \begin{aligned}&\left( \textrm{Ric}_{{\overline{\mu }}\nu } + 2\nabla _{\overline{\mu }}\nabla _\nu \Phi - \frac{1}{4}H_{{\overline{\mu }}ab} H_{\nu }{}^{ab} + \frac{{\alpha ^{\prime }\,}}{4}\textrm{tr}\hspace{2.0pt}F_{\overline{\mu }}{}^a F_{\nu a} - \frac{{\alpha ^{\prime }\,}}{4}\textrm{tr}\hspace{2.0pt}R^\textrm{H}{}_{\overline{\mu }}{}^a R^\textrm{H}{}_{\nu a} \right) \gamma ^\nu \eta \\&\quad + \frac{1}{2}e^{2\Phi } \nabla ^p (e^{-2\Phi } H_{p{\overline{\mu }}\nu } ) \gamma ^\nu \eta = \frac{{\alpha ^{\prime }\,}}{4} \textrm{tr}\hspace{2.0pt}\left( R^\textrm{H}{}_{{\overline{\mu }}\nu } R^\textrm{H}{}_{\alpha }{}^\alpha \right) \gamma ^\nu ~. \end{aligned} \end{aligned}$$

We have used that ${\alpha ^{\prime }\,}P=\mathcal {O}({\alpha ^{\prime }\,}^3)$ and that $\textrm{tr}\hspace{2.0pt}(R^\textrm{H}{\,\wedge \,}R^\textrm{H})_{{\overline{\mu }}\nu \alpha \beta } = \mathcal {O}({\alpha ^{\prime }\,}^2)$ withAs before the first line becomes the graviton equation of motion after using that the *H* equation of motion is satisfied. The second line is related to the trace of $R^\textrm{H}$, followed by additional terms which we now study.

Combining (2.8) and (4.27)

4.37 $$\begin{aligned} g^{\mu {\overline{\nu }}} R^\textrm{H}{}_{\mu {\overline{\nu }}\alpha {\overline{\beta }}} =\! \frac{1}{2}(\textrm{d}H)_\mu {}^\mu {}_{\alpha {\overline{\beta }}} = - 2 \nabla _\alpha \nabla _{\overline{\beta }}\Phi + \mathcal {O}({\alpha ^{\prime }\,}^2)~. \end{aligned}$$

Putting it together, using the *H* equation of motion, we find

4.38 $$\begin{aligned} \begin{aligned}&\left( \textrm{Ric}_{{\overline{\mu }}\nu } + 2\nabla _{\overline{\mu }}\nabla _\nu \Phi - \frac{1}{4}H_{{\overline{\mu }}ab} H_{\nu }{}^{ab} + \frac{{\alpha ^{\prime }\,}}{4}\textrm{tr}\hspace{2.0pt}F_{\overline{\mu }}{}^a F_{\nu a} - \frac{{\alpha ^{\prime }\,}}{4}\textrm{tr}\hspace{2.0pt}R^\textrm{H}{}_{\overline{\mu }}{}^a R^\textrm{H}{}_{\nu a} \right) \gamma ^\nu \eta \\&\quad = - {\alpha ^{\prime }\,}\left( R^\textrm{H}{}_{\nu {\overline{\mu }}}{}^{\alpha {\overline{\beta }}} \left( \nabla _\alpha \nabla _{\overline{\beta }}\Phi \right) \right) \gamma ^\nu \eta ~. \end{aligned} \end{aligned}$$

### Summary

If we assume there is a smooth ${\alpha ^{\prime }\,}\rightarrow 0$ limit, we have shown in earlier sections that supersymmetry implies $H=\mathcal {O}({\alpha ^{\prime }\,})$, $\nabla \Phi = \mathcal {O}({\alpha ^{\prime }\,})$; that the *H* and *F* equation of motion holds to ${\alpha ^{\prime }\,}^2$ and if the Bianchi identity holds then we have shown an integrability condition

4.39 $$\begin{aligned} \begin{aligned}&\left( \textrm{Ric}_{\mu \nu } + 2\nabla _\mu \nabla _\nu \Phi - \frac{1}{4}H_{\mu ab} H_{\nu }{}^{ab} + \frac{{\alpha ^{\prime }\,}}{4}\textrm{tr}\hspace{2.0pt}F_\mu {}^a F_{\nu a} - \frac{{\alpha ^{\prime }\,}}{4}\textrm{tr}\hspace{2.0pt}R^\textrm{H}{}_\mu {}^a R^\textrm{H}{}_{\nu a} \right) \gamma ^\nu \eta = 0~, \\&\left( \textrm{Ric}_{{\overline{\mu }}\nu } + 2\nabla _{\overline{\mu }}\nabla _\nu \Phi - \frac{1}{4}H_{{\overline{\mu }}ab} H_{\nu }{}^{ab} + \frac{{\alpha ^{\prime }\,}}{4}\textrm{tr}\hspace{2.0pt}F_{\overline{\mu }}{}^a F_{\nu a} - \frac{{\alpha ^{\prime }\,}}{4}\textrm{tr}\hspace{2.0pt}R^\textrm{H}{}_{\overline{\mu }}{}^a R^\textrm{H}{}_{\nu a} \right) \gamma ^\nu \eta \\&\qquad \qquad \qquad =\! - {\alpha ^{\prime }\,}\left( R^\textrm{H}{}_{\nu {\overline{\mu }}}{}^{\alpha {\overline{\beta }}} \left( \nabla _\alpha \nabla _{\overline{\beta }}\Phi \right) \right) \gamma ^\nu \eta ~. \end{aligned} \end{aligned}$$

These are the graviton equations of motion sourced by a term proportional to the hessian of the dilaton.

We now use that on an *SU*(3) structure manifold the dilaton’s Hessian is pure gauge–meaning it can be set to zero by a diffeomorphism–up to order $\alpha '^2$ [17, 38]. We explore this gauge freedom in detail in §5.7 and we call it constant dilaton gauge. Hence, (4.39) is the graviton equation of motion in constant dilaton gauge.

#### Remarks

First, the dilaton equations of motion do not appear in this analysis. Second, in contrast to e.g. [39–43], no instanton condition is required on *R*.

A corollary of (4.37) is that the trace of $R^\textrm{H}$ is pure gauge to first order in ${\alpha ^{\prime }\,}$ and vanishes in the constant dilaton gauge. However, $R^{\textrm{H}\,0,2}$ does not vanish, see (3.10), even in this gauge. $R^\textrm{H}$ is not an instanton as required by supersymmetry, see (2.8).

## Equations of Motion from Hermitian Geometry

### Statement of results

In Sect. 2, we started from constraints on a spinor $\eta$ on a compact 6-manifold *X* obtained by setting fermionic supersymmetry variations to zero, and from these equations on spinors we found a complex structure, a holomorphic volume form, and constraints for the metric tensor intertwining these structures at ${\alpha ^{\prime }\,}^2$.

This section tells a self-contained mathematical story based on our findings in earlier sections. The setup is as follows:

1. Let *X* be a compact manifold of dimension 6. Let $(g_{\alpha ^{\prime }\,}, J_{\alpha ^{\prime }\,}, \Omega _{\alpha ^{\prime }\,}, F_{\alpha ^{\prime }\,})$ be a family of tensors on *X* smoothly varying with a parameter ${\alpha ^{\prime }\,}\in [0,\epsilon )$ for $\epsilon >0$ fixed, where $g_{\alpha ^{\prime }\,}$ is a metric tensor, $J_{\alpha ^{\prime }\,}$ is a complex structure, $\Omega _{\alpha ^{\prime }\,}$ is a holomorphic volume form, and $F_{\alpha ^{\prime }\,}$ is the curvature of a connection on a vector bundle $E \rightarrow X$. For example,
  $$ g_{\alpha ^{\prime }\,}= g^{(0)} + {\alpha ^{\prime }\,}g^{(1)} + {\alpha ^{\prime }\,}^2\,g^{(2)} + \dots ~, $$
  and $g^{(0)}$ is a metric tensor.
2. Suppose $(g_{\alpha ^{\prime }\,}, J_{\alpha ^{\prime }\,}, \Omega _{\alpha ^{\prime }\,}, F_{\alpha ^{\prime }\,})$ solves the following equations of complex geometry:
  5.1 $$\begin{aligned} d (\left\| \Omega \right\| _g \omega ^2)&= 0 \end{aligned}$$
  5.2 $$\begin{aligned} F^{0,2} =F^{2,0}&= 0~, \end{aligned}$$
  5.3 $$\begin{aligned} F \wedge \omega ^2&= 0 ~, \end{aligned}$$
  5.4 Here $\omega =g (J \cdot , \cdot )$ as usual. Here and in what follows, we will often drop the subscript ${\alpha ^{\prime }\,}$ on tensors and simply write $g = g_{{\alpha ^{\prime }\,}}$, $\Omega =\Omega _{\alpha ^{\prime }\,}$, etc.
3. Define the 3-form $H_{\alpha ^{\prime }\,}$ and scalar function $\Phi _{\alpha ^{\prime }\,}$ by:
  5.5 $$\begin{aligned} H&= \textrm{i}(\partial -\bar{\partial })\omega ~, \end{aligned}$$
  5.6 $$\begin{aligned} \Phi&=\! - \frac{1}{2}\log \left\| \Omega \right\| _g ~. \end{aligned}$$
  In this section, we simply take equations (5.5) and (5.6) as mathematical definitions. We note that in previous sections, we showed that the physical fields *g*, *H*, and $\Phi$ of string theory, which receive ${\alpha ^{\prime }\,}$ corrections to all orders, are consistent with the truncated equations given here to order ${\alpha ^{\prime }\,}^2$ in the ${\alpha ^{\prime }\,}$-expansion. In particular, equations (5.4) and (5.5) are consistent with the Bianchi identity $dH = \frac{{\alpha ^{\prime }\,}}{4} ( \textrm{tr}\hspace{2.0pt}FF - \textrm{tr}\hspace{2.0pt}R^\textrm{H}R^\textrm{H})$ up to $\mathcal {O}({\alpha ^{\prime }\,}^3)$ corrections; see Sect. 3.3.

We investigate the question of whether these equations of complex geometry imply the equations of motion at order ${\alpha ^{\prime }\,}^2$, which we reprint here for ease of reference:5.7These equations, as listed in that order, correspond to varying the action with respect to $g_{mn}$, $B_{mn}$, *A* and $\Phi$. Here $R^\textrm{H}$ is defined by $\Gamma _m^\textrm{H}= \Gamma ^\textrm{LC}_m + \frac{1}{2}H_m$ and  acts as $\Gamma _m^- = \Gamma ^\textrm{LC}_m - \frac{1}{2}H_m$ on tangent bundle indices. In the current section, we simply write $\nabla$ instead of $\nabla ^{\textrm{LC}}$ for the Levi-Civita connection for ease of notation.

Section 4 analyzed the relation between the supersymmetry constraints on spinors and the equations of motion. The current section gives a second perspective by working only at the level of complex geometry and making no mention of spinors. The precise statement of the result in this section is:

#### Proposition 3

Under the setup assumptions denoted above by 1, 2, and 3, there exists a 1-parameter family of diffeomorphisms $\varphi _{\alpha ^{\prime }\,}$ with $\varphi _0=id$ such that $(\check{g}_{\alpha ^{\prime }\,}, \check{\Phi }_{\alpha ^{\prime }\,}, \check{H}_{\alpha ^{\prime }\,}, \check{F}_{\alpha ^{\prime }\,})$ solve the equations of motion (5.7) where check denotes the pullback by $\varphi _{\alpha ^{\prime }\,}$. For example, $\check{g}_{\alpha ^{\prime }\,}= \varphi _{\alpha ^{\prime }\,}^* g_{\alpha ^{\prime }\,}$.

We make three remarks on this Proposition:

- If we wish to be consistent with the equations of motion at first order in ${\alpha ^{\prime }\,}$, which amounts to replacing $\mathcal {O}({\alpha ^{\prime }\,}^3)$ with $\mathcal {O}({\alpha ^{\prime }\,}^2)$ on the right-hand side of (5.7), then it is not needed to pullback by diffeomorphisms $\varphi _{\alpha ^{\prime }\,}$. In that case, the equations of motion are a direct consequence of assumptions 1, 2, and 3. It is at order ${\alpha ^{\prime }\,}^2$ that there are extra terms which can be removed by gauge fixing along the flow of ${\alpha ^{\prime }\,}$ via $\varphi _{\alpha ^{\prime }\,}$.
- Some other studies of heterotic supersymmetry and the equations of motion add the extra hypothesis that *R* is an instanton e.g. [39–43], but this condition does not play a role in the proof of Proposition 3, and is not compatible with supersymmetry at ${\alpha ^{\prime }\,}^2$ as explained by Bergshoeff–de Roo [1]. This is because the condition
  $$\begin{aligned} g^{\mu \bar{\nu }} R_{\mu {\overline{\nu }}}+ \mathcal {O}({\alpha ^{\prime }\,}) =0~, \qquad R_{{\overline{\mu }}{\overline{\nu }}} + \mathcal {O}({\alpha ^{\prime }\,}) = 0~, \end{aligned}$$
  which automatically holds for $R=R^\textrm{H}$ or $R=R^{\textrm{CH}}$ under setup assumptions 1, 2 and 3, and which appears when deriving the equations of motion from supersymmetry constraints at first order in $\alpha '$ with a general curvature tensor *R* in the Bianchi identity, is refined to
  $$\begin{aligned} \left( R^\textrm{H}_{\mu {\overline{\nu }}}{}^{\alpha {\overline{\beta }}}- \frac{1}{2} (\textrm{d}H)_{\mu {\overline{\nu }}}{}^{\alpha {\overline{\beta }}}\right) g^{\mu {\overline{\nu }}} + \mathcal {O}({\alpha ^{\prime }\,}^2) =0~, \qquad R^\textrm{H}_{{\overline{\mu }}{\overline{\nu }}}- \frac{1}{2} (\textrm{d}H)_{{\overline{\mu }}{\overline{\nu }}} + \mathcal {O}({\alpha ^{\prime }\,}^2) = 0~ , \end{aligned}$$
  in the BdR algebra at second order in $\alpha '$ (see Sect. 2.1), and this refinement also automatically holds under setup assumptions 1, 2 and 3 (C.12); therefore, no further condition needed in the assumptions in Proposition 3.
- Sequences solving assumptions 1 and 2 have been constructed by various methods; see [18, 44–46].

Lastly, a comment about notation. In this section, we will distinguish equality up to error terms of order $\mathcal {O}({\alpha ^{\prime }\,}^3)$ and exact equality. We use the notation

$$\begin{aligned} A \overset{\ {\alpha ^{\prime }\,}^2}{=}\ B \quad \Leftrightarrow \quad A = B + \mathcal {O}({\alpha ^{\prime }\,}^3)~. \end{aligned}$$

This distinction between $=$ and $\overset{\ {\alpha ^{\prime }\,}^2}{=}$ here is only included as a mathematical exercise; in all other sections of the paper this was understood by context and suppressed for ease of notation.

### Preliminaries

We proved in Sect. 3.1 that the setup denoted above by 1, 2 and 3 implies

$$\begin{aligned} H = \mathcal {O}({\alpha ^{\prime }\,}), \quad T = \mathcal {O}({\alpha ^{\prime }\,}), \quad \partial \Phi = \mathcal {O}({\alpha ^{\prime }\,})~. \end{aligned}$$

We will use this frequently in the following subsections. The conformally balanced equation (5.1) and the definition of $\Phi$ (5.6) give via (C.3) the identity

5.8 $$\begin{aligned} \textrm{d}( e^{-2 \Phi } \omega ^2) =0~, \quad H_\mu {}^\mu {}_\lambda =\! - 2 \partial _\lambda \Phi ~, \quad H_\mu {}^\mu {}_{\bar{\lambda }} = 2 \partial _{\bar{\lambda }} \Phi ~. \end{aligned}$$

In the remainder of this subsection we prove various identities for future reference.

- The first identity we prove is:
  5.9 $$\begin{aligned}&\ \frac{{\alpha ^{\prime }\,}}{8} (\textrm{tr}\hspace{2.0pt}F \wedge F - \textrm{tr}\hspace{2.0pt}R^\textrm{CH}\wedge R^\textrm{CH})_\mu {}^\mu {}_{\bar{\beta } \alpha } \nonumber \\&\overset{\ {\alpha ^{\prime }\,}^2}{=}\ - \frac{{\alpha ^{\prime }\,}}{4} \textrm{tr}\hspace{2.0pt}\bigg [ F_{\alpha \bar{\mu }} F_{\bar{\beta }}{}^{\bar{\mu }} -R^\textrm{H}{}_{\alpha \bar{\mu }} R^\textrm{H}{}_{\bar{\beta }}{}^{\bar{\mu }} \bigg ] - \frac{{\alpha ^{\prime }\,}}{4} (\Lambda _\omega \partial \bar{\partial } \omega )^m{}_n R^\textrm{CH}{}_{\alpha \bar{\beta }}{}^n{}_m~. \end{aligned}$$
  To show (5.9), we start with
  $$\begin{aligned} (\textrm{tr}\hspace{2.0pt}F \wedge F)_{\mu \bar{\nu } \alpha \bar{\beta }} = 2 \textrm{tr}\hspace{2.0pt}F_{\mu \bar{\nu }} F_{\alpha \bar{\beta }} - 2 \textrm{tr}\hspace{2.0pt}F_{\mu \bar{\beta }} F_{\alpha \bar{\nu }}~, \end{aligned}$$
  and so since $g^{\mu \bar{\nu }} F_{\mu \bar{\nu }}=0$ then
  5.10 $$\begin{aligned} (\textrm{tr}\hspace{2.0pt}F \wedge F)_{\mu }{}^\mu {}_{\alpha \bar{\beta }} =\! - 2 \textrm{tr}\hspace{2.0pt}F_{\mu \bar{\beta }} F_{\alpha }{}^\mu ~. \end{aligned}$$
  Similarly,
  $$\begin{aligned} {\alpha ^{\prime }\,}(\textrm{tr}\hspace{2.0pt}R^\textrm{CH}\wedge R^\textrm{CH})_{\mu }{}^\mu {}_{\alpha \bar{\beta }} = 2 {\alpha ^{\prime }\,}g^{\mu \bar{\nu }} \textrm{tr}\hspace{2.0pt}R^\textrm{CH}{}_{\mu \bar{\nu }} R^\textrm{CH}{}_{\alpha \bar{\beta }} - 2 {\alpha ^{\prime }\,}g^{\mu \bar{\nu }} \textrm{tr}\hspace{2.0pt}R^\textrm{CH}{}_{\mu \bar{\beta }} R^\textrm{CH}{}_{\alpha \bar{\nu }}~. \end{aligned}$$
  Up to errors of order $\mathcal {O}({\alpha ^{\prime }\,}^3)$, we can convert the second term from Chern to Hull.
  $$\begin{aligned} - 2 {\alpha ^{\prime }\,}g^{\mu \bar{\nu }} \textrm{tr}\hspace{2.0pt}R^\textrm{CH}{}_{\mu \bar{\beta }} R^\textrm{CH}{}_{\alpha \bar{\nu }} \overset{\ {\alpha ^{\prime }\,}^2}{=}\ - 2 {\alpha ^{\prime }\,}\textrm{tr}\hspace{2.0pt}R^\textrm{H}{}_{\mu \bar{\beta }} R^\textrm{H}{}_{\alpha }{}^\mu ~. \end{aligned}$$
  Indeed, the explicit formulas in (C.6) show that $R^\textrm{H}{}_{\alpha \bar{\beta }}{}^{\bar{\gamma }}{}_{\lambda } = \mathcal {O}({\alpha ^{\prime }\,})$ and
  5.11 $$\begin{aligned} R^{\textrm{H}}{}_{\alpha \bar{\beta }}{}^\lambda {}_\gamma = R^\textrm{Ch}{}_{\alpha \bar{\beta }}{}^\lambda {}_\gamma + T_\alpha {}^\lambda {}_{\bar{\sigma }} T_{\bar{\beta }}{}^{\bar{\sigma }}{}_\gamma ~, \quad T = \mathcal {O}({\alpha ^{\prime }\,})~. \end{aligned}$$
  Thus
  5.12 $$\begin{aligned}&\ \frac{{\alpha ^{\prime }\,}}{8} (\textrm{tr}\hspace{2.0pt}F \wedge F - \textrm{tr}\hspace{2.0pt}R^\textrm{CH}\wedge R^\textrm{CH})_\mu {}^\mu {}_{\bar{\beta } \alpha } \nonumber \\&\overset{\ {\alpha ^{\prime }\,}^2}{=}\ \!- \frac{{\alpha ^{\prime }\,}}{4} \textrm{tr}\hspace{2.0pt}\bigg [ F_{\alpha \bar{\mu }} F_{\bar{\beta }}{}^{\bar{\mu }} -R^\textrm{H}{}_{\alpha \bar{\mu }} R^\textrm{H}{}_{\bar{\beta }}{}^{\bar{\mu }} \bigg ] - \frac{{\alpha ^{\prime }\,}}{4} g^{\mu \bar{\nu }} \textrm{tr}\hspace{2.0pt}R^\textrm{CH}{}_{\mu \bar{\nu }} R^\textrm{CH}{}_{\alpha \bar{\beta }} ~. \end{aligned}$$
  The term $g^{\mu \bar{\nu }} R^\textrm{CH}_{\mu \bar{\nu }}$ was computed in the appendix (C.11), and at order ${\alpha ^{\prime }\,}^2$ we have
  5.13 $$\begin{aligned} - {\alpha ^{\prime }\,}\textrm{tr}\hspace{2.0pt}g^{\mu \bar{\nu }} R^\textrm{CH}{}_{\mu \bar{\nu }}{}^\alpha {}_\beta \overset{\ {\alpha ^{\prime }\,}^2}{=}\ {\alpha ^{\prime }\,}(i \partial \bar{\partial } \omega )_\mu {}^{\mu \alpha }{}_\beta ~. \end{aligned}$$
  This proves (5.9).
- Equation (5.9) involves $\Lambda _\omega \partial \bar{\partial } \omega$. We remark that
  5.14 $$\begin{aligned} \Lambda _\omega \textrm{d}\textrm{d}^c \omega =\! - \Lambda _\omega \textrm{d}^c \textrm{d}\omega =\! - \textrm{d}^c \Lambda _\omega \textrm{d}\omega + \mathcal {O}(d \omega )^2 ~, \end{aligned}$$
  using the Käher identity $[\Lambda ,\textrm{d}^c] = \mathcal {O}(\textrm{d}\omega )$. Substituting $\Lambda _\omega \textrm{d}\omega = 2 \textrm{d}\Phi$ (5.8) and $T = \mathcal {O}({\alpha ^{\prime }\,})$ then leads to
  5.15 $$\begin{aligned} \Lambda _\omega (\textrm{i}\partial \bar{\partial } \omega ) =2 \textrm{i}\partial \bar{\partial } \Phi + \mathcal {O}({\alpha ^{\prime }\,}^2)~. \end{aligned}$$

### Yang–Mills equation

It is well-known (see e.g. [47]) that

$$\begin{aligned} d (\left\| \Omega \right\| _g \omega ^2) =0,~ \quad g^{\mu \bar{\nu }} F_{\mu \bar{\nu }}= 0, \quad F^{0,2} = F^{2,0} = 0,~ \end{aligned}$$

implies5.16We outlined the derivation earlier in Sect. 4.2.1. Here we simply note that the Hermitian-Yang-Mills equation $g^{\mu \bar{\nu }} F_{\mu \bar{\nu }} = 0$ does not imply the usual Yang-Mills equation $\nabla ^m F_{mn} = 0$ when *g* is non-Kähler. Rather, (5.16) is the correct adaptation of the Yang-Mills equation, as this is the equation derived from varying the gauge field in the action (1.1).

### Divergence of the 3-form

In this subsection, we derive

5.17 $$\begin{aligned} \nabla ^m(\textrm{e}^{-2\Phi } H_{mij}) = 0~. \end{aligned}$$

We take the approach of [40]. The starting point is the following observation due to [48]: let $\omega$ be a conformally balanced metric on a complex manifold *X* of dimension 3 satisfying $d (e^{-2 \Phi } \omega ^2) = 0$ for some scalar function $\Phi$. Then

5.18 $$\begin{aligned} \star T =\! - e^{2 \Phi } \textrm{d}(e^{-2 \Phi } \omega )~, \quad \textrm{d}\star (e^{-2 \Phi } T) = 0~, \quad T = i (\partial - \bar{\partial })\omega ~. \end{aligned}$$

In our current setup, we solve $d (e^{-2 \Phi } \omega ^2) = 0$ by (5.8). To show (5.18), we can first use the primitive decomposition to derive the general formula

5.19 $$\begin{aligned} \star \chi = J \chi - J(\Lambda _\omega \chi ) \wedge \omega ~, \quad \chi \in \Omega ^3(X)~. \end{aligned}$$

Here *J* acts on differential forms by dual pairings, so that for example in holomorphic coordinates  and . Equation (5.18) then follows from letting $\chi = T$ in (5.19) and using $i \Lambda _\omega T = -2(\partial -\bar{\partial }) \Phi$ (C.3). From here, we can derive the *H*-equation of motion (5.17) by setting $H = T$.

### Einstein equation

In this subsection, we show that if $\Phi = \textrm{const} + \mathcal {O}({\alpha ^{\prime }\,}^2)$, then

5.20 $$\begin{aligned} \textrm{Ric}_{mn}+ 2 \nabla _m \nabla _n \Phi - \frac{1}{4} H_{mpq} H_n{}^{pq} +\frac{{\alpha ^{\prime }\,}}{4} \textrm{tr}\hspace{2.0pt}F_{mp} F_n{}^p - \frac{{\alpha ^{\prime }\,}}{4} \textrm{tr}\hspace{2.0pt}R^\textrm{H}{}_{mp} R^\textrm{H}{}_n{}^p \overset{\ {\alpha ^{\prime }\,}^2}{=}\ 0~. \end{aligned}$$

We will pullback by a certain family of diffeomorphisms along the flow of ${\alpha ^{\prime }\,}$ to arrange that $\Phi$ is a constant plus quadratic terms in ${\alpha ^{\prime }\,}$. This will be done later in Sect. 5.7. For now, we conclude that if $\Phi = \textrm{const} + \mathcal {O}({\alpha ^{\prime }\,}^2)$, then the Einstein equation is satisfied to order ${\alpha ^{\prime }\,}^2$. In the detailed calculation below, we will see the extra terms that arise if $\Phi$ is not constant to this order.

#### Double holomorphic directions

We start by examining (5.20) when *m*, *n* are both holomorphic indices. The first step is to note the general formula for the Ricci curvature of a hermitian metric $(X,\omega )$ along two holomorphic indices:

$$\begin{aligned} \textrm{Ric}_{\alpha \beta } = \frac{1}{2}(\nabla _\alpha ^{\textrm{CH}} T_\mu {}^\mu {}_\beta + \nabla _\beta ^{\textrm{CH}} T_\mu {}^\mu {}_\alpha ) +\frac{1}{2}T_{\alpha \mu \bar{\nu }} T^{\mu \bar{\nu }}{}_\beta ~, \quad T = \textrm{i}(\partial -\bar{\partial })\omega ~. \end{aligned}$$

We now substitute (5.8).

$$\begin{aligned} \textrm{Ric}_{\alpha \beta } =\! - \nabla _\alpha ^{\textrm{CH}} \partial _\beta \Phi - \nabla ^{\textrm{CH}}_\beta \partial _\alpha \Phi + \frac{1}{2}H_{\alpha \mu \bar{\nu }} H^{\mu \bar{\nu }}{}_\beta ~. \end{aligned}$$

Converting the Chern connection $\nabla ^\textrm{CH}$ to the Levi-Civita connection $\nabla$ gives:

5.21 $$\begin{aligned} \textrm{Ric}_{\alpha \beta } + 2 \nabla _\alpha \nabla _\beta \Phi -\frac{1}{4} H_{\alpha pq} H_\beta {}^{pq} = 0~. \end{aligned}$$

If we compare with (5.20), there is a term like

$$\begin{aligned} \frac{{\alpha ^{\prime }\,}}{4} \textrm{tr}\hspace{2.0pt}F_{\alpha m} F_\beta {}^m - \frac{{\alpha ^{\prime }\,}}{4} \textrm{tr}\hspace{2.0pt}R^\textrm{H}{}_{\alpha m} R^\textrm{H}{}_\beta {}^m =\! - \frac{{\alpha ^{\prime }\,}}{4} g^{\mu \bar{\nu }} \textrm{tr}\hspace{2.0pt}R^\textrm{H}{}_{\alpha \mu } R^\textrm{H}{}_{\beta \bar{\nu }} - \frac{{\alpha ^{\prime }\,}}{4} g^{\mu \bar{\nu }} \textrm{tr}\hspace{2.0pt}R^\textrm{H}{}_{\alpha \bar{\nu }} R^\textrm{H}{}_{\beta \mu }~. \end{aligned}$$

This is order $\mathcal {O}({\alpha ^{\prime }\,}^3)$. Indeed, we can compute the curvature ${\alpha ^{\prime }\,}R^\textrm{H}$ using the explicit formulas in (C.6). The only non-zero component of $R^{\textrm{H}}{}_{\alpha \beta }{}^m{}_n$ is $R^{\textrm{H}}{}_{\alpha \beta }{}^\mu {}_{\bar{\nu }}$. Hence

5.22 $$\begin{aligned} - \frac{{\alpha ^{\prime }\,}}{4} \textrm{tr}\hspace{2.0pt}R^\textrm{H}{}_{\alpha m} R^\textrm{H}{}_\beta {}^m \overset{\ {\alpha ^{\prime }\,}^2}{=}\! - \frac{{\alpha ^{\prime }\,}}{4} g^{\mu \bar{\nu }} \textrm{tr}\hspace{2.0pt}R^\textrm{H}{}_{\alpha \mu }{}^\sigma {}_{\bar{\delta }} R^\textrm{H}{}_{\beta \bar{\nu }}{}^{\bar{\delta }}{}_\sigma - \frac{{\alpha ^{\prime }\,}}{4} g^{\mu \bar{\nu }} \textrm{tr}\hspace{2.0pt}R^\textrm{H}{}_{\alpha \bar{\nu }}{}^{\bar{\delta }}{}_\sigma R^\textrm{H}{}_{\beta \mu }{}^\sigma {}_{\bar{\delta }}~. \end{aligned}$$

From here we can use

$$\begin{aligned} R^{\textrm{H}}{}_{\mu \nu }{}^\alpha {}_{\bar{\beta }} =\! - (\textrm{i}\partial \bar{\partial } \omega )_{\mu \nu }{}^\alpha {}_{\bar{\beta }}~, \quad R^{\textrm{H}}{}_{\beta \bar{\nu }}{}^{\bar{\delta }}{}_\sigma = \nabla ^{\textrm{CH}}_\beta H_{\bar{\nu }}{}^{\bar{\delta }}{}_\sigma ~, \end{aligned}$$

to reduce (5.22) to $\mathcal {O}({\alpha ^{\prime }\,}^3)$. Thus

$$\begin{aligned} \textrm{Ric}_{\alpha \beta }+ 2 \nabla _\alpha \nabla _\beta \Phi - \frac{1}{4} H_{\alpha pq} H_\beta {}^{pq} +\frac{{\alpha ^{\prime }\,}}{4} \textrm{tr}\hspace{2.0pt}F_{\alpha m} F_\beta {}^m - \frac{{\alpha ^{\prime }\,}}{4} \textrm{tr}\hspace{2.0pt}R^\textrm{H}{}_{\alpha m} R^\textrm{H}{}_\beta {}^m \overset{\ {\alpha ^{\prime }\,}^2}{=}\ 0~. \end{aligned}$$

Therefore the Einstein equation holds on double holomorphic indices.

#### Mixed directions

Next, we return to (5.20) and consider the case when *m*, *n* are mixed barred/unbarred indices. As before, the starting point is the general formula for the Ricci curvature in complex geometry, which expresses the Ricci curvature in terms of the first Chern class representative:

5.23 $$\begin{aligned} \textrm{Ric}_{\alpha \bar{\beta }}&= R^{\textrm{Ch}}{}_{\alpha \bar{\beta }}{}^\mu {}_\mu + \frac{1}{2} (dT)_\mu {}^\mu {}_{\bar{\beta } \alpha } + \frac{1}{2} \nabla _\alpha ^{\textrm{Ch}} T_\mu {}^\mu {}_{\bar{\beta }} - \frac{1}{2} \nabla _{\bar{\beta }}^{\textrm{Ch}} T_\mu {}^\mu {}_\alpha \nonumber \\&+ \frac{1}{2} T_{\alpha \mu \bar{\nu }} T^{\mu \bar{\nu }}{}_{\bar{\beta }} + \frac{1}{4} T_{\alpha \bar{\mu } \bar{\nu }} T^{\bar{\mu } \bar{\nu }}{}_{\bar{\beta }} - \frac{1}{2} T_\alpha {}^\mu {}_\beta T_\nu {}^\nu {}_\mu - \frac{1}{2} T_\alpha {}^{\bar{\mu }}{}_\beta T_\nu {}^\nu {}_{\bar{\mu }} ~. \end{aligned}$$

This holds on any hermitian manifold $(X,\omega )$ and can be calculated directly from the conventions stated in the appendix. We can then rewrite (5.23) in our particular setup, where we substitute $R^{\textrm{Ch}}{}_{\alpha \bar{\beta }}{}^\mu {}_\mu = \partial _\alpha \partial _{\bar{\beta }} \log |\Omega |^2_\omega$, $\partial \log \left\| \Omega \right\| _g = -2 \partial \Phi$, $T=H$ and (5.8).

5.24 $$\begin{aligned} \begin{aligned} \textrm{Ric}_{\alpha \bar{\beta }}&=\! -2 \partial _\alpha \partial _{\bar{\beta }} \Phi + \frac{1}{2} (d H)_\mu {}^\mu {}_{\bar{\beta } \alpha } \\&+ \frac{1}{2} H_{\alpha \mu \bar{\nu }} H^{\mu \bar{\nu }}{}_{\bar{\beta }} + \frac{1}{4} H_{\alpha \bar{\mu } \bar{\nu }} H^{\bar{\mu } \bar{\nu }}{}_{\bar{\beta }} + H_\alpha {}^\mu {}_\beta \Phi _\mu - H_\alpha {}^{\bar{\mu }}{}_\beta \Phi _{\bar{\mu }}~ . \end{aligned} \end{aligned}$$

The Hessian of the Levi-Civita connection in holomorphic coordinates is

$$\begin{aligned} \nabla _\alpha \nabla _{\bar{\beta }} \Phi = \partial _\alpha \partial _{\bar{\beta }} \Phi - \frac{1}{2} H_\alpha {}^\mu {}_{\bar{\beta }} \Phi _\mu + \frac{1}{2} H_\alpha {}^{\bar{\mu }}{}_{\bar{\beta }} \Phi _{\bar{\mu }}~, \end{aligned}$$

and so using equation (5.4) for $i \partial \bar{\partial } \omega$ gives

5.25 $$\begin{aligned} \textrm{Ric}_{\alpha \bar{\beta }} + 2 \nabla _\alpha \nabla _{\bar{\beta }} \Phi -\frac{1}{4} H_{\alpha mn} H_{\bar{\beta }}{}^{mn} - \frac{{\alpha ^{\prime }\,}}{8} (\textrm{tr}\hspace{2.0pt}F \wedge F - \textrm{tr}\hspace{2.0pt}R^\textrm{CH}\wedge R^\textrm{CH})_\mu {}^\mu {}_{\bar{\beta } \alpha } = 0~. \end{aligned}$$

We can now apply (5.9) to the last term. Substituting (5.9) into (5.25) gives

5.26 $$\begin{aligned}&\ \textrm{Ric}_{\alpha \bar{\beta }} + 2 \nabla _\alpha \nabla _{\bar{\beta }} \Phi -\frac{1}{4} H_{\alpha mn} H_{\bar{\beta }}{}^{mn} + \frac{{\alpha ^{\prime }\,}}{4} \bigg [ F_{\alpha \bar{\mu }} F_{\bar{\beta }}{}^{\bar{\mu }} -R^\textrm{H}{}_{\alpha \bar{\mu }} R^\textrm{H}{}_{\bar{\beta }}{}^{\bar{\mu }} \bigg ] \nonumber \\&\overset{\ {\alpha ^{\prime }\,}^2}{=}\! - \frac{{\alpha ^{\prime }\,}}{4} (\Lambda _\omega \partial \bar{\partial } \omega )^\lambda {}_\gamma R^\textrm{CH}{}_{\alpha \bar{\beta }}{}^\gamma {}_\lambda ~ . \end{aligned}$$

There is a troublesome term on the right-hand side of (5.26). At linear order in ${\alpha ^{\prime }\,}$, this term is negligible and the equations of motion are satisfied.

The subtlety arises at order ${\alpha ^{\prime }\,}^2$. To remove the troublesome term, we will pullback by a certain family of diffeomorphisms along the flow of ${\alpha ^{\prime }\,}$ to arrange that $\Phi$ is a constant plus quadratic terms in ${\alpha ^{\prime }\,}$. This will be done later in Sect. 5.7. For now, we conclude that if $\Phi = \textrm{const} + \mathcal {O}({\alpha ^{\prime }\,}^2)$ then (5.15) implies

5.27 $$\begin{aligned} {\alpha ^{\prime }\,}\Lambda \partial \bar{\partial } \omega \overset{\ {\alpha ^{\prime }\,}^2}{=}\ 0~. \end{aligned}$$

With this, equation (5.26) becomes the Einstein equation to order ${\alpha ^{\prime }\,}^2$.

### Dilaton equation

Finally, we must derive the dilaton equation

5.28 $$\begin{aligned} R - 4(\nabla \Phi )^2 + 4 \nabla ^2 \Phi - \frac{1}{2} |H|^2 + \frac{{\alpha ^{\prime }\,}}{4} \textrm{tr}\hspace{2.0pt}|F|^2 - \frac{{\alpha ^{\prime }\,}}{4} \textrm{tr}\hspace{2.0pt}|R^\textrm{H}|^2 \overset{\ {\alpha ^{\prime }\,}^2}{=}\ 0~. \end{aligned}$$

Taking the trace of the Einstein equation (5.20) does not immediately produce the dilaton equation. So instead we start by deriving an identity for $\nabla ^2 \Phi$ by using special identities from conformally balanced metrics on complex manifolds.

We start with the definition of the Laplacian in holomorphic coordinates:

$$\begin{aligned} \nabla ^2 \log \left\| \Omega \right\| _g = g^{\alpha \bar{\beta }} \partial _\alpha \partial _{\bar{\beta }} \log |\Omega |^2_\omega - T_\alpha {}^{\mu \alpha } \partial _\mu \log \left\| \Omega \right\| _g + T_\alpha {}^{\bar{\mu } \alpha } \partial _{\bar{\mu }} \log \left\| \Omega \right\| _g~. \end{aligned}$$

On a general conformally balanced geometry $(X,\omega ,\Omega )$ satisfying $d (\left\| \Omega \right\| _g \omega ^2)=0$, we can apply (C.9) and (C.11), hence there holds

$$\begin{aligned} \nabla ^2 \log \left\| \Omega \right\| _g =\! - (\textrm{i}\partial \bar{\partial } \omega )_\alpha {}^{\alpha \mu }{}_\mu + T_{\alpha \mu \bar{\nu }} T^{\mu \bar{\nu } \alpha }- T_\alpha {}^{\mu \alpha } \partial _\mu \log \left\| \Omega \right\| _g + T_\alpha {}^{\bar{\mu } \alpha } \partial _{\bar{\mu }} \log \left\| \Omega \right\| _g~. \end{aligned}$$

In our setup, the $\log \left\| \Omega \right\| _g$ is related to the dilaton via $\partial _i \log \left\| \Omega \right\| _g = - 2 \partial _i \Phi$, $H=T$, and our conventions are $|H|^2=\frac{1}{6} H_{mnp} H^{mnp}$. Thus

$$\begin{aligned} \nabla ^2 \Phi = \frac{1}{2} (i \partial \bar{\partial } \omega )_\alpha {}^{\alpha \mu }{}_\mu - \frac{1}{2}|H|^2 + H_\alpha {}^{\mu \alpha } \partial _\mu \Phi - H_\alpha {}^{\bar{\mu } \alpha } \partial _{\bar{\mu }} \Phi ~. \end{aligned}$$

Since $H_\mu {}^\mu {}_\lambda = - 2 \partial _\lambda \Phi$, this becomes

$$\begin{aligned} \nabla ^2 \Phi - \frac{1}{2} (\textrm{i}\partial \bar{\partial } \omega )_\alpha {}^{\alpha \mu }{}_\mu + \frac{1}{2}|H|^2 - 2 |\nabla \Phi |^2=0~. \end{aligned}$$

We now substitute the equation (5.4) for $i \partial \bar{\partial } \omega$ and (5.12).

5.29 $$\begin{aligned} \nabla ^2 \Phi - \frac{{\alpha ^{\prime }\,}}{8} \textrm{tr}\hspace{2.0pt}|F|^2 + \frac{{\alpha ^{\prime }\,}}{8} \textrm{tr}\hspace{2.0pt}|R^\textrm{H}|^2+ \frac{1}{2}|H|^2 - 2 |\nabla \Phi |^2 \overset{\ {\alpha ^{\prime }\,}^2}{=}\ 0~. \end{aligned}$$

Here we used

$$\begin{aligned} {\alpha ^{\prime }\,}\textrm{tr}\hspace{2.0pt}(g^{\mu \bar{\nu }} R^{\textrm{CH}}{}_{\mu \bar{\nu }})^2 = \mathcal {O}({\alpha ^{\prime }\,}^3)~, \end{aligned}$$

which follows from (5.13). We can now combine identity (5.29) with the trace of the Einstein equation to obtain the dilaton equation. The trace of the Einstein equation (5.26) is

5.30 $$\begin{aligned} R+ 2 \nabla ^2 \Phi -\frac{3}{2} |H|^2 + \frac{{\alpha ^{\prime }\,}}{2} \textrm{tr}\hspace{2.0pt}|F|^2 - \frac{{\alpha ^{\prime }\,}}{2} |R^\textrm{H}|^2 \overset{\ {\alpha ^{\prime }\,}^2}{=}\ 0~. \end{aligned}$$

Add twice equation (5.29) to equation (5.30) to obtain

$$\begin{aligned} R+ 4 \nabla ^2 \Phi - 4 |\nabla \Phi |^2 -\frac{1}{2} |H|^2 + \frac{{\alpha ^{\prime }\,}}{4} \textrm{tr}\hspace{2.0pt}|F|^2 - \frac{{\alpha ^{\prime }\,}}{4} |R^\textrm{H}|^2 \overset{\ {\alpha ^{\prime }\,}^2}{=}\ 0~. \end{aligned}$$

We conclude that the dilaton equation is satisfied to appropriate order.

### Gauge fixing

To complete the proof of Proposition 3, it remains to show that after pulling back by diffeomorphisms flowing along ${\alpha ^{\prime }\,}$, we can assume that the dilaton function $\Phi$ is a constant plus terms of order $\mathcal {O}({\alpha ^{\prime }\,}^2)$. It is automatic that $\Phi = \textrm{const} + \mathcal {O}({\alpha ^{\prime }\,})$, and the task is to upgrade this by gauge fixing to $\Phi = \textrm{const} + \mathcal {O}({\alpha ^{\prime }\,}^2)$. This procedure is well-known in the literature: this particular choice of gauge was used by Witten–Witten [38] and further refined by Anguelova–Quigley–Sethi [17] (see [49] for a summary). To end this section, we give here a self-contained presentation of these known results.

Let *X* be a compact manifold. Suppose we have a smooth family

$$ g_{\alpha ^{\prime }\,}= g^{(0)}+{\alpha ^{\prime }\,}g^{(1)} + {\alpha ^{\prime }\,}^2\,g^{(2)} + \dots ~, \quad J_{\alpha ^{\prime }\,}= J^{(0)} + {\alpha ^{\prime }\,}J^{(1)} + {\alpha ^{\prime }\,}^2 J^{(2)} + \dots ~, $$

parametrized by ${\alpha ^{\prime }\,}\in [0,1)$. Let $\varphi _{\alpha ^{\prime }\,}: X \rightarrow X$ be a 1-parameter family of diffeomorphisms which is the flow of a vector field *v*, so that

$$ \frac{\textrm{d}}{\textrm{d}{\alpha ^{\prime }\,}} \bigg |_{{\alpha ^{\prime }\,}=0} \varphi _{\alpha ^{\prime }\,}= v~, \quad \varphi _0 = id~. $$

The Lie derivative acting on a metric tensor $g^{(0)}$ satisfies

$$ \frac{\textrm{d}}{\textrm{d}{\alpha ^{\prime }\,}} \bigg |_{{\alpha ^{\prime }\,}=0} (\varphi _{\alpha ^{\prime }\,}^* g^{(0)})_{ij} = (L_v g^{(0)})_{ij} = \nabla _i v_j + \nabla _j v_i~, $$

with $\nabla$ the Levi-Civita connection of $g^{(0)}$. If *g* also varies with ${\alpha ^{\prime }\,}$, the formula is

$$ \frac{\textrm{d}}{\textrm{d}{\alpha ^{\prime }\,}} \bigg |_{{\alpha ^{\prime }\,}=0} (\varphi _{\alpha ^{\prime }\,}^* g_{\alpha ^{\prime }\,})_{ij} = L_v g^{(0)} + g^{(1)}~. $$

It follows that altering *g* by a moving reference frame leads to the expansion

$$ \varphi _{\alpha ^{\prime }\,}^* g_{\alpha ^{\prime }\,}= g^{(0)} + {\alpha ^{\prime }\,}(g^{(1)} + L_v g^{(0)}) + \dots ~. $$

Suppose we also have a family of scalar functions $\Phi _{\alpha ^{\prime }\,}= \Phi ^{(0)} + {\alpha ^{\prime }\,}\Phi ^{(1)} + \dots$ which solve

$$ \partial _m \Phi _{\alpha ^{\prime }\,}= \frac{1}{2}g^{ij} (\textrm{d}^c \omega )_{ijm} + \mathcal {O}({\alpha ^{\prime }\,}^2)~, \quad \Phi ^{(0)} = \textrm{const}~. $$

We want to choose the vector field *v* so that $\varphi _{\alpha ^{\prime }\,}^* \Phi _{\alpha ^{\prime }\,}= \Phi ^{(0)} + \mathcal {O}({\alpha ^{\prime }\,}^2)$. We can rewrite the dilaton equation as

$$ \partial _{\rho } \Phi = g^{\mu \bar{\nu }} (\nabla _\mu g_{\rho \bar{\nu }} - \nabla _\rho g_{\mu \bar{\nu }}) + \mathcal {O}({\alpha ^{\prime }\,}^2)~. $$

These holomorphic coordinates move along the deformation, so we write the equation in terms of fixed real coordinates.

$$ \partial _{k} \Phi = g^{ij} \nabla _i g_{k j} - \frac{1}{2}g^{ij} \nabla _k g_{ij} + \mathcal {O}({\alpha ^{\prime }\,}^2)~. $$

If we expand *g* in ${\alpha ^{\prime }\,}$ and set $\partial \Phi ^{(1)}$ to zero, we obtain the condition

5.31 $$\begin{aligned} \nabla ^{i} (g^{(1)})_{i k} - \frac{1}{2}\nabla _k (g^{(1)})_i{}^i = 0~. \end{aligned}$$

Witten–Witten [38] showed that there exists a vector field *v* such that replacing

$$ g^{(1)} \mapsto g^{(1)} + L_v g^{(0)}~, $$

satisfies (5.31). Indeed, the equation for *v* is

5.32 $$\begin{aligned} \nabla ^{i} \nabla _i v_k =\! -\nabla ^{i} (g^{(1)})_{i k} + \frac{1}{2}\nabla _k (g^{(1)})_i{}^i~, \end{aligned}$$

after using $[\nabla _i,\nabla _k] v^i = 0$ by Ricci-flatness. Since $L = \nabla ^i \nabla _i$ is a self-adjoint elliptic operator acting on vector fields, by the theory of elliptic PDE on manifolds the equation is solvable if the right-hand side is $L^2$ orthogonal to $\mathrm{ker ~}L$.

5.33 $$\begin{aligned} L v^k =\! - \nabla _i \bigg ( (g^{(1)})^{ik} + \frac{1}{2} g^{k i} (g^{(1)})_r{}^r \bigg )~. \end{aligned}$$

Integration by parts shows that $\mathrm{ker ~}L = \{ w \in \Gamma (TX): \nabla w = 0 \}$. Pairing the right-hand side with $w \in \mathrm{ker ~}L$ and integrating by parts gives zero, hence the equation for *v* is solvable.

In summary, $\check{\Phi } = \varphi _{\alpha ^{\prime }\,}^* \Phi$ satisfies

$$ \partial \check{\Phi } = 0 + \mathcal {O}({\alpha ^{\prime }\,}^2)~. $$

In fact, [17] showed that *v* can be taken to even satisfy

$$ \partial \check{\Phi } = 0 + \mathcal {O}({\alpha ^{\prime }\,}^3)~, $$

but we will not need this stronger result in the present work.

## Conclusions

### Geometric flows

There is a version of Ricci flow adapted to heterotic string compactifications. The idea is as follows: in practice, one can construct various explicit examples of complex geometries $(X,\Omega ,\omega )$ and bundles $E \rightarrow X$ solving

6.1 $$\begin{aligned} F^{0,2} =0~, \quad d \Omega = 0~, \quad \textrm{d}(\left\| \Omega \right\| _g \omega ^2)= 0~. \end{aligned}$$

There are in a sense intermediate supersymmetric configurations; the full supersymmetry equations (5.3), (5.4) are not satisfied, as we are missing

6.2 $$\begin{aligned} F \wedge \omega ^2 = 0~, \quad \textrm{i}\partial \bar{\partial } \omega = \frac{{\alpha ^{\prime }\,}}{8} [\textrm{tr}\hspace{2.0pt}R^\textrm{CH}\wedge \textrm{tr}\hspace{2.0pt}R^\textrm{CH}- \textrm{tr}\hspace{2.0pt}F \wedge F ]~. \end{aligned}$$

The method then is to start with concrete examples solving the partial constraints (6.1) and deform them along a flow which seeks to reach an on-shell configuration solving the full equations of motion. The anomaly flow, introduced in [50, 51], is a type of Ricci flow which does this: it is a flow that remains in the class of complex geometries solving (6.1) and whose fixed-points solve (6.2). The flow equations are:

6.3 $$\begin{aligned} \partial _t ( \left\| \Omega \right\| _{g(t)} \omega (t)^2)&= \textrm{i}\partial \bar{\partial } \omega (t) - \frac{{\alpha ^{\prime }\,}}{8} ( \textrm{tr}\hspace{2.0pt}R^{\textrm{CH}} \wedge R^\textrm{CH}- \textrm{tr}\hspace{2.0pt}F_{h(t)} \wedge F_{h(t)})~, \end{aligned}$$

6.4 $$\begin{aligned} h^{-1} \partial _t h&=\! - \textrm{i}\Lambda _{\omega (t)} F_{h(t)}~, \end{aligned}$$

where the pair $(\omega (t),h(t))$ is flowing, and the holomorphic volume form $\Omega$ on *X* and complex structure on *E* remains fixed. Here *h*(*t*) is a hermitian metric on the holomorphic bundle *E* with Chern curvature $F_h = \bar{\partial }( h^{-1} \partial h)$ and its flow is given by the Donaldson heat flow [36]. The initial metric $\omega _0$ solves $d ( \left\| \Omega \right\| _{g(0)} \omega (0)^2)=0$ and the equations are such that $d ( \left\| \Omega \right\| _{g(t)} \omega (t)^2)=0$ at all times. The reason this is a type of Ricci flow is because the metric tensor evolves as (see e.g. [52])

$$\begin{aligned} \partial _t g_{\alpha \bar{\beta }} = \frac{e^{2 \Phi }}{2} \bigg [ - \textrm{Ric}_{\alpha \bar{\beta }} - 2 \nabla _\alpha \nabla _{\bar{\beta }} \Phi +\frac{1}{4} H_{\alpha mn} H_{\bar{\beta }}{}^{mn} \bigg ] + \mathcal {O}({\alpha ^{\prime }\,})~. \end{aligned}$$

As the flow is nonlinear, there is a requirement on the scale: $|{\alpha ^{\prime }\,}R_{g(t)}| \ll 1$ must be satisfied for the flow to be mathematically well-defined [50, 53]. Explicit examples of this flow can be tested on $T^4$ fibrations over Riemann surfaces [54] and $T^2$ fibrations over *K*3 surfaces [55]. We note that there is also another version of Ricci flow [56] adapted to heterotic string theory without the gravitational term $\textrm{tr}\hspace{2.0pt}R \wedge R$.

If the flow converges as $t \rightarrow \infty$, then limiting fixed point configurations solve (6.1) and (6.2). Our current work has demonstrated that these equations of complex geometry are consistent with supersymmetry at ${\alpha ^{\prime }\,}^2$ and solve the equations of motion after possibly gauge-fixing, where the fields *H* and $\Phi$ are obtained from the complex geometry via (5.5) and (5.6). It is unclear how the anomaly flow interacts with the gauge fixing condition $\Phi = \textrm{const} +\mathcal {O}({\alpha ^{\prime }\,}^2)$. For the string theoretic study of the anomaly flow at leading order in ${\alpha ^{\prime }\,}$, see [52].

From the mathematical perspective, the major question on the anomaly flow is to understand the class of initial data which leads to long-time existence and convergence; specific explicit examples have been constructed with a wide range of behaviour including finite-time divergence [54] as well as infinite-time convergence [55]. Regardless of the behavior of this particular flow, an outstanding question is to determine when an intermediate configuration solving (6.1) can be deformed to solve the full system including (6.2); the significance of this problem in pure mathematics and differential geometry has been underscored by S.-T. Yau [44, 57].

### Torsional *K*3 solutions

Let us comment on the assumption of a smooth ${\alpha ^{\prime }\,}\rightarrow 0$ limiting metric, which is used throughout our analysis. There are in fact examples in string theory (and not supergravity) that do not satisfy this assumption. But if this ${\alpha ^{\prime }\,}\rightarrow 0$ limit does not produce genuine limiting fields on the threefold, such solutions do not have sigma model descriptions and must be described by other methods such as duality.

Nonetheless, the example of a $T^2$ fibration over a *K*3 receives interest and so we comment very briefly on the set-up. The Calabi-Yau threefold given by a $T^2$ fibered over a *K*3 base was suggested in [58] to have significance in string theory, and it has been widely studied in both string theory and pure mathematics [18, 57, 59–61]. In particular, Fu-Yau [57] constructed solutions to the nonlinear constraint (5.4) for $i \partial \bar{\partial }\omega$ over these geometries.

Let *X* be the total space of a $U(1) \times U(1)$ principal bundle over a *K*3 surface *S*. Let $(\omega _S,\Omega _S)$ be a Calabi-Yau structure on *S*. The hermitian metric and holomorphic volume form on the threefold *X* are:6.5where *a* is a constant denoting the volume of the torus. Here $\phi : S \rightarrow \mathbb {R}$ is a function on the base and  and  is a connection 1-form on each *U*(1)-bundle factor. Flux quantization has the implication that *a* is an integer multiple of $2\pi {\alpha ^{\prime }\,}$ [18]. This setup is such that the metric and Hermitian form on the threefold are not well-defined in the large-radius (i.e., ${\alpha ^{\prime }\,}\rightarrow 0$) limit as the geometry collapses down to the base $(S, e^{2 \phi } \omega _S)$.

If the supergravity and supersymmetry equations studied here are to approximate the string theory coming from the sigma model, one must have a smooth ${\alpha ^{\prime }\,}\rightarrow 0$ limit. As a result, these backgrounds do not correspond to weakly coupled sigma models–linear or non-linear–that flow to conformal field theories. Instead, they are believed to be defined only indirectly, through dualities with type IIB string theory or M-theory. Consequently, without a sigma model to rely on, the ${\alpha ^{\prime }\,}^2$-analysis discussed here is not obviously appropriate to study these geometries. We likely need new computational tools, such as duality to M-theory, and leave this open for future investigation.

### Conclusions

Bergshoeff–de Roo [1] constructed the supersymmetry algebra for the heterotic string and the action it preserves up to and including the ${\alpha ^{\prime }\,}^2$ corrections. Starting from these equations, we analyze the resulting geometry on the compact six-dimensional manifold *X*. We find that *X* is a complex manifold with *SU*(3) holonomy with respect to the Bismut connection, and that *X* is a non-Kähler Calabi-Yau threefold with conformally balanced metric $\omega$ satisfying a nonlinear constraint on $i \partial \bar{\partial } \omega$.

Furthermore, we find the following:

(a) Starting from supersymmetry and the Bianchi identity we prove the equations of motion hold in constant dilaton gauge up to and including ${\alpha ^{\prime }\,}^2$ corrections.

(b) For solutions with a smooth ${\alpha ^{\prime }\,}\rightarrow 0$ limit, we observe $H=\mathcal {O}({\alpha ^{\prime }\,})$, $\nabla \Phi =\mathcal {O}({\alpha ^{\prime }\,})$ and

$$\begin{aligned} \begin{aligned} {\alpha ^{\prime }\,}F^{0,2} + \mathcal {O}({\alpha ^{\prime }\,}^3)&=0~, \quad \textrm{d}\Omega +\mathcal {O}({\alpha ^{\prime }\,}^3) = 0~, \quad \textrm{d}(\left\| \Omega \right\| _g \omega ^2) +\mathcal {O}({\alpha ^{\prime }\,}^3) = 0~,\\ {\alpha ^{\prime }\,}F \wedge \omega ^2 + \mathcal {O}({\alpha ^{\prime }\,}^3)&= 0~, \quad \textrm{i}\partial \bar{\partial } \omega = \frac{{\alpha ^{\prime }\,}}{8} [\textrm{tr}\hspace{2.0pt}R^\textrm{CH}\wedge \textrm{tr}\hspace{2.0pt}R^\textrm{CH}- \textrm{tr}\hspace{2.0pt}F \wedge F ] + \mathcal {O}({\alpha ^{\prime }\,}^3)~. \end{aligned} \end{aligned}$$

We found, remarkably, that these equations when written in terms of the holomorphic structure did not receive ${\alpha ^{\prime }\,}^2$ corrections. Indeed, the zeroth order geometry is Kähler Calabi-Yau, while the first and second order non-Kähler complex geometries match the equations from Strominger’s first order analysis [10].

Supersymmetry also requiresIn constant dilaton gauge, we show $(\textrm{d}H)_\mu {}^\mu = \mathcal {O}({\alpha ^{\prime }\,}^2)$ and so $R^\textrm{H}$ is traceless but still has non-vanishing (0, 2)-component and so is not an instanton.

It is natural to ask how the ${\alpha ^{\prime }\,}$ corrections modify the moduli space. In particular, one could study the corrections to the moduli space’s natural Kähler metric and its associated Kähler potential governing the moduli and matter field dynamics, as constructed in [49, 62, 63]. Based on [64], it is unlikely for the perturbative ${\alpha ^{\prime }\,}$-corrections to modify the dimension of the moduli space and so the modifications to the metric are not going to be topological or dimension changing in nature. In studies of mirror symmetry of Calabi-Yau manifolds, the Kähler potential is modified by an ${\alpha ^{\prime }\,}^3 \zeta (3)$ term. Are there additional terms for heterotic theories? At ${\alpha ^{\prime }\,}^2$, the results here suggest there might be some protection of the moduli space metric and Kähler potential from ${\alpha ^{\prime }\,}^2$ corrections, at least when it is written in terms of deformations of $\Theta ^\textrm{H}$. We hope to report on this in future work. It would be of interest to further investigate the associated universal geometric structures [65, 66] and their capacity to encode the effects of ${\alpha ^{\prime }\,}$-corrections on the background geometry.

The conditions that derive from an action functional (labelled the superpotential see e.g. [43, 63, 67, 68])include $\textrm{d}\Omega = 0$, $F^{0,2} = 0$, $H=\textrm{i}({\partial }-{\overline{\partial }})\omega$ and $R^{0,2} = 0$ . While *W* captures a subset of the first order ${\alpha ^{\prime }\,}$ supersymmetry equations, ${\alpha ^{\prime }\,}R^{0,2}$ is modified at ${\alpha ^{\prime }\,}^2$, which suggests *W* receives corrections. Investigating these issues, and how the finite deformations of *W* described in [68] are affected, would be interesting to pursue as future work.

It would also be of interest to investigate the structure and implications of the ${\alpha ^{\prime }\,}^2$ supersymmetry algebra in the context of compactifications on $\textrm{G}_2$– and $\textrm{Spin}(7)$–holonomy manifolds, as well as on special geometries such as $\textrm{K3} \times T^2$, which preserve extended supersymmetry. For $\textrm{G}_2$ and $\textrm{Spin}(7)$ backgrounds, the geometry is governed by a closed (or co-closed) defining form–either the associative 3-form in the case of $\textrm{G}_2$, or the Cayley 4-form in the $\textrm{Spin}(7)$ case–leading to nontrivial torsion classes when fluxes are introduced. In such settings, the preservation of supersymmetry often requires gauge fields to satisfy an instanton condition, i.e., that their curvature lies in a specific subbundle of two-forms determined by the holonomy. Understanding how this condition is modified in the presence of ${\alpha ^{\prime }\,}$-corrections, and how it is reflected in the structure of the BdR algebra, is an important question for future work.

## Gamma Matrix Results

We list here some useful gamma matrix identities. Our conventions for gamma matrices are $\{ \gamma _m, \gamma _n \} = 2 g_{mn} I$.

We have the following gamma matrix results:

A.1 $$\begin{aligned}&{[}\gamma ^m, \gamma _r] = 2\gamma ^m{}_r~,&\qquad&\{ \gamma ^m, \gamma _r\} = 2\delta ^m{}_r~,\nonumber \\&{[}\gamma ^{mn}, \gamma _r] = -4 \delta ^{[m}{}_r \gamma ^{n]}~,  &   \{ \gamma ^{mn}, \gamma _r \} = 2\gamma ^{mn}{}_r~,\nonumber \\&{[}\gamma ^{mnp}, \gamma _r] = 2\gamma ^{mnp}{}_r ~,  &   \{ \gamma ^{mnp}, \gamma _r \} = 6 \delta ^{[m}{}_r \gamma ^{np]}~, \end{aligned}$$

and

A.2 $$\begin{aligned}&{[}\gamma ^{mn}, \gamma _{rs}] = -8 \delta ^{[m}{}_{[r} \gamma ^{n]}{}_{s]}~,  &   \{ \gamma ^{mn}, \gamma _{rs} \} = 2\gamma ^{mn}{}_{rs} - 4\delta ^{[mn]}{}_{rs}~,\nonumber \\&{[}\gamma ^{mnp}, \gamma _{rs}] = 12 \delta ^{[m}{}_{[r} \gamma ^{np]}{}_{s]}~,  &   \{ \gamma ^{mnp}, \gamma _{rs} \} = 2\gamma ^{mnp}{}_{rs} - 12\delta ^{[mn}{}_{rs}\gamma ^{p]}~. \end{aligned}$$

These results are independent of dimension. We now restrict to an *SU*(3) manifold, so that we work over a 6-dimensional manifold *X*. In this case, we take gamma matrices representing $\textrm{Cliff}(6)$ to be pure imaginary: $\gamma _m^\dagger = \gamma _m$. Let $\eta$ be a pure Weyl spinor, so that with respect to the almost complex structure $J^k{}_\ell = i \eta ^\dagger \gamma ^k{}_\ell \eta$ we have the decomposition  with . If $\mu , \nu$ denotes indices along , then

$$ \gamma ^{\overline{\mu }}\eta = \gamma _\mu \eta = 0~, \quad \{ \gamma ^\mu , \gamma ^{\overline{\nu }}\} = 2\,g^{\mu {\overline{\nu }}}~. $$

Then we have some useful results

A.3 $$\begin{aligned}&\gamma ^{\overline{\mu }}\eta = 0~,&\quad&\gamma ^{\mu {\overline{\nu }}} \eta = -g^{\mu {\overline{\nu }}}\eta ~,&\quad&\gamma ^{{\overline{\mu }}{\overline{\nu }}} \eta = 0~, \nonumber \\&\gamma ^{\mu \nu {\overline{\rho }}} \eta = g^{\mu {\overline{\rho }}} \,\gamma ^\nu \eta - g^{\nu {\overline{\rho }}} \,\gamma ^\mu \eta ~,&\quad&\gamma ^{\mu {\overline{\nu }}{\overline{\rho }}} \eta =0 ~,&\quad&\gamma ^{{\overline{\mu }}{\overline{\nu }}{\overline{\rho }}} \eta = 0~,\nonumber \\&\gamma ^{\mu \nu } \gamma ^\rho \eta = \gamma ^{\mu \nu \rho }\eta ~,&\quad&\gamma ^{\mu {\overline{\nu }}}\gamma ^{\rho } \eta = 2g^{{\overline{\nu }}\rho } \,\gamma ^\mu \eta - g^{\mu {\overline{\nu }}} \,\gamma ^\rho \eta ~,&\quad&\gamma ^{{\overline{\mu }}{\overline{\nu }}} \gamma ^\rho \eta = 0~. \end{aligned}$$

In addition

A.4 $$\begin{aligned} \begin{aligned} \gamma ^{\mu \nu }\gamma ^{\rho \sigma {\overline{\tau }}} \eta&= \left( g^{\rho {\overline{\tau }}} \gamma ^{\mu \nu \sigma } - g^{\sigma {\overline{\tau }}} \gamma ^{\mu \nu \rho } \right) \eta ~, \\ \gamma ^{\mu {\overline{\nu }}}\gamma ^{\rho \sigma {\overline{\tau }}} \eta&= 2(g^{\rho {\overline{\tau }}}g^{\sigma {\overline{\nu }}} - g^{\sigma {\overline{\tau }}} g^{\rho {\overline{\nu }}} )\gamma ^\mu \eta + g^{\mu {\overline{\nu }}} \left( g^{\sigma {\overline{\tau }}} \gamma ^\rho - g^{\rho {\overline{\tau }}} \gamma ^\sigma \right) \eta ~, \\ \gamma ^{{\overline{\mu }}{\overline{\nu }}}\gamma ^{\rho \sigma {\overline{\tau }}} \eta&= 0~. \end{aligned} \end{aligned}$$

## Dictionary of Notation to Bergsehoeff-de Roo

In Appendix A of BdR [1] the action and supersymmetry variations correct to ${\alpha ^{\prime }\,}^2$ are written out. Here we provide a simple dictionary of notation. The left-hand side is always BdR; the right hand side is our notation, largely the same as AQS [17].

B.1 $$\begin{aligned} \begin{aligned}&\phi = e^{2\Phi / 3}~, \qquad \phi ^{-1} {\partial }_m \phi = \frac{2}{3} {\partial }_m \Phi ~, \quad \phi ^{-3} = e^{-2\Phi }~,\\&\omega _{mab} = - \Theta ^\textrm{LC}_{mab}~, \quad \widetilde{R}(\omega ) = - R(\Theta ^\textrm{LC})~, \\&H^{BdR}_{mnp} = \frac{1}{3\sqrt{2}} H_{mnp}~, \quad B^{BdR}_{mn} = \frac{1}{\sqrt{2}} B_{mn}~,\\&\Omega ^\pm _{mab} =\!- \Theta ^\mp _{mab}~, \qquad \alpha = \beta = -\frac{{\alpha ^{\prime }\,}}{4}~,\\&T_{mnpq} = - \frac{1}{3!} (\textrm{d}H)_{mnpq}~,\\&e = \frac{1}{\kappa _{10}^2} = 1~, \end{aligned} \end{aligned}$$

where $\kappa _{10} = 1$ is Newton’s constant and set to unity. Note that the Riemann curvature is defined in BdR (see appendix A of BdR) as

B.2 $$\begin{aligned} \widetilde{R}(\omega )_{mn}{}^{ab} = 2 {\partial }_{[m} \omega _{n]}{}^{ab} - \omega _{[m}{}^{ac} \omega _{n]}{}_c{}^b~, \end{aligned}$$

and so the map is $\widetilde{R}(\omega ) = - R(\Theta ^\textrm{LC})$, where the RHS is defined in a conventional fashion $R(\Theta ^\textrm{LC}) = \textrm{d}\Theta ^\textrm{LC}+ (\Theta ^\textrm{LC})^2$.

The Bergshoeff–de Roo action, once transformed to our notation, is

B.3 $$\begin{aligned} \begin{aligned} \mathcal {L}&= e\phi ^{-3} \left\{ -\frac{1}{2}R(\omega ) - \frac{3}{4} H_{mnp} H^{mnp} + \frac{9}{2} (\phi ^{-1} {\partial }_m \phi )^2 + \cdots \right\} \\&= \frac{e^{-2\Phi }}{2 } \left\{ R(\Theta ^\textrm{LC}) - \frac{1}{2}|H|^2 + 4 ({\partial }_m\Phi )^2 + \frac{{\alpha ^{\prime }\,}}{4} \textrm{tr}\hspace{2.0pt}|F|^2 - \frac{{\alpha ^{\prime }\,}}{4} \textrm{tr}\hspace{2.0pt}|R|^2 + \cdots \right\} ~. \end{aligned} \end{aligned}$$

The field strength *F*, *R* are anti-hermitian with trace negative definite $\textrm{tr}\hspace{2.0pt}T^A T^B = - \delta ^{AB}$. It is useful to note that $\Omega ^{-\,ab} \textrm{d}\Omega ^{-\,ab} = - \textrm{tr}\hspace{2.0pt}\Omega ^- \textrm{d}\Omega ^-$ as $\Omega ^-$ is antisymmetric in its endomorphism indices. We discuss this in subsection to come.

The three-form becomes

B.4 $$\begin{aligned} \begin{aligned} H_{mnp} = 3 {\partial }_{[m} B_{np]} + \frac{{\alpha ^{\prime }\,}}{4} {\textrm{CS}}(A)_{mnp} - \frac{{\alpha ^{\prime }\,}}{4} {\textrm{CS}}(\Theta ^+)_{mnp}~. \end{aligned} \end{aligned}$$

These conventions match Green-Schwarz’s anomaly cancellation paper [2], see below. The gravitino and dilatino variations become (correct to ${\alpha ^{\prime }\,}^2$):

B.5

As written here there is one important difference as compared with AQS: in $P_{mab}$ BdR write the adjoint of $\nabla ^-$ contracted on the first index, not the second index as written in AQS. The normalisation of this term differs slightly, 1/4 here vs 6 in AQS.

### The trace, the action and null energy condition

There is ambiguity in parts of the physics literature surrounding the sign of the trace in the action and Bianchi identity. To that end, we head direct to the source: the Green-Schwarz anomaly cancellation paper [2]. The action is originally presented in the Einstein frame, which we have converted to the string frame and adapted to our notation.^6^

B.6 $$\begin{aligned} \begin{aligned} S = \frac{1}{2} \int e^{-2\Phi } \left\{ R - \frac{1}{12} H_{mnp} H^{mnp} + 4 ({\partial }_m\Phi )^2 - \frac{{\alpha ^{\prime }\,}}{4} F^A_{mn} F^{A\,mn} + \cdots \right\} ~, \end{aligned} \end{aligned}$$

where $F = F^A T^A$ and the generators $T^A$ are antihermitian, so that $F = \textrm{d}A + A^2$ with a similar definition for the spin connection. Anomaly cancellation then requires

B.7 $$\begin{aligned} H = \textrm{d}B + \frac{{\alpha ^{\prime }\,}}{4} CS(A) - \frac{{\alpha ^{\prime }\,}}{4} CS(\Theta )~, \qquad \textrm{d}H = \frac{{\alpha ^{\prime }\,}}{4} \textrm{tr}\hspace{2.0pt}F{\,\wedge \,}F -\frac{{\alpha ^{\prime }\,}}{4} \textrm{tr}\hspace{2.0pt}R{\,\wedge \,}R~. \end{aligned}$$

As the gauge group is compact, one has the possibility of working with a hermitian connection and field strength ${\hat{F}} = d{\hat{A}} - i {\hat{A}}^2$ whose generators can be normalised so that $\textrm{tr}\hspace{2.0pt}T^A T^B$ is positive definite. This would be at the expense of modifying the sign of $\textrm{tr}\hspace{2.0pt}F{\,\wedge \,}F$ in the Bianchi identity (B.7), so that anomaly cancellation continues to hold and we continue to get the topological relation $c_2(X)=c_2(E)$.^7^

We will always work with antihermitian *F* and *R*, and we utilise the principle of BdR [1] that *F* and *R* appear on the same footing. Generators are normalised to $\textrm{tr}\hspace{2.0pt}T^A T^B = - \delta ^{AB}$. After including the curvature term, we get the following action

B.8 $$\begin{aligned} \begin{aligned} S = \frac{1}{2} \int e^{-2\Phi } \left\{ R - \frac{1}{12} H_{mnp} H^{mnp} + 4 ({\partial }_m\Phi )^2 + \frac{{\alpha ^{\prime }\,}}{4} \textrm{tr}\hspace{2.0pt}|F|^2 - \frac{{\alpha ^{\prime }\,}}{4} \textrm{tr}\hspace{2.0pt}|R^\textrm{H}|^2 + \cdots \right\} ~. \end{aligned} \end{aligned}$$

This agrees with the first order action in ${\alpha ^{\prime }\,}$ written down by Bergshoeff–de Roo [1]. Integrating the Bianchi identity we get $c_2(V) = c_2(X)$.

The are some physics calculations we can do to verify the relative sign of $\textrm{tr}\hspace{2.0pt}|F|^2$ in the supergravity action. First, we compute the energy-momentum tensor $T_{mn}$ and check a positive energy theorem from general relativity. The sign of the gauge field contribution must ensure positive energy density. The simplest test is the null energy condition (NEC), requiring:

$$ k^m k^n T_{mn} \ge 0 \quad \mathrm{for\, all\, null\, } k^m \mathrm{with\, } k^m k^n g_{mn} = 0. $$

From Einstein’s equations we can deduce *T*:

B.9 $$\begin{aligned} R_{mn} - \frac{1}{2}g_{mn} R = T_{mn}~. \end{aligned}$$

If we start from the action (B.6), then we get $R_{mn}$ and *R* follow from varying with respect to $g_{mn}$ and the dilaton:

B.10 $$\begin{aligned} \begin{aligned}&R - 4(\nabla \Phi )^2 + 4 \nabla ^2 \Phi - \frac{1}{2}|H|^2 + \frac{{\alpha ^{\prime }\,}}{4} \textrm{tr}\hspace{2.0pt}|F|^2 - \frac{{\alpha ^{\prime }\,}}{4} \textrm{tr}\hspace{2.0pt}|R^\textrm{H}|^2 + \mathcal {O}({\alpha ^{\prime }\,}^3) = 0~,\\&\textrm{Ric}_{mn}+ 2 \nabla _m \nabla _n\Phi - \frac{1}{4} H_{mpq} H_n{}^{pq} +\frac{{\alpha ^{\prime }\,}}{4} \textrm{tr}\hspace{2.0pt}F_{mp} F_n{}^p - \frac{{\alpha ^{\prime }\,}}{4} \textrm{tr}\hspace{2.0pt}R^\textrm{H}_{mp} R_n{}^p + \mathcal {O}({\alpha ^{\prime }\,}^3) = 0~.\\ \end{aligned} \end{aligned}$$

For simplicity, we now set the dilaton to a constant, in which case, there is no difference between Einstein and string frame. We find

B.11 $$\begin{aligned} \begin{aligned} T_{mn}&= - 2\left( \nabla _m \nabla _n\Phi + g_{mn} ((\nabla \Phi )^2 - \nabla ^2 \Phi )\right) + \frac{1}{4} \left( H_{mpq} H_n{}^{pq} - g_{mn} |H|^2\right) \\&\quad -\frac{{\alpha ^{\prime }\,}}{4} \left( \textrm{tr}\hspace{2.0pt}F_{mp} F_n{}^p - \frac{1}{2}g_{mn} \textrm{tr}\hspace{2.0pt}|F|^2\right) +\frac{{\alpha ^{\prime }\,}}{4} \left( \textrm{tr}\hspace{2.0pt}R^\textrm{H}_{mp} R^\textrm{H}_n{}^p - \frac{1}{2}g_{mn} \textrm{tr}\hspace{2.0pt}|R^\textrm{H}|^2\right) ~. \end{aligned} \end{aligned}$$

The NEC $k^m k^n T_{mn} \ge 0$ becomes

B.12 $$\begin{aligned} k^m k^n T_{mn} = \frac{1}{4}\left( H_{mpq} k^m \right) ^2 - \frac{{\alpha ^{\prime }\,}}{4} \textrm{tr}\hspace{2.0pt}(F_{mp} k^m) (F_{n}{}^p k^n) + \frac{{\alpha ^{\prime }\,}}{4} \textrm{tr}\hspace{2.0pt}(R^\textrm{H}_{mp} k^m) (R^\textrm{H}_{n}{}^p k^n)~. \end{aligned}$$

Rewrite in terms of generators of the Lie algebra so that terms are manifestly squares:

B.13 $$\begin{aligned} k^m k^n T_{mn} = \frac{1}{4}\left( H_{mpq} k^m \right) ^2 + \frac{{\alpha ^{\prime }\,}}{4} (F^A_{mp} k^m)^2 - \frac{{\alpha ^{\prime }\,}}{4} (R^{\textrm{H}\,A'}_{mp} k^m)^2~, \end{aligned}$$

where *A* denotes a basis of the gauge group and $A'$ runs over the adjoint of *SO*(9, 1). We see that the first two terms are manifestly positive definite. Note that the sign changed as we evalauted the trace; this confirms we have the correct relative sign in the supergravity action. Furthermore, the $\textrm{tr}\hspace{2.0pt}R^\textrm{H}{\,\wedge \,}R^\textrm{H}$ term contributes a negative energy density. This is consistent with heterotic compactifications that are dual type IIB compactifications (or F-theory), in which the $\textrm{tr}\hspace{2.0pt}R^\textrm{H}{\,\wedge \,}R^\textrm{H}$ term is believed to be mapped to the gravitational contribution of O7-planes and these are believed to have a negative contribution to matter energy density [58]. In turn this maps to an 8-derivative term in M-theory known as $X_8$. Such negative density contributions via higher derivative (equivalently ${\alpha ^{\prime }\,}$) corrections are important also in type IIA and massive type IIA string theory [69, 70], facilitating the existence of flux solutions similar to the *K*3 torsional solutions in heterotic.

Its also worth pointing out the moduli space metric for a compactification to $d=4$, written up [49, 62] is evaluated with the convention that the operator $\textrm{Tr}\hspace{2.0pt}$ has action that is positive definite on antihermitian generators, as is done so in for example [17, 40, 60].^8^ We write down how the metric looks in the usual convention that $\textrm{tr}\hspace{2.0pt}$ is the matrix trace. This metric is derivable just from the action (B.8) and is given by

B.14

This metric governs the kinetic terms of $d=4$ massless scalar fields. Since moduli can mix under diffeomorphisms, the gauge bundle’s contribution should match the form and sign of metric and complex structure moduli terms.

## Hermitian Geometry

### Conformally balanced metrics

Let *X* be a complex manifold with $\textrm{dim}_{\mathbb {C}} X = 3$. The tangent bundle splits as

and we will use Greek indices (e.g. $\mu ,\nu$) to denote directions along . We will sometimes use the notation

$$ \textrm{d}^c =\! - \frac{\textrm{i}}{2} (\partial - \bar{\partial })~, \quad \textrm{d}\textrm{d}^c = \textrm{i}\partial \bar{\partial }~. $$

Let $\omega = \textrm{i}g_{\mu \bar{\nu }} \, dx^{\mu \bar{\nu }} \in \Omega ^{1,1}(X,\mathbb {R})$ be a hermitian metric, so that the components satisfy $\overline{g_{\mu \bar{\nu }}} = g_{\nu \bar{\mu }}$ and $g_{\mu \bar{\nu }}$ is a positive-definite local matrix. The components of a differential form in holomorphic coordinates are given by

$$ \eta = \frac{1}{p! q!} \eta _{\mu _1 \cdots \mu _p \bar{\nu }_1 \cdots \bar{\nu }_q} dx^{\mu _1 \cdots \mu _p \bar{\nu }_1 \cdots \bar{\nu }_q}, \quad \eta \in \Omega ^{p,q}(X)~. $$

Roman indices (e.g. *i*, *j*) are used full indices spanning all of $T_{\mathbb {C}} X$. Our conventions for norms of forms are:

$$ |\eta |^2 = \frac{1}{k!} \eta _{i_1 \cdots i_k} \eta ^{i_1 \cdots i_k}, \quad \eta \in \Omega ^k(X)~. $$

We use the notation $i \Lambda _\omega : \Omega ^{p+1,q+1}(X) \rightarrow \Omega ^{p,q}(X)$ for metric contraction:

$$ (i \Lambda _\omega \eta )_K = g^{\mu \bar{\nu }} \eta _{\mu \bar{\nu } K}, \quad K = \mu _1 \cdots \mu _{p} \bar{\nu }_1 \cdots \bar{\nu }_{q}~. $$

In complex dimension 3, we note the following wedge product identity

C.1 $$\begin{aligned} \eta \wedge \omega = \frac{1}{2}(\Lambda _\omega \eta ) \wedge \omega ^2, \quad \eta \in \Omega ^3(X)~. \end{aligned}$$

We now discuss the conformally balanced condition, which generalizes the Kähler condition on $\omega$. Let *f* be an arbitrary function and consider

$$ \partial (e^{-2 f} \omega ^2) = 2 e^{-2 f} \bigg [ \partial \omega \wedge \omega - \partial f \wedge \omega ^2 \bigg ]~. $$

Substitute (C.1) with $$\eta = \partial \omega $$.

C.2 $$\begin{aligned} \partial (e^{-2 f} \omega ^2) = 2 e^{-2 f} \bigg [ -\frac{1}{2}(i \Lambda _\omega ) (i \partial \omega ) - \partial f \bigg ] \wedge \omega ^2~. \end{aligned}$$

We see that

C.3 $$\begin{aligned} \bigg [ d ( e^{-2f} \omega ^2) = 0 \bigg ] \ \Leftrightarrow \ \bigg [ (\partial - \bar{\partial }) f =\! - \frac{\textrm{i}}{2} \Lambda _\omega T \bigg ] \ \Leftrightarrow \ \bigg [ T_\mu {}^\mu {}_\lambda =\! -2 \partial _\lambda f \bigg ]~, \end{aligned}$$

where we use the notation $T \in \Omega ^3(X)$ for

$$ T = \textrm{i}(\partial - \bar{\partial })\omega ~, \quad T_{\mu \nu \bar{\rho }} =\! - \partial _\mu g_{\nu \bar{\rho }} + \partial _\nu g_{\mu \bar{\rho }}~. $$

Metrics solving any of the equivalent equations in (C.3) are said to be conformally balanced.

### C.2. Connection Symbols

Given a hermitian manifold (*X*, *g*), we can ask whether there is an optimal natural connection $\nabla$ on $T_{\mathbb {C}} X$. Unless *g* is a Kähler metric, there is no agreed-upon optimal connection. We present here several connections which all play various roles in string theory and in the main text. Our conventions are as follows: a vector field $V \in \Gamma (T_{\mathbb {C}} X)$ is written as

$$ V = V^k \partial _k = V^\mu \partial _\mu + V^{\bar{\mu }} \partial _{\bar{\mu }}~, $$

where *k* denotes $T_{\mathbb {C}} X$ indices and $\mu$ denotes  indices. Our notation for a connection $\nabla$ acting on $V \in \Gamma (T_{\mathbb {C}} X)$ is

where $\Gamma _i{}^k{}_j$ will be specified by the connection under consideration. It what follows, we only specify $\Gamma _\mu {}^k{}_j$ with the understanding that $\Gamma _{\bar{\mu }}{}^{\bar{k}}{}_{\bar{j}} = \overline{\Gamma _\mu {}^k{}_j}$.

#### Levi-Civita

The Levi–Civita connection $\nabla ^{\textrm{LC}}$ is the unique metric compatible connection with no torsion. Unfortunately, $\nabla ^{\textrm{LC}}$ does not preserve  (unless *g* is Kähler) and is not recommended for calculations in holomorphic coordinates.

C.4 $$\begin{aligned} \begin{aligned}&\Gamma ^{\textrm{LC}}{}_{\mu }{}^{\nu }{}_\rho = g^{\nu {\overline{\sigma }}} \, {\partial }_\mu g_{\rho {\overline{\sigma }}} - \frac{1}{2} T_{\mu }{}^{\nu }{}_{\rho } ~,\\&\Gamma ^{\textrm{LC}}{}_{\mu }{}^{{\overline{\nu }}}{}_{\rho } = 0 ~,\\&\Gamma ^{\textrm{LC}}{}_{\mu }{}^{\nu }{}_{{\overline{\rho }}} = \frac{1}{2} T_{\mu }{}^\nu {}_{{\overline{\rho }}} ~,\\&\Gamma ^{\textrm{LC}}{}_{\mu }{}^{{\overline{\nu }}}{}_{{\overline{\rho }}} =\! -\frac{1}{2} T_{\mu }{}^{{\overline{\nu }}}{}_{{\overline{\rho }}} ~. \end{aligned}\end{aligned}$$

#### Chern

The Chern connection is the unique connection which satisfies both $\nabla _k g_{\mu \bar{\nu }} = 0$ and $\nabla _{\bar{\mu }} V^\alpha = \partial _{\bar{\mu }} V^\alpha$, making it well-suited for calculations in holomorphic coordinates.

C.5 $$\begin{aligned} \begin{aligned}&\Gamma ^{\textrm{CH}}{}_{\mu }{}^{\nu }{}_\rho = g^{\nu {\overline{\sigma }}} \, {\partial }_\mu g_{\rho {\overline{\sigma }}} ~,\\&\Gamma ^{\textrm{CH}}{}_{\mu }{}^{{\overline{\nu }}}{}_{\rho } = 0 ~,\\&\Gamma ^{\textrm{CH}}{}_{\mu }{}^{\nu }{}_{{\overline{\rho }}} = 0 ~,\\&\Gamma ^{\textrm{CH}}{}_{\mu }{}^{{\overline{\nu }}}{}_{{\overline{\rho }}} = 0 ~. \end{aligned}\end{aligned}$$

#### Hull

A connection $\Gamma ^{\textrm{H}}$ appears in the heterotic action and Bianchi identity which we will refer to as the Hull connection. At the order in ${\alpha ^{\prime }\,}$ considered in this paper it is given by $\Gamma _m^{\textrm{H}}= \Gamma _m^{\textrm{LC}} + \frac{1}{2}T_m$. This connection $\Gamma ^{{\textrm{H}}}$ does not preserve  and its connection symbols are:

C.6 $$\begin{aligned} \begin{aligned}&\Gamma ^{{\textrm{H}}}{}_{\mu }{}^{\nu }{}_\rho = \Gamma ^{\textrm{CH}}{}_{\mu }{}^{\nu }{}_\rho ~,\\&\Gamma ^{{\textrm{H}}}{}_{\mu }{}^{{\overline{\nu }}}{}_{\rho } = 0 ~,\\&\Gamma ^{{\textrm{H}}}{}_{\mu }{}^{\nu }{}_{{\overline{\rho }}} = T_\mu {}^\nu {}_{{\overline{\rho }}} ~,\\&\Gamma ^{{\textrm{H}}}{}_{\mu }{}^{{\overline{\nu }}}{}_{{\overline{\rho }}} = 0 ~. \end{aligned}\end{aligned}$$

#### Bismut

It was noticed by Yano [71] that specifying the ansatz $\Gamma _m^{\textrm{B}}= \Gamma _m^{\textrm{LC}} - \frac{1}{2}T_m$ where $T \in \Omega ^3(X)$ is a priori an arbitrary 3-form, together with the requirement that $\nabla ^{\textrm{B}}$ preserves , uniquely determines the 3-form in the ansatz as $T = i (\partial -\bar{\partial })\omega$. See Proposition 1 for details. This connection in non-Kähler complex geometry is sometimes called the Bismut connection.

C.7 $$\begin{aligned} \begin{aligned}&\Gamma ^{{\textrm{B}}}{}_{\mu }{}^{\nu }{}_\rho = \Gamma ^{\textrm{CH}}{}_\mu {}^\nu {}_\rho - T_{\mu }{}^{\nu }{}_{\rho } ~,\\&\Gamma ^{{\textrm{B}}}{}_{\mu }{}^{{\overline{\nu }}}{}_{\rho } = 0 ~,\\&\Gamma ^{{\textrm{B}}}{}_{\mu }{}^{\nu }{}_{{\overline{\rho }}} = 0 ~,\\&\Gamma ^{{\textrm{B}}}{}_{\mu }{}^{{\overline{\nu }}}{}_{{\overline{\rho }}} =\! - T_{\mu }{}^{{\overline{\nu }}}{}_{{\overline{\rho }}} ~. \end{aligned}\end{aligned}$$

### Curvature

From a connection $\nabla$ on $T_{\mathbb {C}} X$, the curvature tensor $R \in \Omega ^2( \textrm{End} \, T_{\mathbb {C}} X)$ will be denoted

From here, one can compute the curvatures of all the connections listed above in holomorphic coordinates. The Ricci curvature and scalar curvature of the Levi-Civita connection are denoted

The 4-form $\textrm{tr}\hspace{2.0pt}R \wedge R$ for various connections will be used in the main text, which is

The coordinate expressions for the Chern curvature are simplest, and will often be used as reference.

One can take four different traces of $R^{\textrm{CH}}$ along the holomorphic directions to get four analogs of Ricci curvature. Two of these traces will play a role in the main text.

1. The first trace is
  C.8 $$\begin{aligned} R^{\textrm{CH}}{}_{\mu \bar{\nu }}{}^\alpha {}_\alpha =\! - \partial _\mu \partial _{\bar{\nu }} \log \det g_{\rho \bar{\sigma }}~. \end{aligned}$$
  Its significance is that it defines the first Chern class. If *X* admits a holomorphic volume form $\Omega$, then
  C.9 $$\begin{aligned} R^{\textrm{CH}}{}_{\mu \bar{\nu }}{}^\alpha {}_\alpha = \partial _\mu \partial _{\bar{\nu }} \log |\Omega |^2_\omega ~. \end{aligned}$$
  Here $\Omega \overset{\textrm{loc}}{=}\ f(x) dx^{123}$ in local holomorphic coordinates $x^\mu$ with *f*(*x*) a local non-vanishing holomorphic function, and $|\Omega |^2_\omega \overset{\textrm{loc}}{=} |f|^2 (\det g_{\mu \bar{\nu }})^{-1}$.
2. The second trace that will appear in our analysis is
  This quantity vanishes if the Chern connection is an instanton. We can compare both traces via the exchange relation
  C.10 $$\begin{aligned} R^{\textrm{CH}}{}_\mu {}^\mu {}_{\bar{\beta } \alpha } = R^{\textrm{Ch}}{}_{\alpha \bar{\beta }}{}^\mu {}_\mu - (i \partial \bar{\partial } \omega )_\mu {}^\mu {}_{\bar{\beta } \alpha } + \nabla _\alpha ^{\textrm{Ch}} T_\mu {}^\mu {}_{\bar{\beta }} - \nabla _{\bar{\beta }}^{\textrm{Ch}} T_\mu {}^\mu {}_\alpha + T_{\alpha \mu \bar{\nu }} T^{\mu \bar{\nu }}{}_{\bar{\beta }}~. \end{aligned}$$

We end with a lemma containing an identity which will be used in the main text.

#### Proposition 4

Let *X* be a complex threefold with hermitian metric $\omega$ and holomorphic volume form $\Omega$. Suppose

$$ \textrm{d} (\left\| \Omega \right\| _g \omega ^2) =0~. $$

Then

C.11 $$\begin{aligned} R^{\textrm{CH}}{}_\mu {}^\mu {}_{\bar{\beta } \alpha }&=\! - (\textrm{i}\partial \bar{\partial } \omega )_\mu {}^\mu {}_{\bar{\beta } \alpha } + T_{\alpha \mu \bar{\nu }} T^{\mu \bar{\nu }}{}_{\bar{\beta }}~, \end{aligned}$$

C.12 $$\begin{aligned} g^{\mu \bar{\nu }} R^{{\textrm{H}}}{}_{\mu \bar{\nu } ij}&=\! - (\textrm{i}\partial \bar{\partial } \omega )_\mu {}^\mu {}_{ij}~, \quad R^{{\textrm{H}}}{}_{\mu \nu ij} = - (\textrm{i}\partial \bar{\partial } \omega )_{\mu \nu ij}~. \end{aligned}$$

Identity (C.11) follows from (C.10) upon substituting (C.3) and (C.8). Identity (C.12) then follows from converting Hull to Chern via (C.6). The interpretation of (C.12) is the deviation of the Hull connection of a conformally balanced metric from being an instanton. We refer to (2.8) for this identity on $R^{{\textrm{H}}}$ derived from the BdR supersymmetry algebra with identification $dH = -2 \textrm{i}\partial \bar{\partial } \omega + \mathcal {O}({\alpha ^{\prime }\,}^2)$.

## Footnotes

1 In [1] there are two coupling constants for the *F* and $R^\textrm{H}$ curvatures, which from supergravity perspective can be different. In string theory the Green-Schwarz anomaly cancellation fixes them in terms of ${\alpha ^{\prime }\,}$.

2 This precludes the torsional $T^2\rightarrow K3$ examples, whose metric degenerates in this limit. A corresponding non-linear sigma model in the ${\alpha ^{\prime }\,}\rightarrow 0$ effectively decouples fields describing the torus from the rest of the geometry. This is problematic with, for example, cancelling the Weyl anomaly as the geometry becomes effectively 8-dimensional. The infrared limit and meaning of the sigma model in this case is obscure, at best.

3 The exception to this is the standard embedding in which $H=0$.

4 In fact [1] make progress in the quartic action.

5 Other choices are possible, for example in their prior paper [4], but complicate the supersymmetry algebra and do not allow for an easy analysis of the ${\alpha ^{\prime }\,}$-corrections. This echoes a point made in [18]: one could find a field redefinition to evaluate $\mathcal {L}(R^2)$ using the Chern connection, but in order to preserve supersymmetry it would require redefinitions of *G*, *B* etc so that what becomes the metric is no longer a tensor–it acquires gauge and gerbe transformation properties from the B-field–and any geometry is at best obscure.
